# Systematic dissection of phenotypic, functional, and tumorigenic heterogeneity of human prostate cancer cells

**DOI:** 10.18632/oncotarget.4260

**Published:** 2015-06-24

**Authors:** Xin Liu, Xin Chen, Kiera Rycaj, Hsueh-Ping Chao, Qu Deng, Collene Jeter, Can Liu, Sofia Honorio, Hangwen Li, Tammy Davis, Mahipal Suraneni, Brian Laffin, Jichao Qin, Qiuhui Li, Tao Yang, Pamela Whitney, Jianjun Shen, Jiaoti Huang, Dean G. Tang

**Affiliations:** ^1^ Department of Epigenetics and Molecular Carcinogenesis, University of Texas MD Anderson Cancer Center, Science Park, Smithville, TX 78957, USA; ^2^ Cancer Stem Cell Institute, Research Center for Translational Medicine, East Hospital, Tongji University School of Medicine, Shanghai 200120, China; ^3^ Program in Molecular Carcinogenesis, University of Texas Graduate School of Biomedical Sciences (GSBS), Houston, TX 77030, USA; ^4^ Department of Pathology and Laboratory Medicine, David Geffen School of Medicine, UCLA, Los Angeles, CA 90095, USA; ^5^ Centers for Cancer Epigenetics, Stem Cell and Developmental Biology, RNA Interference and Non-Coding RNAs, and Molecular Carcinogenesis, University of Texas MD Anderson Cancer Center, Houston, TX 77030, USA

**Keywords:** prostate cancer, cancer stem cells, stem cells, differentiation, heterogeneity

## Abstract

Human cancers are heterogeneous containing stem-like cancer cells operationally defined as cancer stem cells (CSCs) that possess great tumor-initiating and long-term tumor-propagating properties. In this study, we systematically dissect the phenotypic, functional and tumorigenic heterogeneity in human prostate cancer (PCa) using xenograft models and >70 patient tumor samples. In the first part, we further investigate the PSA^−/lo^ PCa cell population, which we have recently shown to harbor self-renewing long-term tumor-propagating cells and present several novel findings. We show that discordant AR and PSA expression in both untreated and castration-resistant PCa (CRPC) results in AR^+^PSA^+^, AR^+^PSA^−^, AR^−^PSA^−^, and AR^−^PSA^+^ subtypes of PCa cells that manifest differential sensitivities to therapeutics. We further demonstrate that castration leads to a great enrichment of PSA^−/lo^ PCa cells in both xenograft tumors and CRPC samples and systemic androgen levels dynamically regulate the relative abundance of PSA^+^ versus PSA^−/lo^ PCa cells that impacts the kinetics of tumor growth. We also present evidence that the PSA^−/lo^ PCa cells possess distinct epigenetic profiles. As the PSA^−/lo^ PCa cell population is heterogeneous, in the second part, we employ two PSA^−^ (Du145 and PC3) and two PSA^+^ (LAPC9 and LAPC4) PCa models as well as patient tumor cells to further dissect the clonogenic and tumorigenic subsets. We report that different PCa models possess distinct tumorigenic subpopulations that both commonly and uniquely express important signaling pathways that could represent therapeutic targets. Our results have important implications in understanding PCa cell heterogeneity, response to clinical therapeutics, and cellular mechanisms underlying CRPC.

## INTRODUCTION

Cellular heterogeneity represents an omnipresent feature in human tumors, which contain cells with diverse morphology, cytogenetic markers, growth kinetics, immunological characteristics, metastatic ability, and sensitivity to therapeutics [1]. Clonal evolution, driven by genetic instability of tumor cells, and phenotypic maturation and diversification, driven by cancer stem cells (CSCs), operate hand-in-hand to generate tumor cell heterogeneity [2]. Specifically, clonal evolution creates genetic diversity and drives clonal competition between multiple subclones in the tumor whereas CSC-directed differentiation and maturation generates phenotypic diversity within individual subclones [2].

One of the key biological properties of CSCs is the ‘stemness’, which confers on a subpopulation of cancer cells two fundamental traits of normal stem cells, i.e., self-renewal and differentiation ability. Like normal stem cells, whose self-renewal and multi-lineage differentiation (i.e., pluripotency) are regulated by an intricate network of transcription factors, CSC stemness is also bestowed by critical signaling pathways (e.g., Notch, HH, and Wnt) and transcription factors and epigenetic regulators such as Nanog, Bmi-1, and Polycomb proteins [3–5]. It has now become clear that intra-clonally, genetic mutations, epigenetic changes and tumor microenvironment converge on regulating the CSC stemness to generate the phenotypic diversity and functional heterogeneity of tumor cells [2].

Many different experimental strategies and approaches have been adopted and developed to purify and enrich CSC populations. These include cell surface marker-based flow sorting, marker-independent strategies such as holoclone, clonogenic sphere formation and label-retaining assays, functional assays such as Side Population (SP; which measures the drug-effluxing ability in CSCs) and Aldefluor assay (which measures the aldehyde dehydrogenase [ALDH] mediated detoxification capability), and *in vitro* and *in vivo* lineage tracing assays [1]. To study the stemness properties, a ‘gold-standard’ functional assay is to xenotransplant candidate human CSC populations in immunodeficient mice at decreasing cell doses, an assay often called limiting dilution (tumor) assay or LDA [1]. The LDA measures tumor-regenerating or tumor-initiating capacity, which, when combined with serial tumor transplantations, would measure the self-renewal ability of the candidate CSCs [1].

Prostate cancer (PCa) is extremely heterogeneous but the cellular basis for PCa cell heterogeneity remains largely unknown. Understanding PCa cell heterogeneity is of clear clinical importance as it likely underlies differential PCa cell response to androgen-deprivation therapy (ADT) and other therapeutics such as docetaxel and helps explain PCa recurrence and metastasis. Work from our lab in the past 10 years has generated important clues to understanding the cellular heterogeneity of PCa. We have demonstrated that PCa cell SP and holoclones, as well as CD44^+^ and CD44^+^α2β1^+^ subpopulations in some PCa models are enriched in prostate CSCs (PCSCs) with high tumorigenic and metastatic potential [6–12]. Using a PSA promoter (PSAP) driven EGFP lentiviral tracing reporter, we have recently provided evidence that the undifferentiated (PSA^−/lo^) PCa cell population harbors long-term tumor-propagating PCSCs that preferentially express stem cell-associated genes and can self-renew to generate PSA^+^ PCa cells by asymmetric cell division [13]. Of clinical significance, PSA^−/lo^ PCa cells can initiate robust tumor regeneration in fully castrated hosts, survive androgen deprivation, and mediate tumor recurrence [13]. Many other groups have also reported PCSC subpopulations [14–24].

One of the issues in PCSC studies is that different research groups often use divergent PCa models and different phenotypic markers or experimental approaches to enrich for putative PCSCs, making direct comparison of the results difficult. The main goals of our current study are to systematically dissect the PCa cell heterogeneity via assessing a spectrum of PCa cell line and xenograft models as well as primary tumor cells and samples, to address the relationship between and among different PCSC subpopulations, and dissect the relationship between PCSCs and AR, PSA, and castration resistance. The results presented here greatly advance our understanding of PCa cell heterogeneity and help to illuminate cellular mechanisms of PCa therapy resistance.

## RESULTS

### PCa cell heterogeneity: Inverse correlation between tumor *PSA* mRNA levels with clinical parameters and discordant *AR* and *PSA* mRNA expression in PCa samples

**Figure 1:**
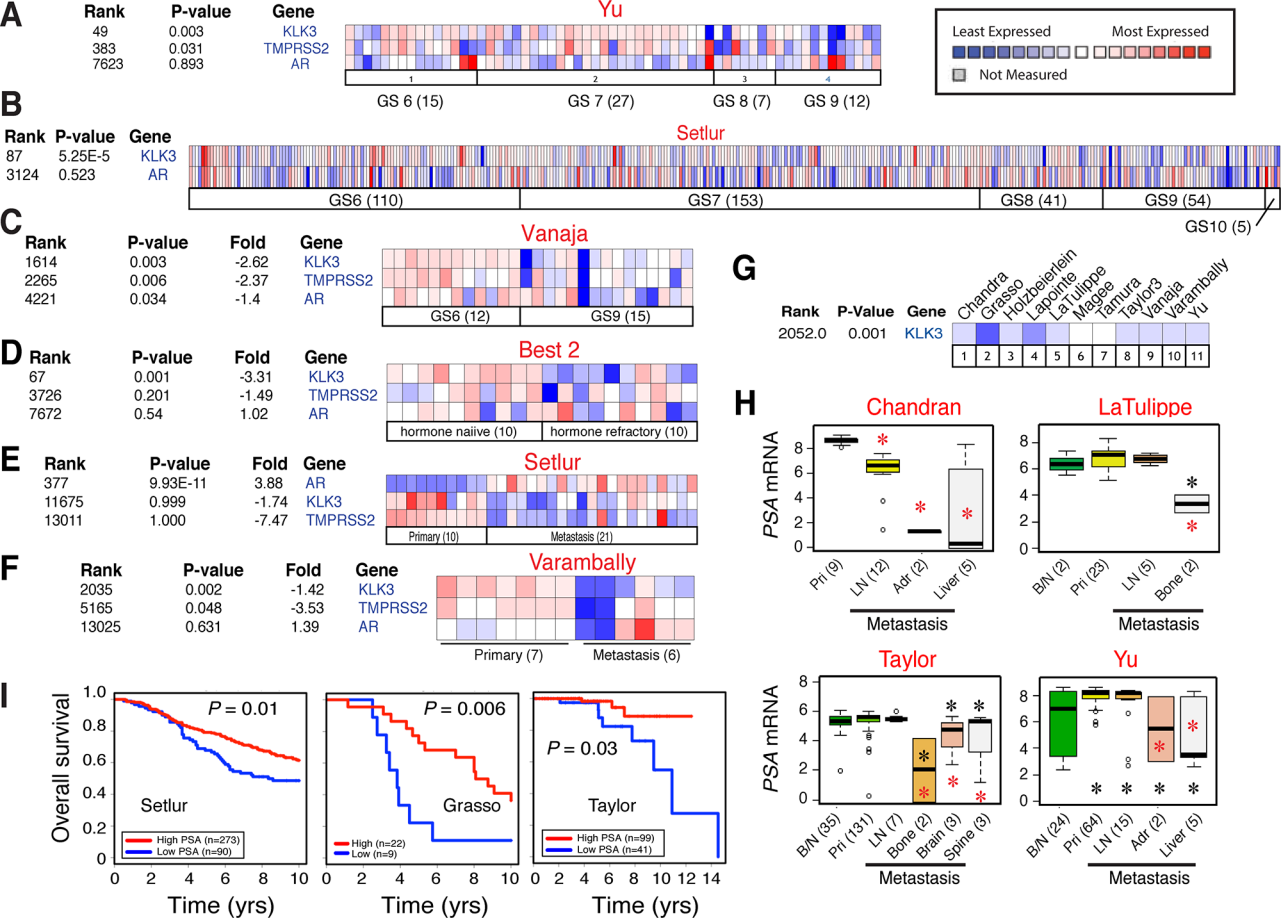
Inverse correlation between tumor *PSA* mRNA levels and clinical parameters **A–C.** Heat map presentation of the mRNA levels of *PSA*, *AR*, and/or *TMPRSS2* in relation to tumor grade (GS, Gleason score) in three representative *Oncomine* data sets (indicated above; see Supplementary Table 1 for information). Note that in individual samples, the *AR* and *PSA* expression patterns are frequently discordant. The legend on the right applies to all heat maps. **D–F.** Heat map showing discordant *AR* and *PSA* expression and reduced *PSA* mRNA levels in CRPC (D) and/or in metastases (E–F). **G.** Reduced *PSA* mRNA levels in PCa metastases across all 11 eligible data sets. **H.** Examples of reduced *PSA* mRNA levels in PCa metastasis. B/N, benign/normal; pri, primary tumor; LN, lymph node; Adr., adrenal gland. Red asterisk, *P* < 0.05 in comparison with primary tumors; black asterisk, *P* < 0.05 compared to B/N samples. **I.** Overall patient survival positively correlates with high *PSA* mRNA levels in 3 data sets.

We started our studies by systematically analyzing 27 ‘eligible’ *Oncomine* data sets of PCa cDNA microarrays (Supplementary Table 1) and by correlating tumor *PSA* mRNA levels versus Gleason grade, hormone-refractory and metastatic status, and patient survival. The results revealed several interesting points. FIRST, an inverse correlation was observed between tumor *PSA* mRNA and tumor grade in all data sets with information on *PSA* mRNA and Gleason grade of the tumors and with sufficient number of cases (Figure 1A–1C; 13). Reduced *PSA* mRNA was also noted in high-grade (i.e., Gleason 8–10) tumors in the data sets of Best 2, Holzbeierlein, and Wallace (not shown). SECOND, reduced *PSA* levels were observed in hormone-refractory PCa in data sets of Best 2 (Figure 1D), and of Tamura and Tomlins (not shown). THIRD, we observed reduced tumor *PSA* mRNA in PCa metastases in all 11 data sets that contained ≥ 5 metastatic samples (Figure 1E–1H). Interestingly, although the draining lymph node (LN) only occasionally showed reduced *PSA* mRNA (e.g., in the Chandran data set; Figure 1H), distant metastases, e.g., those to the adrenal gland, bone, and liver, generally exhibited consistent reduction in *PSA* mRNA (Figure 1H). Distant metastases also tended to express lower *PSA* mRNA than the benign/normal (B/N) tissues (Figure 1H). FINALLY, overall patient survival correlated with high intra-tumoral PSA mRNA levels in the data sets of Nakagawa [13], Setlur, Grasso, and Taylor (Figure 1I).

Strikingly, we frequently observed a discordant relationship between *PSA* and *AR* in individual primary (Figure 1A–1C), hormone-refractory (Figure 1D) and metastatic (Figure 1E–1F) samples. *PSA* mRNA was decreased across all data sets (Figure 1A–1F; data not shown) except the Setlur data set in which *PSA* reduction was not statistically significant although the decreasing trend was clear (Figure 1E). Another AR target, *TMPRSS2*, was also reduced in most data sets analyzed (Figure 1A, 1C–1F; 13). In contrast, the *AR* mRNA levels were not correlated with tumor grade, hormone refractoriness, or metastasis (Figure 1A–1F; 13; data not shown). In one data set (Vanaja), the *AR* mRNA levels were actually decreased in Gleason 9 tumors compared to Gleason 6 tumors (Figure 1C).

### PCa cell subtypes in untreated patient tumors, enrichment of PSA^−/lo^ PCa cells in CRPC and castration-resistant xenograft tumors, and differential drug responses in PCa cell subtypes

**Figure 2:**
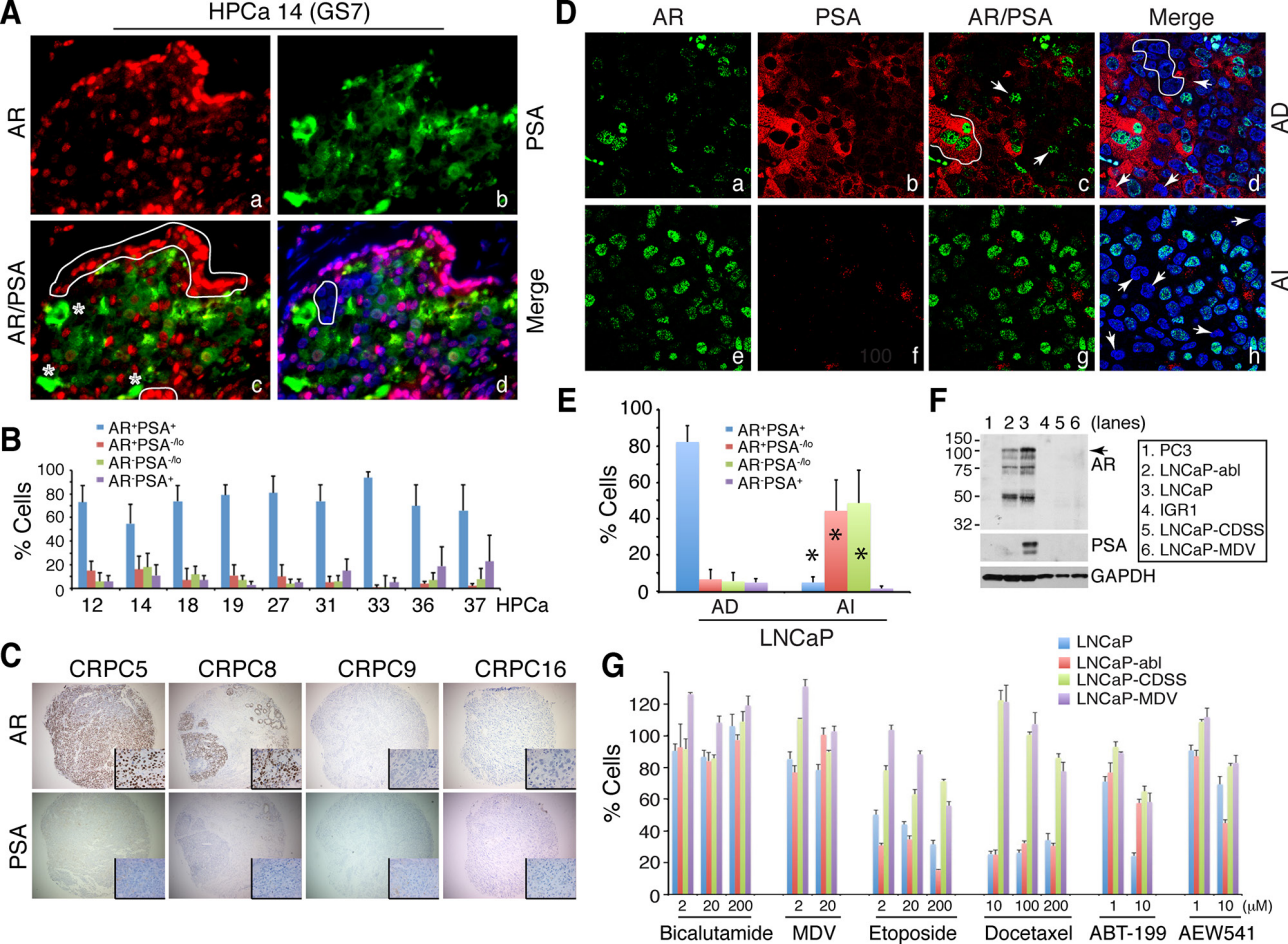
Discordant PSA and AR protein expression in PCa, 4 subtypes of PCa cells, enrichment of PSA^−/lo^ PCa cells in CRPC, and differential drug responses in subtypes of PCa cells **A–B.** Representative immunofluorescence images (×400) illustrating discordant PSA and AR protein expression in HPCa 14 (A) and quantification of 4 subpopulations of PCa cells in the 9 HPCa samples (B). In A, AR^+^PSA^+^ PCa cells are marked by red nuclei and green cytosplasm, AR^+^PSA^−/lo^ cells by red alone (panel c, white circled areas), AR^−^PSA^+^ cells by green alone (panel c, asterisks), and AR^−^PSA^−/lo^ cells by being negative (or low) for both red and green staining (panel d, white circled area). **C.** IHC analysis of AR and PSA in the TMA samples. Shown are 4 CRPC samples illustrating homogeneous loss of PSA in all 4 samples and heterogeneous expression of AR (insets: 400×). **D.** Double immunofluorescence staining of AR and PSA in AD vs. AI LNCaP xenograft tumors. In panel c, the white line demarcates 3 AR^+^PSA^+^ cells and the arrows point to 2 AR^+^PSA^−/lo^ cells. In panel d, the white circle demarcates several AR^−^PSA^−/lo^ cells and the arrows point to 3 AR^−^PSA^+^ cells. In panel h, the arrows illustrate several AR^−^PSA^−/lo^ cells. Shown are representative confocal images (original magnification; ×400). **E.** Quantification of the 4 subtypes of PCa cells in AD and AI LNCaP xenograft tumors. A total of 809 and 907 cells were counted from several AD and AI tumors, respectively. *P < 0.001 in AI compared in AD tumors. **F.** Western blotting analysis of AR and PSA. PC3 and IGR1 cells, which are known to be negative for both proteins, were used as controls. Note that the wild-type LNCaP cells (lane 3) were AR^+^PSA^+^ whereas LNCaP-abl cells AR^+^PSA^−^ (lane 2). LNCaP-CDSS and LNCaP-MDV cells were both AR^−^PSA^−^ (lanes 5–6). The arrow indicates the ∼114 kD full-length AR and lower bands might represent AR splice variants (top panel). **G.** Drug responses in subtypes of LNCaP cells. LNCaP (AR^+^PSA^+^), LNCaP-abl (AR^+^PSA^−^), and LNCaP-CDSS and LNCaP-MDV (AR^−^PSA^−^) cells were treated with the drugs at the indicated concentrations for 72 h. Relative cell numbers were determined by Alamar Blue assays (see Methods). For Bicalutamide, at 2 and 20 μM, only LNCaP-MDV cells showed partial resistance (*P* < 0.05). At 200 μM, Bicalutamide even slightly promoted wild-type LNCaP cell growth probably due to its well-known agonist effects. For MDV3100, at 2 μM, LNCaP-CDSS and LNCaP-MDV but not LNCaP-abl cells showed partial resistance (*P* < 0.05). At 200 μM of MDV, all 3 LNCaP sublines showed partial resistance (*P* < 0.05) in comparison to wild-type cells. Note prominent resistance in LNCaP-CDSS and LNCaP-MDV cells to etoposide and docetaxel (*P* < 0.001 for all comparisons between these two cell types vs. either wild-type or LNCaP-abl cells). All LNCaP cell types responded similarly to 1 μM ABT-199 but the 3 LNCaP sublines (LNCaP-abl, -CDSS, and –MDV) showed common resistance to 20 μM ABT-199 (*P* < 0.01). LNCaP-CDSS and LNCaP-MDV but not LNCaP-abl cells showed partial resistance to 1 μM of AEW541 (*P* < 0.05) and this resistance phenotype dissipated at 10 μM AEW541, at which LNCaP-abl cells showed higher sensitivity than wild-type cells (*P* < 0.05).

Discordant mRNA expression patterns between *AR* and *PSA* suggest 4 subpopulations of PCa cells, i.e., AR^+^PSA^+^, AR^−^PSA^+^, AR^+^PSA^−^, and AR^−^PSA^+^ cells. Immunofluorescence (IF) analysis of AR and PSA proteins in 11 untreated primary patient tumors (HPCa; Supplementary Table 2) directly supports this premise as the 4 subpopulations of PCa cells could be identified in all samples, although, as expected, the AR^+^PSA^+^ PCa cells represented the major subpopulation (Figure 2A–2B; Supplementary Figure 1 and 2). In these analyses, AR showed typical nuclear staining with a spectrum of intensities (negative, weak, intermediate, and strong) whereas PSA generally showed cytoplasmic staining (Figure 2A; Supplementary Figure 1 and 2). Occasionally, nuclear PSA (Supplementary Figure 1C; Supplementary Figure 2B) and secreted PSA in the lumen of the prostatic glands (Supplementary Figure 2C) were observed.

Next, we analyzed AR and PSA protein expression in 23 CRPC samples including 20 samples (CRPC1–20) in a tissue microarray (TMA) and 3 regular CRPC (CRPC21–23) samples (Figure 2C; Supplementary Figure 3A–3B). AR expression showed wide variability in these CRPC samples. For example, CRPC5 and CRPC12 showed apparently increased AR expression and AR^+^ PCa cells compared to untreated PCa but many CRPC samples (e.g., CRPC9, 16, and 20–23) significantly lacked AR^+^ PCa cells (Figure 2C; Supplementary Figure 3, Ab, B). Furthermore, in all AR^+^ CRPC samples, AR^−^ PCa cells could be readily identified, e.g., in CRPC8 (Figure 2C) and CRPC2 and 7 (Supplementary Figure 3B). In sharp contrast to the AR expression patterns, the majority of the 23 CRPC samples mostly lacked appreciable PSA expression or PSA^+^ PCa cells (Figure 2C; Supplementary Figure 3A–3B). Only one sample (CRPC12) was found to have somewhat concordant AR and PSA expression and only CRPC19 (the patient was treated with Lupron for ∼2 weeks) expressed high intratumoral PSA (Supplementary Figure 3A). The IHC studies in this cohort of 23 CRPC samples indicate that *PSA^−/lo^* PCa cells (which can be AR^+^ or AR^−^) are enriched in patient CRPC samples.

Subsequently, we investigated the relative abundance of the 4 subtypes of PCa cells in 3 AD (androgen-dependent) and AI (androgen-independent) PCa xenograft models, LNCaP, LAPC4 and LAPC9 (13). In all 3 models, the AI tumors were highly enriched in PSA^−/lo^ PCa cells (Figure 2D–2E; Supplementary Figure 3C–3D; data not shown). In LNCaP AD tumors, ∼80% of the cells were AR^+^PSA^+^ and the other 3 subtypes of cells represented the minority (Figure 2D–2E; Supplementary Figure 3C). In contrast, the LNCaP AI tumors showed greatly reduced AR^+^PSA^+^ cells and dramatically increased PSA^−/lo^ (AR^+^PSA^−/lo^ and AR^−^PSA^−/lo^) cells (Figure 2D–2E; Supplementary Figure 3C). Similarly, PSA^−/lo^ PCa cells were significantly increased in LAPC4 (Supplementary Figure 3D) and LAPC9 (not shown) AI tumors. Interestingly, in LAPC4 AI tumors, most AR localized to the cytoplasm (Supplementary Figure 3D).

To explore potential differences between subtypes of PCa cells in response to therapeutics, we performed a preliminary study in three types of LNCaP cells (Figure 2F), i.e., AR^+^PSA^+^ wild-type LNCaP, AR^+^PSA^−^ LNCaP-abl [25], and AR^−^PSA^−^ LNCaP-CDSS and LNCaP-MDV cells, the two castration-resistant LNCaP sublines we recently established (Rycaj et al., manuscript submitted). We treated these 3 LNCaP cell types with two antiandrogens, i.e., bicalutamide and MDV3100 (MDV; Enzalutamide), two chemotherapeutic drugs (etoposide and docetaxel), and two molecularly targeted drugs, i.e., ABT-199, which selectively inhibits Bcl-2 [26, 27], and AEW541, an inhibitor of IGF-1R [28], which is important for the PSA^−/lo^ PCa cells [13]. In this relatively short (72 h) cytotoxicity assay, the three LNCaP cells manifested differential responses to the 6 drugs (Figure 2G). The AR^+^PSA^+^ wild-type LNCaP cells displayed responses to all 6 drugs except Bicalutamide whereas AR^+^PSA^−^ LNCaP-abl cells behaved overall similarly to wild-type LNCaP cells and showed only resistance to 10 μM ABT-199 (Figure 2G). In contrast, the AR^−^PSA^−^ LNCaP-CDSS and LNCaP-MDV cells manifested prominent resistance to both etoposide and docetaxel as well as to MDV and ABT-199 (Figure 2G). Interestingly, LNCaP-abl cells showed higher sensitivity to 10 μM AEW541 than both wild-type LNCaP and LNCaP-CDSS and LNCaP-MDV cells (Figure 2G). This pilot experiment establishes the proof-of-principle that subtypes of PCa cells with distinct AR and PSA expression profiles may respond differently to anticancer therapeutics.

### PSA^−/lo^ PCa cells: Heterogeneity in AR expression, quiescence, and resistance to antiandrogens and other therapeutics

The converging findings from the above studies are that: 1) the PSA^−/lo^ PCa cells pre-exist in untreated HPCa; 2) PSA^−/lo^ PCa cells become enriched in patient CRPC and AI xenograft models; and 3) PSA^−/lo^ PCa cells respond to antiandrogens and several other therapeutics differently than the PSA^+^ PCa cells. We recently employed a series of lentiviral GFP/RFP reporters to separate PSA^−/lo^ from PSA^+^ PCa cells to compare their molecular, cell biological, and tumorigenic properties [13]. Herein, we continue to use this system to further explore the cellular and molecular distinctions between these cell subsets, investigate their differential responses to therapeutics *in vitro* and to systemic androgen levels *in vivo*, and interrogate the relationship between the PSA^−/lo^ PCa cells vs. several other PCSC populations.

Infection of LNCaP cells with the PSAP-GFP lentivector at an MOI of 20 led to 100% infection and GFP positivity faithfully reported the endogenous PSA expression [13]. Consistent with earlier results [13], all PSA^+^ (i.e., GFP^+^) LNCaP cells were nuclear AR^+^ whereas only ∼30% PSA^−/lo^ (i.e., GFP^−/lo^) LNCaP cells had strong nuclear AR (Supplementary Figure 4A). Similar results were obtained in LAPC9 and LAPC4 xenografts [13; data not shown]. These observations suggest that the PSA^−/lo^ PCa cell population is heterogeneous with respect to AR expression, consistent with the above IHC-based immunophenotypic analysis of AR and PSA expression in both untreated HPCa and CRPC samples.

**Figure 3:**
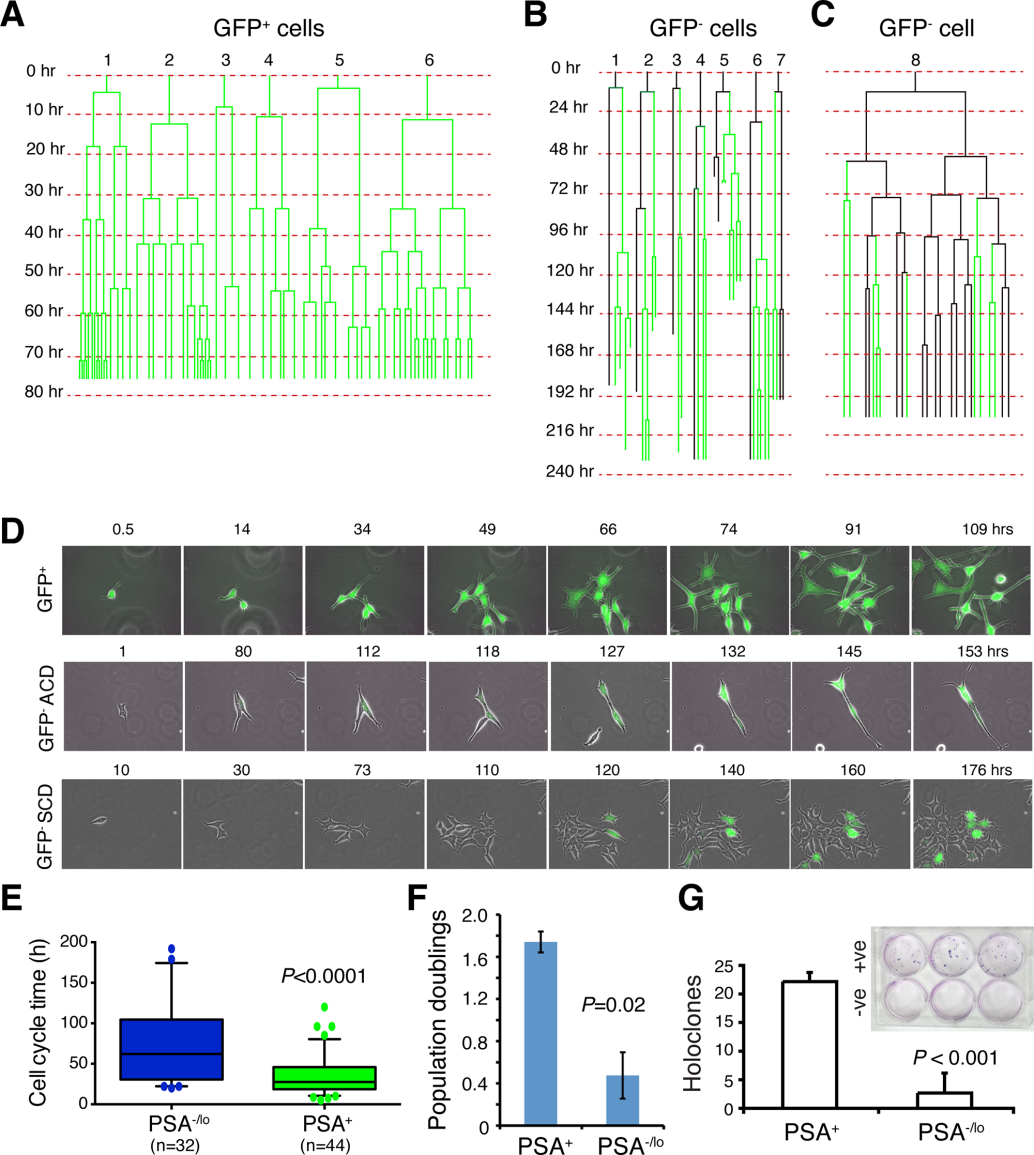
PSA^−/lo^ LNCaP cells are more quiescent than PSA^+^ cells **A–C.** Cell division mode and cell-cycle transit times in LNCaP cells in regular serum-containing culture medium as determined by time-lapse videomicroscopy. Shown in A are six representative GFP^+^ LNCaP cells that underwent symmetrical cell divisions. Shown in B are seven representative GFP^−^ LNCaP cells that underwent asymmetric cell divisions. Shown in C is one GFP^−^ LNCaP cell that underwent complex cell divisions (see Text). Time scale is shown on the left for each cell recorded. **D.** Time-lapse images showing one GFP^+^ LNCaP cell undergoing symmetrical cell divisions in the first round and all subsequent rounds (top panels), one GFP^−^ cell undergoing ACD during the first cell division (middle panels), and one GFP^−^ cell undergoing symmetrical cell division during the first cell division followed by complex division modes in the progeny (bottom panels). **E.** Graphical presentation of cell-cycle transition times in PSA^−/lo^ vs. PSA^+^ LNCaP cells based on the time-lapse tracking of the 2 cell types. **F.** PSA^+^ and PSA^−/lo^ LNCaP cells were FACS-purified and plated in quadruplicate in 96-well plate (1,500 cells/well) and cultured in regular serum-containing medium. Live cells were enumerated 3 days after plating and presented are the population doublings. **G.** PSA^+^ (+ve) and PSA^−/lo^ (−ve) LNCaP cells were plated at clonal density (100 cells/well in triplicate) and cultured in RPMI-5% FBS plus 10 nM R1881 for 2 weeks. At the end, holoclones were enumerated. Shown are the bar graphs (mean ± S.D) pooled from three repeat experiments and a representative Giemsa-stained image (inset).

We have previously demonstrated [13] that under time-lapse videomicroscopy, single PSA^+^ PCa cells exclusively undergo symmetrical cell divisions (SCD) whereas PSA^−/lo^ PCa cells undergo both SCD and asymmetrical cell division (ACD). Here we employed time lapse-based single-cell tracking to determine cell-cycle transit times in two populations of LNCaP cells (Figure 3A–3E). As observed previously [13], the PSA^+^ (i.e., GFP^+^) LNCaP cells underwent rapid and exclusive SCD to generate more PSA^+^ cells (Figure 3A; Figure 3D, top). In contrast, many PSA^−/lo^ (i.e., GFP^−^) LNCaP cells underwent ACD during the first cell division (Figure 3B; Figure 3D, middle). Very occasionally, we observed rare PSA^−/lo^ cells that underwent SCD during the first cell division followed by complex division modes during subsequent divisions (Figure 3C; Figure 3D, bottom). Strikingly, the PSA^+^ daughter cells derived from ACD in most cases underwent rapid SCD whereas the PSA^−/lo^ mother cells rarely divided again (Figure 3B), suggesting that the PSA^−/lo^ cells overall divided more slowly than the isogenic PSA^+^ cells. Indeed, quantification of time-lapse images indicated that the PSA^−/lo^ LNCaP cells had longer average cell-cycle transit times than PSA^+^ cells (Figure 3E). Consistent with the single cell analysis, PSA^−/lo^ LNCaP cells demonstrated lower cumulative population doublings (Figure 3F) and holoclone [10] forming efficiency (Figure 3G) in regular medium containing serum (which contained small amount of steroid hormones) than the corresponding PSA^+^ cells. In another holoclone assay, in which we sorted single PSA^+^ and PSA^−/lo^ LNCaP cells into 96-well plates and cultured them in serum-containing medium. 18 days later, 19 holoclones developed in 36 single PSA^+^ LNCaP cells (i.e., cloning efficiency = 53%) whereas 24 clones developed in 83 single PSA^−/lo^ cells (cloning efficiency = 29%). Taken together, these results suggest that the PSA^−/lo^ PCa cells, in the presence of androgen, are more quiescent than PSA^+^ PCa cells.

**Figure 4:**
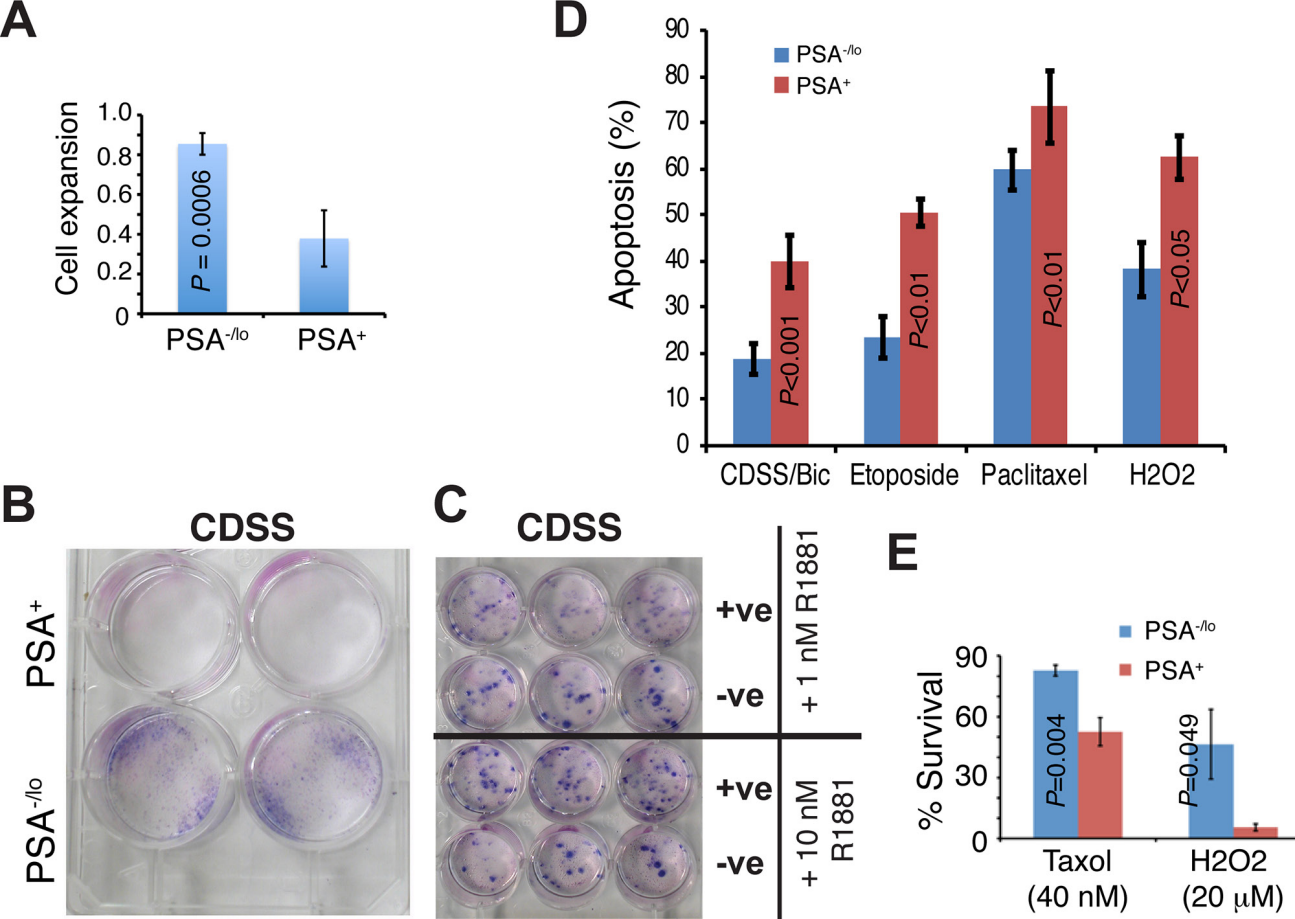
Differential apoptotic responses of PSA^−/lo^ and PSA^+^ LNCaP cells to therapeutics **A.** PSA^+^ and PSA^−/lo^ LNCaP cells were plated (1,500/well) in quadruplicate in RPMI containing either 7% regular FBS or 7% charcoal dextran stripped serum (CDSS). 11 days later, live cells were measured by MTT assays. The results are presented as the cell growth (expansion) of each population in CDSS medium RELATIVE to the corresponding FBS medium (which is 1). **B.** PSA^+^ and PSA^−/lo^ LNCaP cells (10,000/well) were cultured in RPMI containing 7% CDSS for 1 month and plates were stained by Giemsa. **C.** PSA^+^ and PSA^−/lo^ LNCaP cells (10,000/well) were cultured in RPMI-7% CDSS plus either 1 nM or 10 nM R1881 for 25 days and plates were stained by Giemsa. Note that R1881 dose-dependently ‘overcame’ the CDSS effect and promoted the clonal expansion of PSA^+^ LNCaP cells. **D.** Apoptosis assessed by the Vybrant apoptosis assays. Unsorted bulk LNCaP cells infected with PSAP-GFP lentiviral reporter were plated at 120 k cells/well in 6-well plates. Cells were treated for 4 days with either DMSO, 2% CDSS plus 20 μM Bicalutamide (CDSS/Bic), 20 nM Paclitaxel, 1 μM etoposide, or 1 μM H2O2. The % apopotsis represents the mean ± S.D (*n* = 3) and *P* values determined by Student’s *t*-test. No difference in apoptosis was observed in the two populations in response to vehicle DMSO (not shown). **E.** PSA^−/lo^ LNCaP cells preferentially survive stress treatments. Purified PSA^−/lo^ and PSA^+^ cells were plated (1,000/well) in 96-well plate in regular serum-containing medium containing Taxol (Docetaxel) or H2O2 for 48 h. At the end of treatments, live cells were measured by MTT assays and cell survival normalized to vehicle control DMSO (which is 100%).

Are there any differences between PSA^−/lo^ and PSA^+^ PCa cells in the absence of androgen or in the presence of stresses? In our earlier studies [13], we performed cDNA microarray analyses comparing gene expression profiles in PSA^−/lo^ and PSA^+^ LNCaP as well as xenograft LAPC9 cells. A total of 570 probesets representing 337 genes (see Methods) were commonly upregulated (1.5 fold; *P* < 0.05) in PSA^−/lo^ cells in both cell types (Supplementary Figure 4B). Remarkably, when we performed Gene Ontology (GO) analysis on the 337 genes using DAVID, the top 10 GO terms were all related, in some ways, to cellular responses to stress and wound healing (Supplementary Figure 4C). Preferential enrichment of anti-stress and regeneration genes coupled with their quiescent nature would render the PSA^−/lo^ PCa cells resistant to stresses and therapeutics. Several experiments confirmed this prediction. First, when acutely purified PSA^+^ and PSA^−/lo^ LNCaP cells were cultured in androgen-deficient conditions, i.e., in medium containing charcoal dextran-stripped serum (CDSS), the PSA^−/lo^ cells underwent significant expansion (Figure 4A), sharply contrasting with the scarce growth observed in androgen-proficient conditions (Figure 3F). As a matter of fact, only the PSA^−/lo^ LNCaP cells showed significant survival and expansion during continued culture of up to 1 month (Figure 4B). Importantly, the suppressive effects of CDSS on PSA^+^ LNCaP cells could be dose-dependently relieved by exogenous R1881 (Figure 4C). In another set of experiments, we treated the two purified populations of LNCaP cells side-by-side with CDSS plus bicalutamide (20 μM), etoposide (1 μM), paclitaxel (20 nM), or H2O2 (1 μM) for 4 days and then analyzed for apoptosis. As shown in Figure 4D, the PSA^−/lo^ LNCaP cells were more resistant to all these treatments. Finally, we performed yet another set of side-by-side experiments with the two purified populations using the MTT assays to measure the cells that survived treatments. As shown in Figure 4E, PSA^−/lo^ cells survived better than PSA^+^ LNCaP cells in response to both Taxol and H2O2. Since we employed two purified populations of LNCaP cells to directly compare their apoptotic sensitivities (Figure 4), the results excluded the possibility that treatments caused de-differentiation in turning PSA^+^ LNCaP cells to PSA^−/lo^ cells during the treatment period (i.e., 4 days). In support, we observed that all live PSA^+^ LNCaP cells 48 h after treatments remained GFP^+^ (not shown).

### Systemic androgen levels regulate the relative abundance of PSA^+^ and PSA^−/lo^ PCa cells in tumors

**Figure 5:**
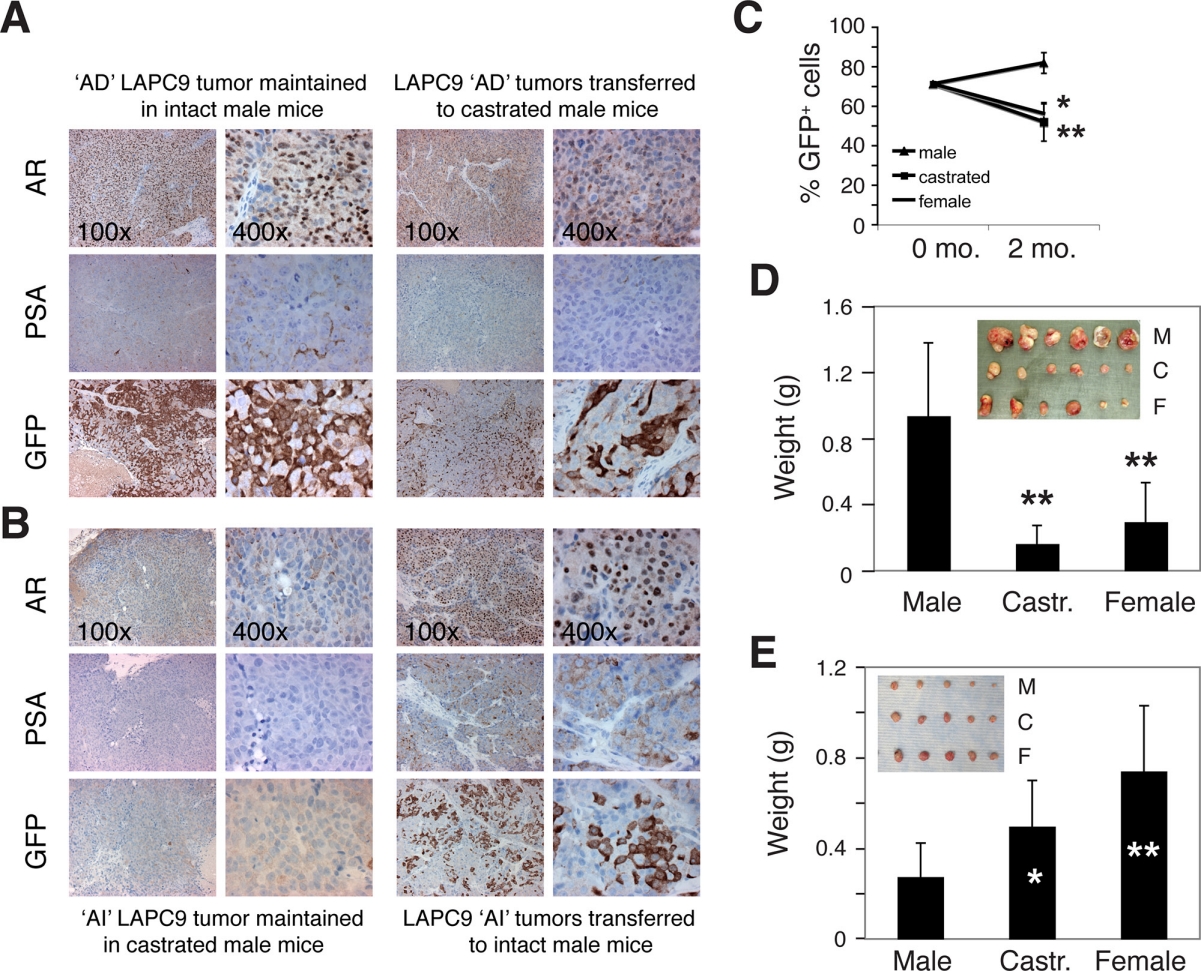
Systemic androgen levels regulate the relative abundance of PSA^+^ and PSA^−/lo^ cells in the tumors **A–B.** Systemic androgen regulates the abundance of PSA^+^ PCa cells in LAPC9 tumors. (A) The majority of PCa cells in LAPC9 reporter tumors maintained in intact male NOD-SCID mice expressed nuclear AR, PSA, and GFP (the left panel; note less sensitive PSA staining than corresponding GFP staining). When LAPC9 tumors in male mice were transferred to castrated mice, AR became excluded from nucleus (which was expected due to lack of the ligand), PSA staining was reduced, and % GFP^+^ cells significantly decreased (right panels). (B) Tumor cells in the LAPC9 reporter tumors maintained in castrated male mice showed dramatically reduced numbers of AR^+^ and PSA^+^, and GFP^+^ cells (the left panel; note that in these tumors GFP sequence could be readily detected by PCR analysis of genomic DNA; not shown); however, when the LAPC9 tumors in castrated mice were transferred back to intact male mice, many tumor cells again displayed nuclear AR as well as PSA/GFP positivity (right panels). **C.** LAPC9 tumor cells were purified from a maintenance reporter tumor maintained in intact male NOD/SCID mice). The bulk tumor cells contained ∼72% GFP^+^ LAPC9 cells as assessed by FACS (i.e., at 0 month). Then 100,000 unsorted LAPC9 cells were injected subcutaneously, in 50% Matrigel, in intact male mice, castrated male mice (castrated ∼2 weeks earlier), or female mice (*n* = 4 for each), respectively. Two months after tumor cell implantation, tumors were harvested and the % of GFP^+^ cells in each tumor was determined by FACS. *P < 0.05 and **P < 0.01, when compared to the tumors in male mice. **D–E.** Bulk LAPC9 cells purified from maintenance tumors in male (D) or castrated (E) mice were injected (200, 000 cells/injection) s.c in three different types of hosts (M, male; C, castrated; F, female). Tumor weights (mean ± S.D) were presented. *P < 0.05; **P < 0.01. Insets: tumor images.

We next explored how systemic androgen levels dynamically affect the relative abundance of PSA^−/lo^ vs. PSA^+^ cells in the tumors (Figure 5). LAPC9 tumors continuously maintained in male mice (i.e., the ‘AD’ tumors) contained 20.9% ± 10.3% (*n* = 10) PSA^−/lo^ cells with the majority being PSA^+^ cells (Figure 5A, and 5C). When bulk LAPC9 cells from these AD tumors were transferred to androgen-deficient hosts (i.e., either castrate male or female mice) for ∼2 months, PSA^+^ cells declined significantly whereas PSA^−/lo^ cells increased to ∼50% (Figure 5A, and 5C). When LAPC9 tumors were maintained in androgen-deficient hosts for ∼2 years (i.e., the ‘AI’ tumors), PSA^−/lo^ cells increased to 89.3% ± 9.8% (*n* = 12) (Figure 5B). When unsorted LAPC9 cells from such AI tumors were put back in intact male mice, PSA^+^ LAPC9 cells in the tumors again increased (Figure 5B). These results are remarkably similar to what we observed earlier in AD/AI LNCaP and LAPC4 systems and suggest that systemic androgen levels dynamically regulate the abundance of PSA^+^ vs. PSA^−/lo^ cells in prostate tumors.

When unsorted LAPC9 cells from the AD tumors, in which 70–90% cells were PSA^+^, were implanted in different hosts, they initiated much larger tumors in male mice than in castrated male or female mice (Figure 5D). In contrast, when bulk LAPC9 cells from the AI tumors, in which ∼90% cells were PSA^−/lo^, were implanted in different hosts, they initiated larger tumors in androgen-deficient hosts (Figure 5E). These results indicate that the relative abundance of PSA^+^ versus PSA^−/lo^ cells greatly influences tumor growth rate in hosts with different levels of androgen.

### Evidence that PSA^−/lo^ PCa cells possess distinct epigenetic profiles: Analysis of bivalent chromatin domains in several genes

**Figure 6:**
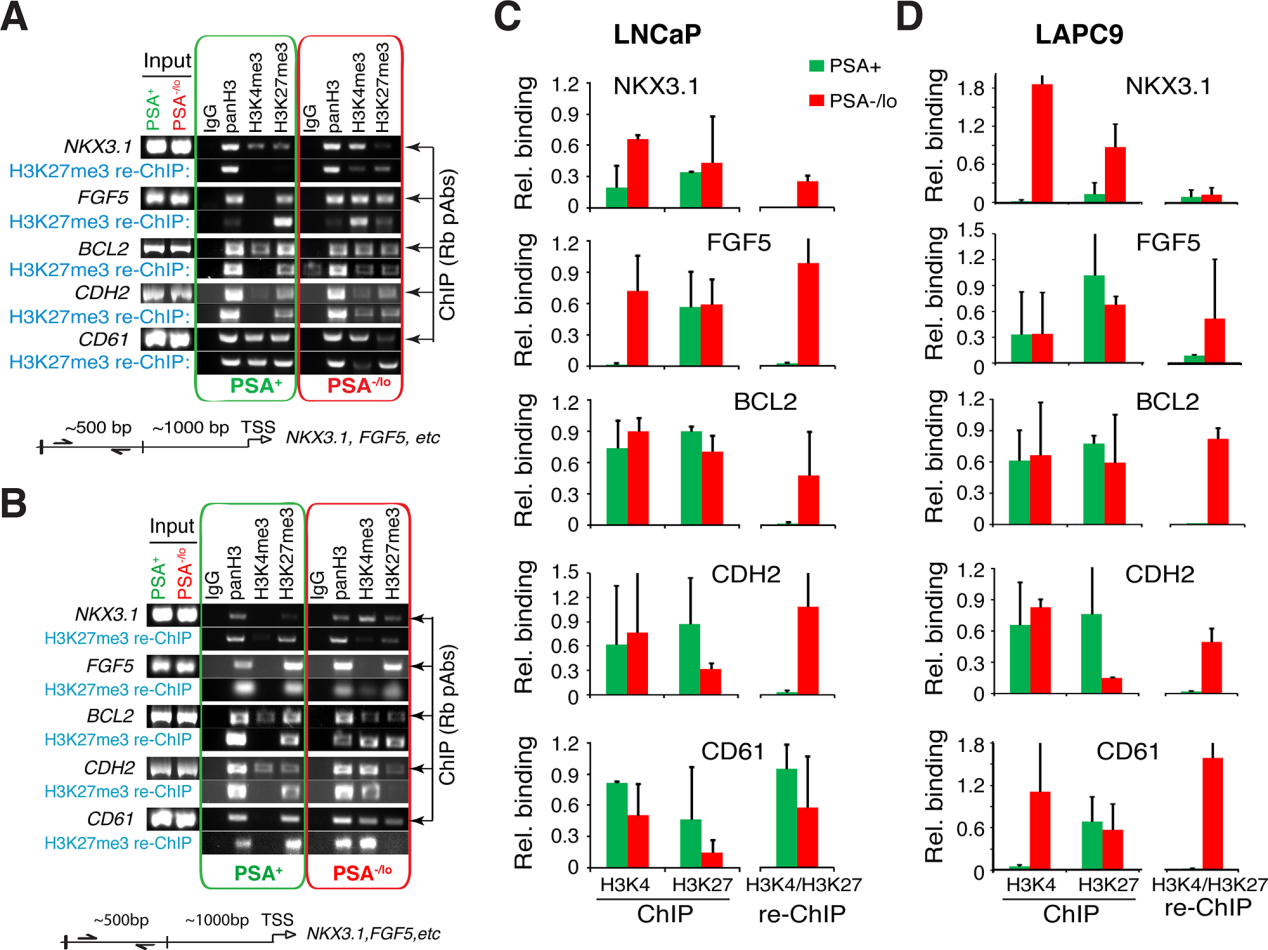
PSA^−/lo^ PCa cells show preferential gene promoter association with bivalent chromatin marks ChIP/re-ChIP experiments were performed in purified PSA^−/lo^ and PSA^+^ LNCaP **(A** and **C).** and LAPC9 **(B** and **D).** cells. ChIP was performed with individual rabbit polyclonal antibodies (Rb pAbs) and re-ChIP was performed with a monoclonal anti-H3K27me3 antibody. Shown are representative gel images (A and B) and quantification (C and D; *n* = 3) normalized to panH3. The re-ChIP bar graphs (C and D; right) represent bivalent marks.

The above observations that systemic androgen levels regulate the relative abundance of the two populations of PCa cells *in vivo* implicate epigenetic mechanisms. Previous microarray analyses showed that the PSA^−/lo^ PCa (LAPC9, LNCaP, as well as HPCa) cells overexpressed several dozens of stem cell-associated genes [13]. Of importance, the PSA^−/lo^ LNCaP cells, compared to PSA^+^ cells, also overexpressed some (e.g., EED, HDAC4, PHF8) whereas under-expressed other (e.g., DNMT3B, PHF19) chromatin modifiers/epigenetic regulators [13]. Embryonic stem cells (ESCs) are enriched in genes associated with bivalent chromatin marks consisting of large regions of the repressive H3 lysine 27 trimethylation (H3K27me3) harboring smaller regions of H3 lysine 4 trimethylation (H3K4me3) [29]. To explore whether PSA^−/lo^ PCa cells may also be epigenetically different from the differentiated isogenic PSA^+^ cells, we performed ChIP and re-ChIP (also called ChIP and sequential ChIP) analysis using the Bernstein protocol [30]. We purified PSA^−/lo^ and PSA^+^ LNCaP and LAPC9 cells and analyzed 8 genes whose promoters have been associated with the bivalent marks in ESCs [29] including FGF5, NKX3.1, BCL2, CDH2 (i.e., N-cadherin), CD61 (i.e., integrin β3), AR, ASCL1, and PPP2R4. We first performed regular ChIP assays using rabbit polyclonal antibodies to pan-histone 3 (panH3), H3K4me3, or H3K27me3 in purified PSA^+^/PSA^−/lo^ LNCaP (Figure 6A) or LAPC9 (Figure 6B) cells. We then performed sequential ChIP on the first ChIP products using a mAb to H3K27me3.

The results revealed that in LNCaP cells, 4 genes, i.e., *NKX3.1*, *FGF5, BCL2*, and *CDH2* showed bivalent binding patterns preferentially in the PSA^−/lo^ cell population (Figure 6C). In contrast, the other 4 genes (i.e., CD61, ASCL1, AR, and PPP2R4) showed overall similar re-ChIP profiles, which did not differ significantly between PSA^−/lo^ vs. PSA^+^ cells (Figure 6C; data not shown). In LAPC9 cells, 4 genes, i.e., *FGF5*, *BCL2*, *CDH2* and *CD61* showed bivalent binding patterns preferentially in PSA^−/lo^ cells (Figure 6D) whereas *NKX3.1* showed similarly low levels of bivalency in both populations. The other 3 genes (ASCL1, AR, and PPP2R4) did not show significant differences in bivalent patterns between PSA^−/lo^ vs. PSA^+^ LAPC9 cells (data not shown). It is interesting that LNCaP and LAPC9 cells showed similar bivalent chromatin marks on 3 gene promoters (i.e., FGF5, BCL2, and CDH2) but differed in NKX3.1 and CD61. Also of interest, LNCaP cDNA microarray analysis revealed higher levels of *NKX3.1* and *FGF5* mRNAs in PSA^−/lo^ cells [13] and correspondingly, our ChIP assays showed high H3K4me3 association with the *NKX3.1* and *FGF5* gene promoters also in PSA^−/lo^ cells (Figure 6C), supporting the preferential activation of these two genes in PSA^−/lo^ LNCaP cells. These preliminary ChIP/re-ChIP results provide evidence that the PSA^−/lo^ and PSA^+^ PCa cells may possess different epigenetic profiles.

### Relationship between PSA^−/lo^ PCa cells and other tumorigenic PCa cell subsets

**Figure 7:**
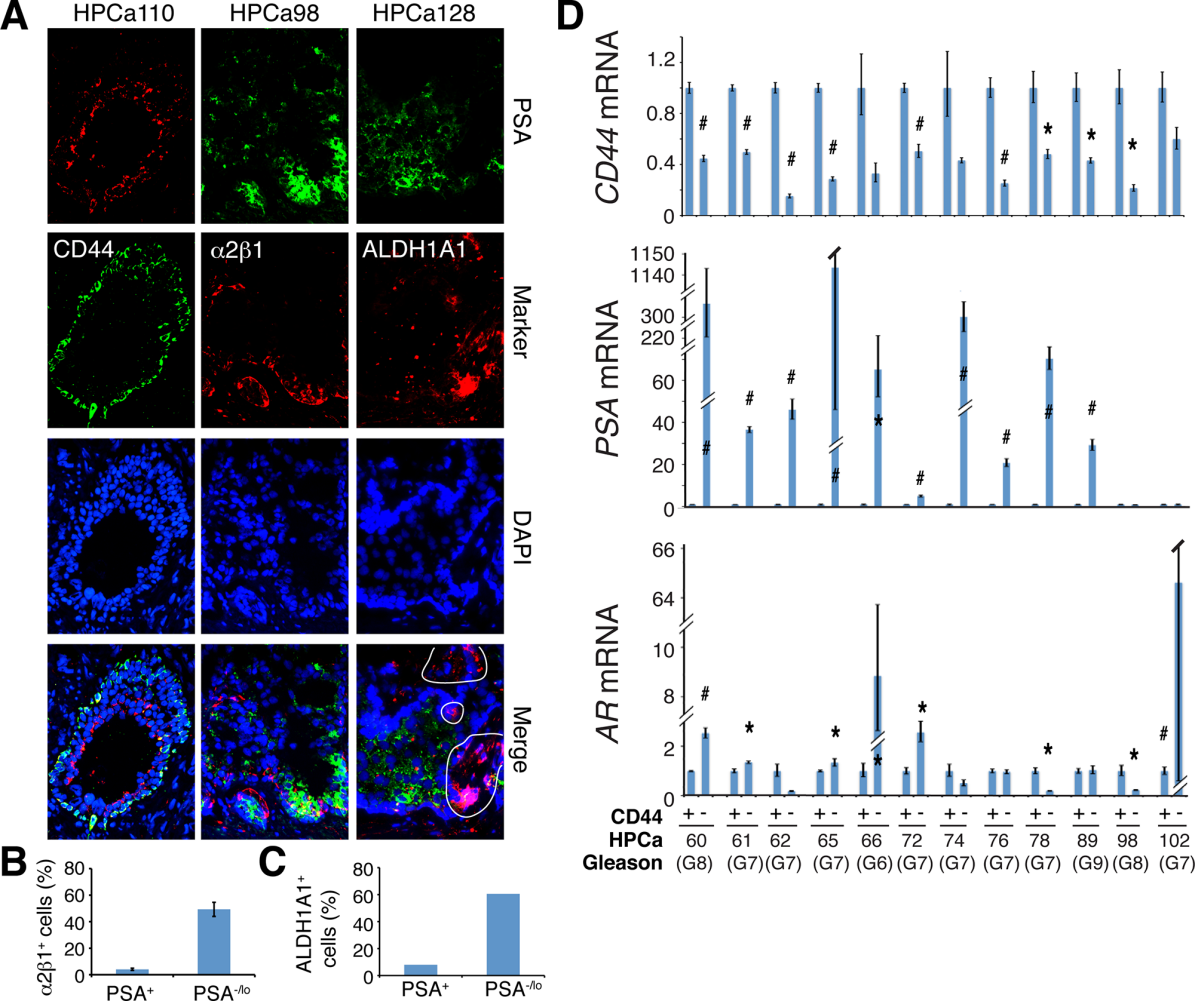
Relationship of PSA^−/lo^ PCa cells and other PCSC marker-expressing subpopulations in HPCa **A.** Representative IF images (×400) illustrating reciprocal expression patterns of ALDH1A1, α2β1, and CD44 versus PSA in the 3 HPCa samples (indicated on top). Note the mutually exclusive staining patterns of PSA versus ALDH1A1 (right; circled areas were ALDH1A1^+^ but PSA^−^), α2β1 (middle), or CD44 (left). **B–C.** Quantification of marker-positive cells in PSA^−/lo^ vs. PSA^+^ HPCa cells. The results for α2β1 were data pooled from counting > 500 cells each in HPCa96, HPCa98, and HPCa110 (B). The PSA^−/lo^ cells contained significantly more α2β1^+^ cells (mean ± S.D; *P* < 0.0001). The bar graph for ALDH1A1 was obtained from counting ALDH1A1-postive cells in ∼1,000 each of PSA^+^ and PSA^−/lo^ cells in HPCa128. **D.** qPCR analysis of *CD44*, *AR*, and *PSA* mRNAs in CD44^+^ and CD44^−^ HPCa cells freshly purified from untreated primary prostate tumors. The results are expressed as relative levels in CD44^+^ HPCa cells to those in the matched CD44^−^ HPCa cells. *P < 0.05; #*P* < 0.01.

In our previous studies, cDNA microarray analysis revealed that the PSA^−/lo^ LAPC9 cells expressed higher mRNA levels of several CSC markers including *CD44*, integrin *α2β1*, and *ALDH1A1* in comparison to PSA^+^ LAPC9 cells [13]. Indeed, using PSA^+^/PSA^−/lo^ LAPC9 cells freshly purified from xenograft reporter tumors [13], we observed lower levels of *PSA* and *AR* mRNAs (Supplementary Figure 5A) but higher levels of *CD44* mRNA (Supplementary Figure 5B) in PSA^−/lo^ cells. Tumors initially derived from PSA^−/lo^ LAPC9 cells, even after 3 passages in intact male mice, still expressed high levels of α2β1, CD44, and ALDH1A1 proteins compared to similarly passaged tumors initially derived from the PSA^+^ cells (Supplementary Figure 5C). These results suggest an opposite relationship between PSA expression and the three phenotypic PCSC markers. Indeed, double IF staining in benign prostate tissues showed basal expression of CD44, ALDH1A1, and α2β1 but luminal expression of PSA (Supplementary Figure 5D–5E). Similar experiments in HPCa samples also revealed mutually exclusive expression patterns between PSA versus the three PCSC markers (Figure 7A). Differential quantification demonstrated that α2β1^+^ (Figure 7B) and ALDH1A1^+^ (Figure 7C) cells were mainly PSA^−/lo^. Strikingly, when we performed the opposite experiments by purifying out CD44^+^/CD44^−^ primary tumor cells from 12 untreated tumor samples (Supplementary Table 2) and analyzing *AR* and *PSA* mRNAs in the two populations, we found that the *PSA* mRNA was preferentially expressed in CD44^−^ HPCa cells in 10 samples whereas *AR* mRNA expression pattern was more complex with preferential enrichment in CD44^−^ HPCa cells in only 6 samples (Figure 7D). In 4 samples, *AR* mRNA was actually higher in CD44^+^ HPCa cells (Figure 7D).

### Comprehensive dissection of tumorigenic subsets in PCa culture and xenograft models

The above studies in primary human PCa (HPCa) samples (Figure 7; Supplementary Figure 5) suggest a concordant relationship between PSA^−/lo^ cells and PCa cells expressing CSC markers CD44, α2β1, and ALDH1A1. Unfortunately, primary HPCa cells, and even primary HPCa pieces, are well-known to be very difficult to regenerate tumors in immunocompromised mice [11, 31]. Therefore, to further dissect the PCa cell heterogeneity, in this part of the project, we employed both surface markers (CD44, α2β1, and, for comparison, ABCG2) and functional (i.e., SP and Aldefluor) assays to dissect the tumorigenicity of PCa cell subpopulations in three PSA^−^ (Du145, PPC-1 and PC3; all three models do not express AR and PSA and contain only PSA^−^ cells) and three PSA^+^ (LNCaP, LAPC4 and LAPC9; all 3 models contain PSA^+^ and PSA^−/lo^ cells) PCa models. It should be noted that although we have previously reported tumor-initiating cells in some of these models [6–12], those studies were conducted in by different investigators and at different time points. Herein, we aim to conduct side-by-side, in-depth dissection of PCa cell heterogeneity in the same models. We performed a spectrum of functional assays *in vitro* and (serial) tumor transplantations by implanting 1 to 5 × 10^5^ cells in NOD/SCID mice followed by determining and comparing the tumor-initiating frequency (TIF) of matched PCa cell subpopulations. As we describe below, the results revealed distinct phenotypic profiles of tumor-initiating cells in individual PCa models.

*In vitro* studies in the 4 PCa cell lines (LNCaP, Du145, PPC-1 and PC3) showed (Supplementary Figure 6; Supplementary Table 3) that they all expressed the luminal cell marker cytokeratin 18 (CK18) but only LNCaP cells expressed the differentiation markers AR and PSA. In contrast, the basal/stem cell markers CD44, α2β1, and CK5 were not detected in LNCaP cells but observed in a fraction of Du145 cells and expressed in the majority of PC3 and PPC-1 cells. We also measured telomerase activity in these cells, which mirrored the expression pattern of basal/stem cell markers (Supplementary Table 3). Interestingly, the clonogenic, tumorigenic, and metastatic capacity of the 4 PCa cells positively correlated with their telomerase activity and the abundance of basal/stem cell markers.

**Figure 8:**
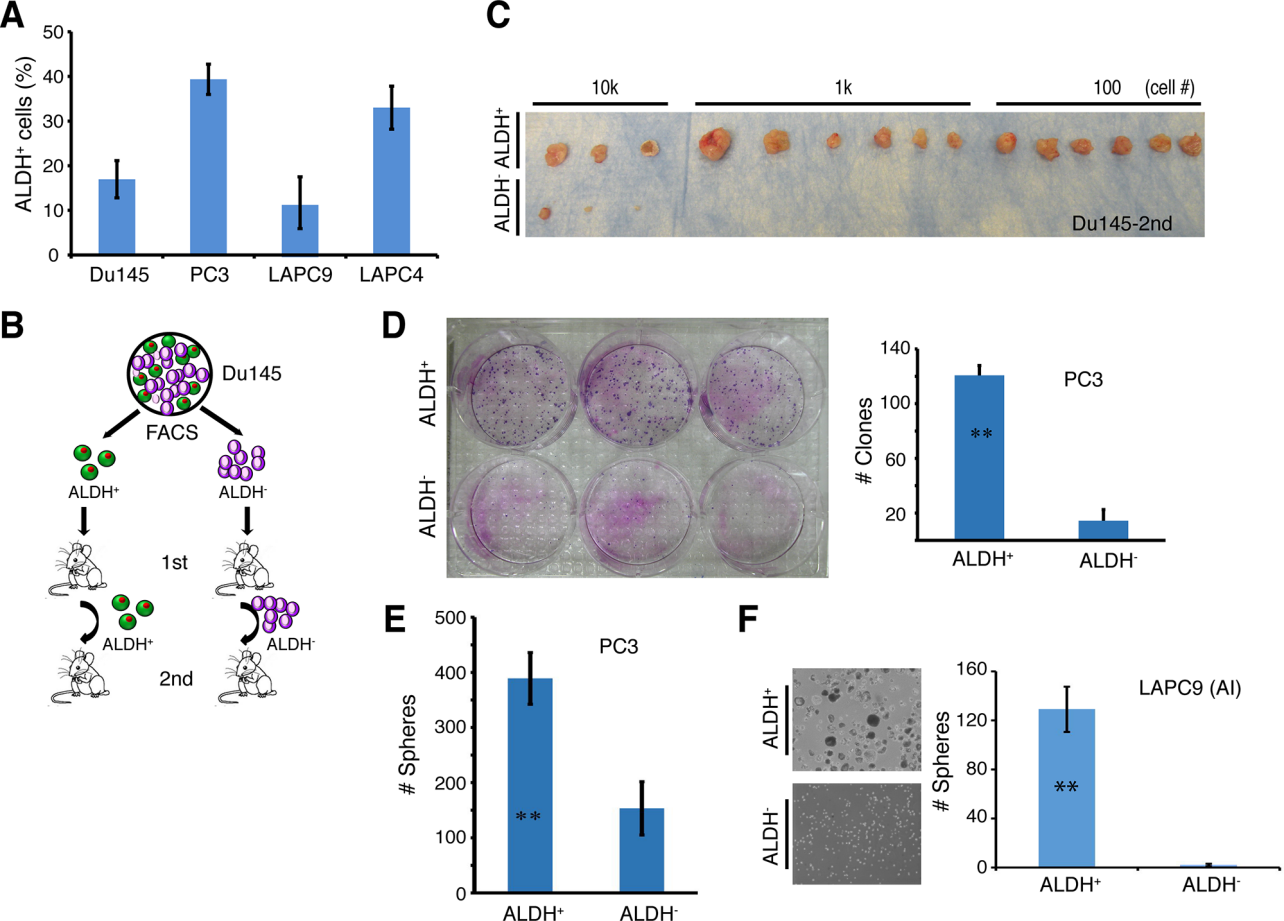
The ALDH^+^ PCa cell subpopulations are enriched in self-renewing tumor-initiating cells **A.** The percentage of ALDH^+^ cells in four PCa models. ALDH activity was measured by the ALDEFLUOR assay and analyzed by flow cytometry. Tumor cells purified from Du145 and PC3 cultures or LAPC9 and LAPC4 xenografts were incubated in ALDEFLUOR assay buffer containing ALDH substrate and analyzed by FACS. Cells treated with DEAB were used as negative control. Shown is the bar graph derived from at least 3 independent experiments (mean ± SEM). **B.** Experimental scheme for Du145 serial tumor transplantation assays. ALDH^+^ and ALDH^−^ Du145 cells were purified and used for LDA in intact male NOD/SCID mice. The 1° tumors derived from ALDH^+^ and ALDH^−^ were harvested and utilized to purify ALDH^+^ and ALDH^−^ cells, respectively, for 2° transplantation. **C.** ALDH^+^ and ALDH^−^ Du145 cells were sorted from 1° tumors derived from ALDH^−^ and ALDH^+^ cells, respectively, and LDA was performed in NOD/SCID male mice (see also Table 1). Shown were 2° tumor transplantation images at the cell doses indicated. **D.** PC3 cells were sorted by FACS for ALDH^+^ and ALDH^−^ cells, and plated at clonal density (400 cells/well in 6-well dishes) in triplicate. Nine days after plating, holoclones were counted. Shown is the bar graph (mean ± S.D; *n* = 3; **P < 0.001) and clone images. **E.** ALDH^+^ and ALDH^−^ PC3 cells were sorted and cultured in anchorage-independent conditions. 10 days later, spheres were counted. Presented are the mean ± S.D (*n* = 3; **P < 0.01). **F.** ALDH^+^ and ALDH^−^ cells were purified from a LAPC9 xenograft tumor long-term maintained in castrated male mice (AI) and cultured in ultra-low attachment plates. Shown are the representative sphere images (left) and bar graphs (mean ± S.D; *n* = 4, **P < 0.001).

Subsequently, we performed limiting-dilution tumor-regeneration assays (LDA) in Du145 and PC3 cells, two surrogate PSA^−^ PCa models, using both marker-based and functional assays (Table 1; Figure 8A–8E; Supplementary Figure 7–8). Among the 3 single surface marker (ABCG2, CD44, and α2β1) profiles, the ABCG2^+^ Du145 cell population (from either cultures or xenografts) manifested significantly higher tumor-regenerating activity than the ABCG2^−^ population (Table 1). Consistent with our earlier results (7), the CD44^+^ Du145 cells were >30 fold more tumorigenic than the CD44^−^ counterparts (Table 1). The integrin α2β1^+^ Du145 cells were also much more tumorigenic than the α2β1^−^ Du145 cells (Table 1; Supplementary Figure 7). Interestingly, when we sorted out Du145 cells double positive for CD44 and α2β1, there was only ∼2 fold difference in TIF between CD44^+^α2β1^+^ vs. CD44^−^α2β1^−^ populations, which was not statistically significant (Table 1; see below). In the two functional (i.e., SP and Aldefluor) assays performed, Du145 cells did not show a detectable SP (not shown), as we previously reported [6]. In contrast, ∼20% Du145 cells had high Aldefluor activity (i.e., ALDH^+^; Figure 8A; Supplementary Figure 8A). The ALDH^+^ Du145 cells demonstrated relatively higher clonogenic capacity (Supplementary Figure 8B) and significantly higher tumorigenicity (Supplementary Figure 8C; Table 1) than ALDH^−^ cells. In secondary (2°) tumor transplantation experiments (Figure 8B), the ALDH^+^ Du145 cells purified from the first generation (1°) tumors were greatly enriched in tumor-regenerating activity giving rise to a striking TIF of 1/1 (Figure 8C; Table 1), suggesting that nearly every single ALDH^+^ cell was tumorigenic. ALDH^+^ Du145 cells self-renewed *in vivo* as both the 1° (Supplementary Figure 8D) and 2° (not shown) tumors, like the parental cultures, harbored only a fraction of ALDH^+^ cells with the majority being ALDH^−^.

**Table 1:** Tumor-initiating frequecy (TIF) of Du145 and PC3 cells

Phenotype*	Cell dose					TIF (range)^$^		P value^#^
	10^5^	10^4^	10^3^	10^2^	10			(fold differ.)
**Du145**								
ABCG2^+^ (cells)			2/6	3/8		1/1,100 (1/415-1/2,915)		6.44e-119 (10x)
ABCG2^−^ (cells)		2/6	1/6	2/6		1/10,897 (1/4060-1/29,246)		
ABCG2^+^ (xenografts)			1/2	2/6		1/623 (1/165-1/2,347)		1.19e-89 (13x)
ABCG2^−^ (xenografts)		1/4	2/6	2/6		1/7,891 (1/2,686-1/23,183)		
CD44^+^			5/8	5/8		1/530 (1/245-1/1,146)		7.6e-210 (33x)
CD44^−^		3/8	0/6	1/8		1/17,584 (1/6,395-1/48,350)		
α2β1^+^	8/8	7/8	3/8			1/3,744 (1/1,694-1/8,275)		8.47e-09 (31x)
α2β1^−^	2/5	2/8	0/8			1/115,913 (1/40,331-1/333,137)		
CD44^+^α2β1^+^		4/8	2/8	1/7	0/8	1/9,152 (1/4,034-1/20,765)		
CD44^+^α2β1^−^		1/8	1/7	0/8	0/8	1/41,048 (1/9,936-1/169,575)		
CD44^−^α2β1^+^		0/3	1/8	0/8	0/8	1/38,298 (1/4,922-1/298,016)	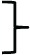	0.0396
CD44^−^α2β1^−^		2/4	0/8	0/8		1/18,963 (1/4,832-1/74,420)		
ALDH^+^			3/4	1/4		1/615 (1/205-1/1,842)		2.62e-77 (64x)
ALDH^−^		1/4	0/4	0/4		1/39,188 (1/5,558-1/276,314)		
ALDH^+^ (2°)		3/3	6/6	6/6		1/1 (1/1-1/108)		1.07e-141 (6,025x)
ALDH^−^ (2°)	3/4 (0.5×104)	0/4	0/4			1/6,025 (1/1,995-1/18,195)		
**PC3**								
ABCG2^+^		5/5	6/8	2/8		1/615 (1/283-1/1,336)		0.253
ABCG2^−^		7/8	8/8	5/8		1/1,071 (1/457-1/2,512)		
ALDH^+^		10/10	4/7	5/8		1/552 (1/245-1/1,245)		0.00869 (4x)
ALDH^−^		10/11	7/12	2/6		1/2,003 (1/944-1/4,250)		

* Marker-positive and -negative Du145 and PC3 cells were sorted out by FACS from log-phase cultures or, in some cases, from xenografts (indicated), and injected subcutaneously in Matrigel (1:1) in female NOD/SCID mice. Tumors were harvested generally 2-4 months after cell implantations except the experiments with CD44α2β1, which were terminated at ∼5 months after implantation.

$ TIF was determined using the L-CalcTM software (http://bioinf.wehi.edu.au/software/elda/index.html). The ranges were indicated in the parentheses.

# The P values between marker-positive and marker-negative populations were determined by Chi-Square (χ^2^) test. Indicated in parenthesis are relative fold enrichment in tumorigencity by comparing TIF in marker-positive and -negative cell populations.

PC3 cells, unlike Du145, were nearly all positive for CD44 and α2β1 (Supplementary Figure 6A; Supplementary Table 3). Therefore, these two surface markers would not be able to stratify tumorigenic vs. non-tumorigenic subsets. On the other hand, ∼40% PC3 cells were ALDH^+^ (Figure 8A; Supplementary Figure 8A) and purified ALDH^+^ PC3 cells showed much higher clonal (Figure 8D), sphere-formation (Figure 8E), and tumor-regeneration (Table 1) capacities than the corresponding ALDH^−^ PC3 cells.

Next, we studied LAPC9 and LAPC4, two xenograft models that contain both AR^+^/AR^−^ and PSA^+^/PSA^−^ cells [13]. Unlike what we observed in Du145 cells, ABCG2^+^ and ABCG2^−^ LAPC9 cells did not show any difference in tumorigenic capacities (Table 2). The α2β1^+^ and α2β1^−^ LAPC9 cells, whether implanted subcutaneously or in the DP, also did not manifest any difference in tumor-regenerating activity (Table 2). CD44^+^ LAPC9 cells, however, when implanted subcutaneously or orthotopically in the dorsal prostate (DP), exhibited ∼6- and 19-fold, respectively, higher tumor-initiating potential than corresponding CD44^−^ LAPC9 cells (Table 2). The higher tumor-initiating capacity of CD44^+^ LAPC9 cells was corroborated in an independent orthotopic LDA experiment (Supplementary Figure 9A). Importantly, the *in vivo* self-renewal ability of the CD44^+^ LAPC9 cells was revealed in 2° transplantation experiments (Supplementary Figure 9B). Remarkably, however, the CD44^+^α2β1^+^ LAPC9 cells, unlike CD44^+^α2β1^+^ Du145 cells, demonstrated > 900 fold enrichment in tumor-initiating capacity compared to the double-negative cells (Table 2). In fact, we even observed tumor development with a single CD44^+^α2β1^+^ LAPC9 cell (Table 2; see discussion below). In the two functional assays we performed, the LAPC9 SP cells, as we observed earlier [6], constituted ∼0.05–1% of the total (not shown) and possessed much higher tumor-initiating capacity than the non-SP cells (Table 2; Supplementary Figure 9C). Like the CD44^+^ and CD44^+^α2β1^+^ cells, the LAPC9 SP cells self-renewed *in vivo* and a single LAPC9 SP cell was able to establish a 2° tumor (Supplementary Figure 9C and 9D). The ALDH^+^ LAPC9 cells in regular AD tumors constituted ∼10% of the total (Figure 8A; Supplementary Figure 8A) and displayed higher sphere-forming (Supplementary Figure 8E) and tumor-regenerating (Table 2) activities than the corresponding ALDH^−^ cells. Interestingly, the ALDH^+^ LAPC9 cells purified from AI tumors, which were enriched in ALDH^+^ cells (not shown), also manifested higher sphere-forming capacity than ALDH^−^ cells (Figure 8F).

**Table 2:** Tumor-initiating frequency of LAPC9 and LAPC4 cells

Phenotype*		Cell dose						TIF (range)^$^	P value^#^
		10^5^	10^4^	10^3^	10^2^	10	1		(fold differ.)
**LAPC9**									
ABCG2^+^				5/8	2/8	1/12		1/719 (1/330-1/1,567)	0.458 (1.5x)
ABCG2^−^			5/5	4/8	2/8			1/1,085 (1/465-1/2,533)	
CD44^+^			4/4	10/11	10/10	1/4		1/137 (1/60-1/311)	0.00124 (6x)
CD44^−^			4/4	5/6	0/8			1/752 (1/308-1/1,839)	
CD44^+^ (DP)			4/9	2/8	0/5			1/12,474 (1/5,350-1/29,082)	1.33e-5 (19x)
CD44^−^ (DP)	4/5 (5×10^5^)	3/5	0/10	0/10	0/5			1/230,530 (1/97,904-1/542,821)	
α2β1^+^			5/6	3/6	0/6		1/3,759	(1/1,528-1/9,244)	0.674 (1.25x)
α2β1^−^		4/6	5/8	6/8	3/6		1/4,694	(1/2,166-1/10,169)	
α2β1^+^ (DP)			1/4	0/4	0/4			1/39,188 (1/5,558-1/276,303)	
α2β1^−^ (DP)		4/4	1/4	0/4	0/4			1/27,813 (1/8,639-1/89,540)	0.766 (1.4x)
CD44^+^α2β1^+^			6/6	6/6	2/2	4/12	1/8	1/21 (1/9-1/49)	
CD44^+^α2β1^−^			8/8	10/10	7/8	2/8		1/44 (1/20-1/96)	0.207
CD44-α2β1^+^			6/8	9/10	4/8	1/8		1/2,040 (1/927-1/4,490)	<0.00001
CD44-α2β1^−^		1/6	2/5	0/8				1/19,791 (1/5,892-1/66,479)	<0.00001(x942)
SP				3/4	2/8			1/554 (1/205-1/1,497)	3.85e-17 (530x)
Non-SP	1/1 (3×105)	0/1	0/4					1/216,403 (1/30,607-1/1,530,060)	
ALDH^+^				7/8	6/8	3/8		1/193(1/79-1/472)	<0.00001 (91x)
ALDH^−^		2/2 (0.5×10^5^)	2/6	1/8	1/8			1/13,607 (1/5,296-1/34,962)	
**LAPC4**									
CD44^+^			3/3	5/6	4/6			1/301 (1/115-1/786)	0.478 (1.4x)
CD44^−^			7/7	13/14	2/14			1/433 (1/240-1/779)	
α2β1^+^ (DP)			0/2	0/4				N/A	0.349
α2β1^−^ (DP)		5/6	1/4	0/4				1/52,266 (1/20,902-1/130,692)	
CD44^+^α2β1^+^				8/8	7/7	1/8		1/35 (1/15-1/80)	0.002 (5.7x)
CD44-α2β1^−^			8/8	7/8	4/8	4/8		1/200 (1/82-1/487)	
ALDH^+^			4/8	7/8	1/8			1/4,897 (1/2,285-1/10,495)	
ALDH^−^			5/8	5/8	5/8			1/3,331 (1/1,503-1/7,373)	0.353

* Marker-positive and -negative LAPC9 or LAPC4 cells were sorted out by FACS from xenograft tumors maintained in intact male NOD/SCID mice and implanted, at different numbers, subcutanesouly (in most cases) or in the dorsal prostate (DP) in Matrigel (1:1) in intact male mice. Tumors were generally harvested in 2-4 months.

$ TIF was determined using the L-CalcTM software (http://bioinf.wehi.edu.au/software/elda/index.html). The ranges were indicated in the parentheses.

# The P values between marker-positive and marker-negative populations were determined by Chi-Square (2) test.

When we purified out CD44^+^/CD44^−^ and α2β1^+^/α2β1^−^ LAPC4 cells from the xenografts and performed similar LDA tumor experiments, surprisingly, the marker-positive and marker-negative subpopulations appeared similarly tumorigenic (Table 2). LAPC4 cells did not have a detectable SP (data not shown) but had ∼35% ALDH^+^ cells (Figure 8A; Supplementary Figure 8A). The ALDH^+^ LAPC4 cells again did not exhibit any difference in tumor-regenerating activity compared to the ALDH^−^ cells (Table 2; Supplementary Figure 8F). If anything, the ALDH^−^ LAPC4 cells appeared to be slightly more tumorigenic than the ALDH^+^ cells (Supplementary Figure 8F). However, CD44^+^α2β1^+^ LAPC4 cells displayed (statistically) higher tumor-regenerating activity than the corresponding CD44^−^α2β1^−^ LAPC4 cells (Table 2; Supplementary Figure 10).

### Further dissection of phenotypic and functional heterogeneity of PCSC subpopulations

The above exhaustive side-by-side tumor studies in two PSA^−^ and two PSA^+^ tumor systems (summarized in Supplementary Table 4) demonstrate that tumor-initiating Du145 cells can be enriched by all three surface markers (ABCG2, α2β1, and CD44) as well as Aldefluor assay but not SP analysis as this model lacks the SP. Tumorigenic LAPC9 cells can be enriched by CD44^+^, CD44^+^α2β1^+^, and SP and ALDH^+^ phenotypes but not the α2β1^+^ or ABCG2^+^ phenotypes. Tumorigenic PC3 cells may be enriched by the ALDH^+^ phenotype but not ABCG2 whereas *only* the CD44^+^α2β1^+^ phenotype can enrich tumor-initiating cells in the LAPC4 model (Supplementary Table 4). Serial tumor transplantation experiments have established that the Du145 ALDH^+^, and LAPC9 CD44^+^, CD44^+^α2β1^+^, and SP populations all can self-renew *in vivo*, attesting to their true CSC properties. These results, collectively, suggest that different PCa models possess distinct profiles of tumorigenic subpopulations.

To investigate the potential relationship between single marker-positive versus double marker-positive PCa cells with respect to their tumor-regenerating activity, we compared CD44^+^α2β1^+^ versus CD44^+^ and α2β1^+^ cells in Du145 and LAPC9 models. Interestingly, the CD44^+^α2β1^+^ Du145 cell population was only slightly enriched in tumor-initiating cells and its tumor-initiating capacity was actually lower than in CD44^+^ Du145 cells (TIF 1/9, 152 vs. TIF 1/530, *P* = 1.27e-07) (Table 1). Also, the CD44^+^α2β1^+^ Du145 cells exhibited only ∼2 fold higher tumorigenic potential than CD44^−^α2β1^−^ cells (1/9, 152 vs. 1/18, 963, *P* = 0.343) (Table 1). In sharp contrast to the Du145 model, the CD44^+^α2β1^+^ LAPC9 cells were highly tumorigenic in that as few as 1 double-positive cell was able to regenerate a tumor (Table 1) and the regenerated tumor contained only a small % of CD44^+^α2β1^+^ LAPC9 cells and could be serially passaged (not shown). Significantly, the CD44^+^α2β1^+^ LAPC9 cell population was more tumorigenic than either CD44^+^ (1/21 vs 1/137; *P* = 0.0014) or α2β1^+^ (1/21 vs. 1/3, 759; *P* = 5.12e14) cell population (Table 1). The contrasting results observed in Du145 and LAPC9 models with respect to the tumorigenicity of CD44^+^α2β1^+^ cells suggest that the ability of combinatorial marker-sorting strategy to further enrich CSCs over single marker strategies is dependent on the cancer model analyzed.

**Figure 9:**
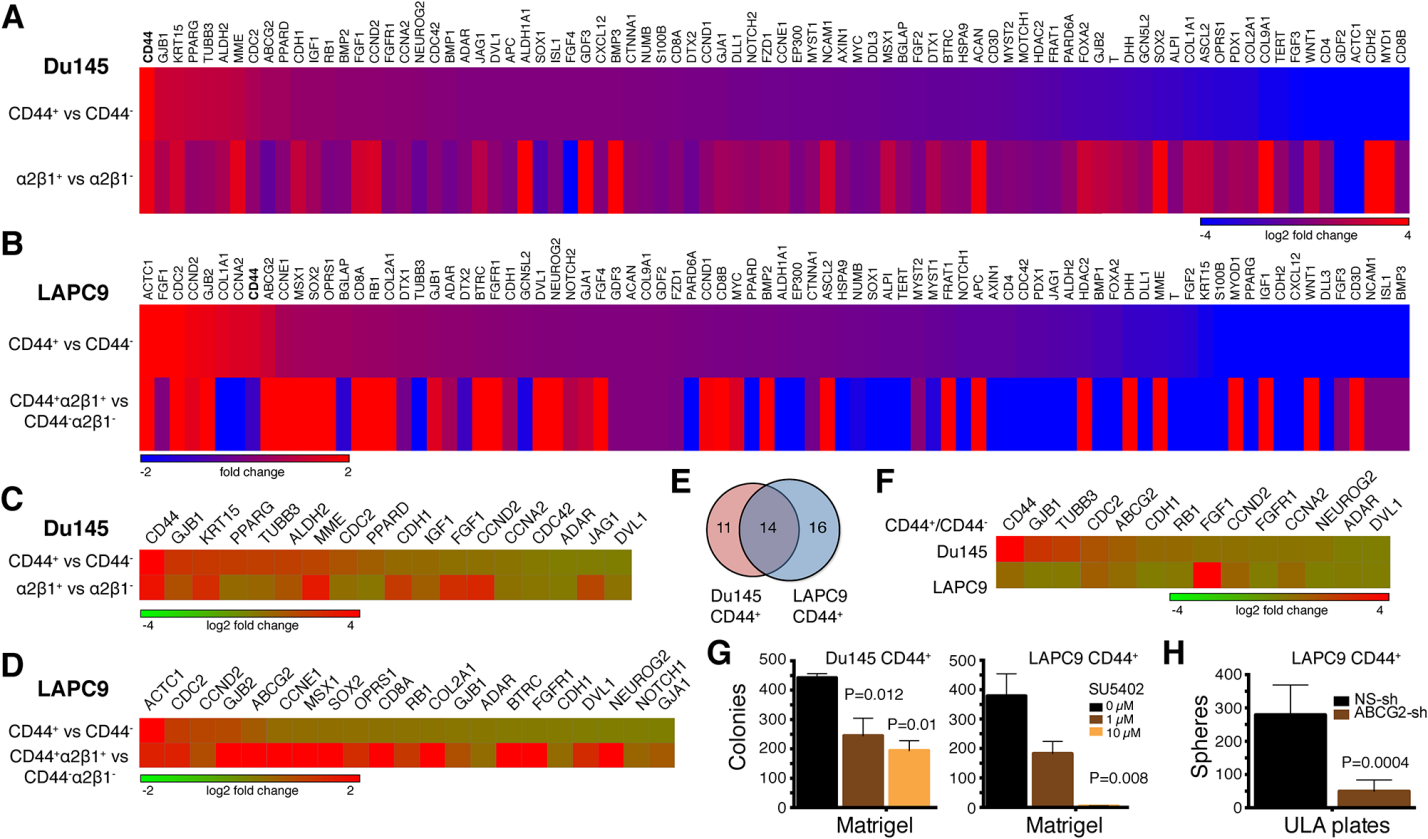
Gene expression profiles and functional studies in PCa cell subpopulations **A–B.** Expression of 84 SC-related genes in the indicated marker-positive and corresponding marker-negative Du145 (A) and LAPC9 (B) cells. Relative expression levels were normalized to the average expression levels of 5 internal controls (B2M, HPRT1, RPL13A, GAPDH and ACTB). Scale bars depict fold changes (in log 2 ratio), centered at 0. For both Du145 and LAPC9, genes were presented from the highest to lowest in the CD44^+^ population. Note that CD44 gene (bold) was the highest expressed gene in Du145 and was among the highest in LAPC9. **C–D.** Heat map of representative genes commonly overexpressed in the two indicated PCa cell populations in Du145 (C) and LAPC9 (D) models. **E–F.** Venn diagram (E) and heat map (F) presenting the genes that were commonly overexpressed in the CD44^+^ Du145 and LAPC9 cells. **G.** Blocking FGFR signaling compromised clonogenic capacity of CD44^+^ PCa cells. Freshly purified Du145 and LAPC9 CD44^+^ cells were plated in Matrigel-coated 12-well plates (3,000 cells/well) and treated with 0 – 10 μM FGFR inhibitor SU5402. Colonies were enumerated 2 weeks after plating. **H.** Knocking down ABCG2 reduced sphere formation in CD44^+^ LAPC9 cells. Freshly purified CD44^+^ LAPC9 cells were infected with non-silencing (NS) or ABCG2 shRNAs (MOI 20) and 48 later, plated in 6-well ULA plates (2,000 cells/well). Spheres were counted 2 weeks after plating.

To further dissect PCSC heterogeneity at the molecular level, we custom-made a RT^2^ Profiler™ qPCR Human Stem Cell Superarray that contained 84 stem cell-associated genes (Supplementary Table 5) and analyzed their expression levels in CD44^+^, α2β1^+^, and/or CD44^+^α2β1^+^ Du145 and LAPC9 cell populations (Figure 9A–9F; Supplementary Figure 11A–1B). The results revealed several interesting findings. First, we observed both overexpressed and downregulated genes in marker-positive in comparison to the corresponding marker-negative populations in both models. Second, we observed similarities as well as differences in gene expression both between different subpopulations of cells in the same cell type and between the same subpopulations of different PCa cell types. For instance, the CD44^+^ Du145 cells displayed a gene expression pattern that was overall different from that in the α2β1^+^ Du145 cells (Figure 9A). Gene expression patterns in CD44^+^ versus CD44^+^α2β1^+^ LAPC9 cells were also dissimilar (Figure 9B). Third, the two subpopulations from the same cell type, however, did share some gene expression patterns. For example, the CD44^+^ and α2β1^+^ Du145 cells (Figure 9C) and the CD44^+^ and CD44^+^α2β1^+^ LAPC9 cells (Figure 9D) shared many overexpressed genes. Fourth, the CD44^+^α2β1^+^ LAPC9 cells, which were among the most tumorigenic and were more tumorigenic than CD44^+^ LAPC9 cells (Table 2), showed more upregulated genes (Figure 9B and 9D; Supplementary Figure 11A). Among the most highly upregulated genes in CD44^+^α2β1^+^ LAPC9 cells were MME (CD10), CCNE1, COL2A1, and those involved in Wnt signaling (FRAT1, BTRC, APC, WNT1), growth factor signaling (FGFR1, IGF1, BMP2, FGF4, NEUROG2), and pluripotency (SOX2) (Supplementary Figure 11A). Many of these molecules are well-known stem cell regulators and have been implicated in PCa etiology and progression [e.g., 32–36].

The qPCR analysis provided clues about potential involvement of certain signaling pathways in commonly regulating several PCSC populations. For example, the CD44^+^ Du145 and LAPC9 cell populations, both of which were tumorigenic, shared 14 upregulated genes including developmental (FGF1, FGFR1, and DVL1), cell-cycle related (RB1, CDC2, CCND2, and CCNA2), and neuronal (TUBB3 and NEUROG2) genes (Figure 9E–9F). As an example of interrogating the functional significance of the signaling pathways, we treated freshly purified CD44^+^ Du145 and LAPC9 cells with SU5402, a specific FGFR inhibitor and then performed colony formation assays in Matrigel and sphere formation assays in ultra-low attachment (ULA) plates (6–13). SU5402 dose-dependently compromised colony (Figure 9G) and sphere (Supplementary Figure 11C) forming capabilities of both CD44^+^ PCa cell populations.

The qPCR results also provided clues about potential relationships between different PCa cell subpopulations. For instance, the CD44^+^ Du145 cell population was enriched not only in *CD44* mRNA but also mRNAs of *ABCG2* and two ALDH isoforms (*ALDH1A1* and *ALDH2*) and the α2β1^+^ Du145 cells expressed high levels of *CD44* and *ALDH1A1* mRNAs (Supplementary Figure 11B). These results suggest that in the Du145 model, CD44^+^, α2β1^+^, ABCG2^+^, and ALDH^+^ cell populations identify overlapping subsets of tumorigenic cells, which is congruent with phenotypic analysis (Supplementary Figure 12A). Similarly, in the LAPC9 model, *ABCG2* mRNA was enriched in both CD44^+^ and CD44^+^α2β1^+^ cell populations (Supplementary Figure 11B), again suggesting that these markers identify overlapping cell populations as corroborated by the flow analysis (Supplementary Figure 12B). Interestingly, the mRNAs of *ALDH1A1* and *ALDH2* were not enriched in the two CD44^+^ LAPC9 populations (Supplementary Figure 11B) but the ALDH^+^ cells were nearly completely encompassed in the CD44^+^ population of LAPC9 cells (Supplementary Figure 12B), suggesting that other ALDH isoform(s) might be involved in mediating the Aldefluor phenotype in the LAPC9 model.

Finally, we employed lentiviral-mediated knockdown to investigate the functions of CD44, integrin α2, and ABCG2 in purified CD44^+^ Du145 and/or LAPC9 cells. CD44 knockdown did not affect the colony or sphere formation in either model (Supplementary Figure 11D; data not shown). These results are consistent with our earlier studies demonstrating that anti-CD44 antibodies did not interfere with the clonal and clonogenic properties of CD44^+^ PCa cells [7]. In contrast to CD44, ABCG2 knockdown inhibited clonogenic activities of both LAPC9 (Figure 9H; Supplementary Figure 11E) and Du145 (not shown) CD44^+^ cells, which is consistent with ABCG2 enrichment and also suggests its functional significance in the two CD44^+^ PCa cell populations. Interestingly, knocking down integrin α2 also strongly suppressed the clonogenicity of LAPC9 CD44^+^ cells (Supplementary Figure 11D).

### Clonogenic and tumorigenic subpopulations in untreated patient tumors

**Figure 10:**
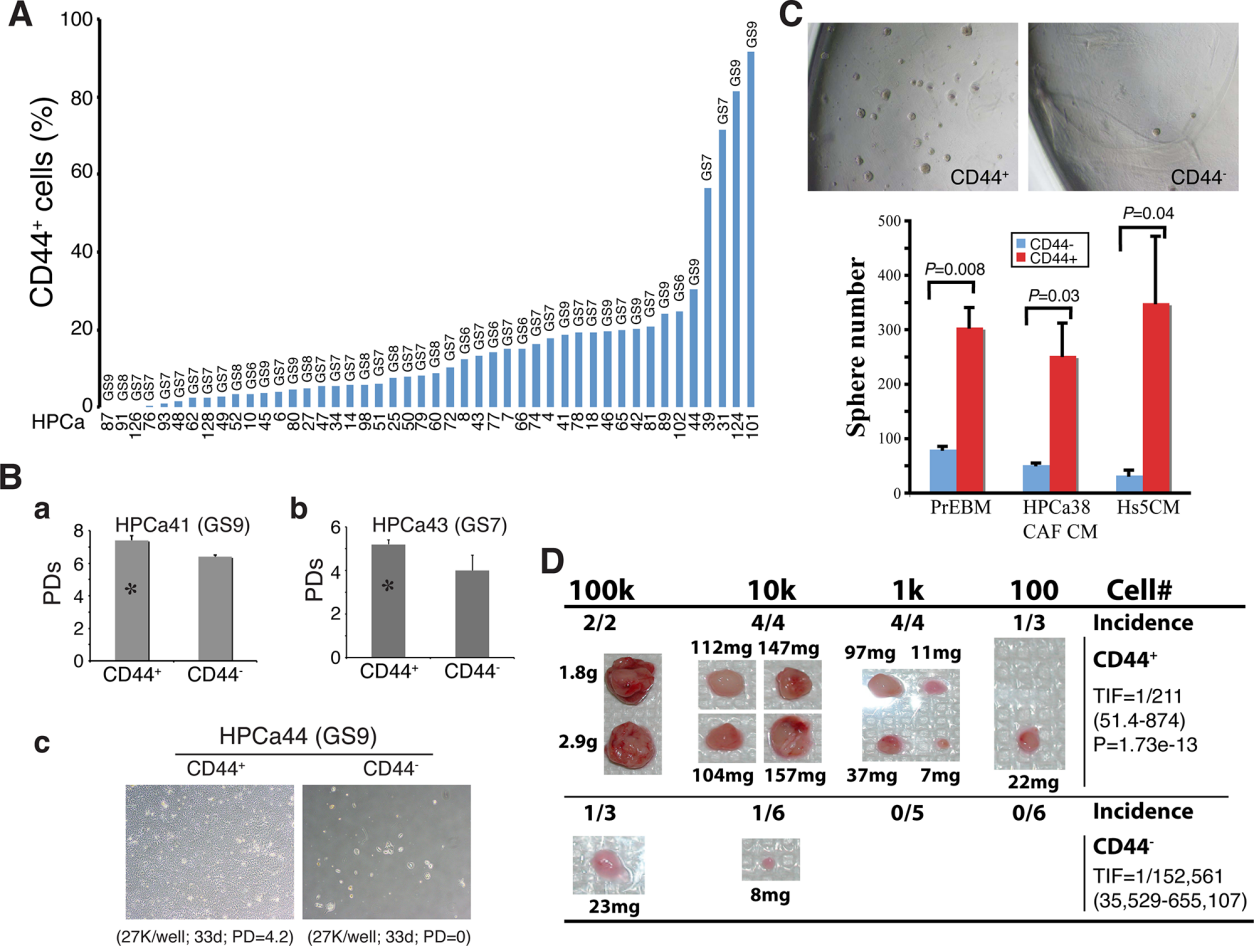
CD44^+^ HPCa cells possess high proliferative, survival, clonogenic, and tumorigenic potential **A.** Percentage of CD44^+^ cells in HPCa samples. The combined Gleason score (GS) for each tumor is indicated on top and the patient ID# at the bottom. **B.** CD44^+^ HPCa cells possess higher proliferative and survival advantages than the corresponding CD44^−^ HPCa cells. a. CD44^+^ and CD44^−^ HPCa41 cells were plated in triplicate on Swiss 3T3 feeder layer (1, 000 cells/well) and cell numbers determined 29 days after plating. Shown are the cumulative population doublings (PDs; *P < 0.05). b. CD44^+^ and CD44^−^ HPCa43 cells were plated in triplicate on Swiss 3T3 feeder layer (5,000 cells/well) and cell numbers determined 41 days after plating. Shown are the cumulative PDs (*P < 0.01). c. Purified CD44^+^ and CD44^−^ HPCa44 cells were plated in triplicate on collagen-coated 6-well dishes. Shown below are the cell numbers plated, time when surviving cells were enumerated, and the cumulative PDs. **C.** CD44^+^ HPCa cells possess high clonogenic potential. CD44^+^/CD44^−^ HPCa51 cells were plated, in triplicate, in Matrigel-coated 12-well plates (10, 000 cells/well). Shown are representative images (40×) of spheres (top) and quantifications of spheres plated in 3 different media (PrEBM, prostate epithelial basal medium; HPCa38 CAF CM, conditioned medium from HPCa38 carcinoma-associated fibroblasts or CAFs; Hs5 CM, conditioned medium from Hs5 immortalized human mesenchymal stem cells) 15 days after plating. **D.** CD44^+^ HPCa cells possess high tumorigenic potential. CD44^+^/CD44^−^ HPCa52 cells were acutely MACS-purified from the patient tumor (GS8) and co-injected, at the indicated cell numbers, with 100k Hs5 cells in 50% Matrigel s.c into irradiated NOD/SCID-γ mice. The 10k and 100k tumors were harvested at ∼4 months whereas 100 and 1k tumors were harvested at 7 months after implantation. Shown on the right are the TIF for the two populations and the *P* value for TIF comparison.

We showed earlier that untreated primary human PCa (i.e., HPCa) contained CD44^+^, α2β1^+^, and ALDH1A1^+^ cells that were mostly PSA^−/lo^ (Figure 7; Supplementary Figure 5E). Here, we quantitatively analyzed the expression and, importantly, potential functions of PCSC marker-positive HPCa cells, i.e., CD44^+^, α2β1^+^, CD44^+^α2β1^+^, and ALDH^+^, as well as CD133 [15] in a large cohort (∼50) of HPCa samples (Figure 10; Supplementary Figure 13; Supplementary Table 2). The majority of the HPCa samples we examined (44/46, 96%) contained CD44^+^ cells, although the percentages varied widely (Figure 10A; Supplementary Table 2). When CD44^+^ HPCa cells, which were all negative for AR and PSA proteins (Supplementary Figure 13A) as we previously observed [8, 37, 38], were purified out, plated on fibroblast feeders or collagen, and analyzed for their proliferative potential, we observed higher population doublings (PDs) for the CD44^+^ cell population than CD44^−^ population in HPCa41 (Figure 10Ba), HPCa43 (Figure 10Bb), HPCa44 (Figure 10Bc), HPCa50 (Supplementary Figure 13C), and HPCa51 (not shown) samples. In fact, most HPCa44 (Figure 10Bc) and HPCa50 (Supplementary Figure 13C) CD44^−^ cells initially attached but did not proliferate and soon died off whereas the corresponding CD44^+^ cells expanded exponentially, suggesting that the CD44^+^ HPCa cells also possess greater survival advantage. On the other hand, as we observed in the xenograft systems, not all patient-derived CD44^+^ HPCa cells manifested higher proliferative potential than their CD44^−^ counterparts (e, g., Supplementary Figure 13D).

We also compared the clonogenic potential of CD44^+^/CD44^−^ HPCa cells by plating them, at clonal density, in Matrigel in several variations of serum-free medium. The results revealed significantly higher sphere-forming ability of the CD44^+^ cells from HPCa50 (not shown) and HPCa51 (Figure 10C) than the respective CD44^−^ HPCa cells. The above clonal and clonogenic assays indicate that primary CD44+ HPCa cells possess certain stem/progenitor cell properties, which was supported by the expression of stem cell marker hTERT (Supplementary Figure 13A; data not shown). Importantly, in a pilot *in vivo* experiment, we purified out CD44^+^/CD44^−^ cells from HPCa52 (GS8) and co-injected them, at increasing cell numbers, with the Hs5 mesenchymal cells [31], subcutaneously in irradiated male NOD/SCID-γ mice supplemented with the exogenous testosterone. As shown in Figure 10D, the CD44^+^ HPCa52 cells demonstrated higher tumor-regenerating capacity than corresponding CD44^−^ cells. This was quite a remarkable finding for the bulk primary HPCa cells are known to be extremely indolent in tumor regeneration [11, 31].

HPCa also expressed other PCSC markers including CD133 [15]. In general, the % of CD133^+^ HPCa cells was lower than that of CD44^+^ HPCa cells (Supplementary Figure 13E; Supplementary Table 2). The CD133^+^ LAPC4 (Supplementary Figure 13F) and HPCa (Supplementary Figure 13G–13H) cells showed higher proliferative and sphere-forming potential than the corresponding CD133^−^ cells. Interestingly, in a pilot study we observed higher *CD44* and integrin *α2* mRNA levels in CD133^+^ HPC40 cells than the corresponding CD133^−^ cells (Supplementary Figure 13I), suggesting a potentially overlapping relationship among the 3 subpopulations in HPCa samples.

Together, these results suggest that untreated primary tumors contain subsets of HPCa cells that express the phenotypic markers of PCSCs and possess enhanced clonal, clonogenic, and even tumorigenic potential.

## DISCUSSION

To our knowledge, the present study represents the most comprehensive efforts to dissect the phenotypic, functional, and tumorigenic heterogeneities in human PCa cells using multiple xenograft models and > 70 patient tumor samples. In the first part, we further investigate the PSA^−/lo^ PCa cell population, which we have recently shown to harbor self-renewing long-term tumor-propagating cells [13]. We demonstrate that 1) tumor cell *PSA* mRNA levels inversely correlate with grade, metastasis, and patient survival; 2) discordant AR and PSA expression in both untreated and castration-resistant PCa (CRPC) results in AR^+^PSA^+^, AR^+^PSA^−^, AR^−^PSA^−^, and AR^−^PSA^+^ subtypes of PCa cells that manifest differential sensitivities to therapeutics; 3) the PSA^−/lo^ PCa cells pre-exist in untreated primary tumors and castration leads to a great enrichment of PSA^−/lo^ PCa cells in both xenograft tumors and CRPC samples; 4) the PSA^−/lo^ PCa cells are quiescent and resistant to castration and other stress treatments; 5) systemic androgen levels dynamically regulate the relative abundance of PSA^+^ versus PSA^−/lo^ PCa cells in the tumors that impacts the kinetics of tumor growth; 6) the PSA^−/lo^ PCa cells seem to possess distinct epigenetic profiles; and 7) the PSA^−/lo^ PCa cell population is enriched in several CSC markers including CD44, integrin α2β1, and ALDH1A1.

Heterogeneous and discordant AR and PSA expression in PCa cells has been reported in numerous earlier studies [39–61]; however, our study, for the first time, has proposed and presented the evidence for the 4 subtypes of PCa cells, i.e., AR^+^PSA^+^, AR^−^PSA^+^, AR^+^PSA^−^, and AR^−^PSA^+^ that pre-exist in untreated HPCa. We have shown preliminary evidence that 3 LNCaP sublines representing 3 subtypes of PCa cells, i.e., AR^+^PSA^+^ (regular LNCaP), AR^+^PSA^−^ (LNCaP-abl) and AR^−^PSA^−^ (LNCaP-CDSS and LNCaP-MDV) exhibit differential responses to antiandrogens, chemodrugs, and targeted therapeutics. Of clinical significance, the PSA^−/lo^ cell population, which encompasses both AR^+^PSA^−/lo^ and AR^+^PSA^−/lo^ cells, becomes strikingly enriched in all CRPC samples examined and in castration-resistant xenograft model. These analyses, taken together with evidence of distinct epigenetic profiles of PSA^−/lo^ vs. PSA^+^ subsets, suggest that castration selects for undifferentiated PSA^−/lo^ PCa cells.

Our previous work has demonstrated that the PSA^−/lo^ PCa cell population harbors self-renewing long-term tumor-propagating PCSCs that resist castration [13]. The present study follows up on the earlier work by further showing that the PSA^−/lo^ PCa cells are much more quiescent than the PSA^+^ cells, based on time-lapse tracking of single cells and clonal analysis. Purified PSA^−/lo^ PCa cells, like the bulk AR^−^PSA^−/lo^ LNCaP subline, are also refractory to antiandrogens and other drugs. We further demonstrate that the relative abundance of both PSA^−/lo^ and PSA^+^ PCa cells in tumors are regulated dynamically by systemic androgen levels, which in turn impacts tumor regeneration and growth in androgen-proficient versus androgen-deficient conditions. These latter observations implicate differential epigenetic mechanisms in regulating the two populations of PCa cells. In support, targeted ChIP/re-ChIP assays on 8 gene promoters known to be associated with bivalent chromatin domains in ES cells reveal 4 genes possessing bivalent features but preferentially in PSA^−/lo^ PCa cells, consistent with these cells possessing stem cell gene expression profiles and biological characteristics [13]. A genome-wide ChIP-Seq analysis of several histone marks in purified PSA^−/lo^ and PSA^+^ PCa cells is under way.

The PSA^−/lo^ PCSC population is heterogeneous [13]. Therefore, in the second part of this project, we carried out exhaustive tumor-regeneration and serial transplantation studies in 2 AR^+^/PSA^+^ (LAPC9 and LAPC4) and 2 AR^−^/PSA^−^ (PC3 and Du145) PCa models. The results provide indisputable evidence that 1) different PCa models possess distinct profiles of tumorigenic subpopulations; 2), some PCa (e.g., LAPC9 and Du145) may possess several populations of CSCs whereas others (e.g., LAPC4) seem to have a paucity of CSC populations; 3) no single marker profile can track tumor-propagating cells in all models; and 4) the ability of combinatorial marker-sorting strategy to further enrich CSCs over single marker strategies is dependent on the cancer models analyzed (Supplementary Table 2). Therefore, the CD44^+^ phenotype enriches CSCs in Du145 and LAPC9 but not in LAPC4 models whereas the ALDH^+^ phenotype enriches tumor-initiating cells in all 4 models except LAPC4. Similarly, the CD44^+^α2β1^+^ phenotype enriches CSCs in LAPC9 and LAPC4 but not in Du145 models. These results provide essential foundation for understanding CSC heterogeneity [1, 2] and also explanations to why different groups, working on individual PCa models, have often reported divergent PCSC phenotypes.

That tumorigenic subpopulations can be enriched by several different markers and functional strategies implies that some tumors contain a CSC pool with heterogeneous tumorigenic subsets that possess distinct tumor-initiating and tumor-propagating properties. In support, the LAPC9 model harbors tumorigenic subpopulations that can be prospectively enriched using CD44^+^ and CD44^+^α2β1^+^ profiles as well as the SP and ALDH assays with the CD44^+^α2β1^+^ subpopulation being the most tumorigenic (i.e., ∼1 tumor-initiating cell in every 20 cells; Table 2). Detailed phenotypic and molecular profiling in Du145 and LAPC9 models shows that the CD44^+^, α2β1^+^, ABCG2^+^, and ALDH^+^ cell populations identify overlapping subsets of tumor-initiating cells. Functional interrogation demonstrates that integrin α2 and ABCG2 but not CD44 are causally important for the clonal and clonogenic properties of the CD44^+^ PCa cells. The results with CD44 suggest that the molecule probably regulates PCSC properties in some other ways. Indeed, we have recently shown that CD44 plays a critical role in facilitating the invasive and metastatic behavior of PCSCs [12]. Of significance, the tumorigenic CD44^+^ cell populations in both Du145 and LAPC9 commonly upregulate 14 genes involved in development (FGF1, FGFR1, and DVL1), cell cycle (RB1, CDC2, CCND2, and CCNA2), and neuronal activity (TUBB3 and NEUROG2), providing potential therapeutic targets for the CD44^+^ PCa cells. Similar molecular profiling reveals genes preferentially expressed in the most tumorigenic CD44^+^α2β1^+^ LAPC9 cells including Wnt (FRAT1, BTRC, APC, WNT1), growth factor (FGFR1, IGF1, BMP2, FGF4, NEUROG2), and pluripotency (SOX2) signaling molecules. As a proof of principle, an FGFR inhibitor potently blocks the clonal and clonogenic activity in CD44^+^ LAPC9 and Du145 cells.

Our observations in PCa are consistent with the phenotypic heterogeneity and functional diversity of CSCs recently reported in other tumor systems including cancers of the breast, pancreas, and colon as well as acute myeloid leukemia and glioblastoma [1, 2, 25, 62–67]. Our results also support but greatly extend earlier efforts in using CD antigen phenotyping to study PCa cell heterogeneity [68]. Importantly, phenotypic analysis combined with functional studies in ∼50 HPCa samples demonstrate that untreated HPCa samples also heterogeneously express CSC markers including CD44, CD133, α2β1, and ALDH and that prospectively purified CD44^+^ and CD133^+^ HPCa cells in most (though not all) samples manifest high proliferative, clonal and clonogenic capacities.

**Figure 11:**
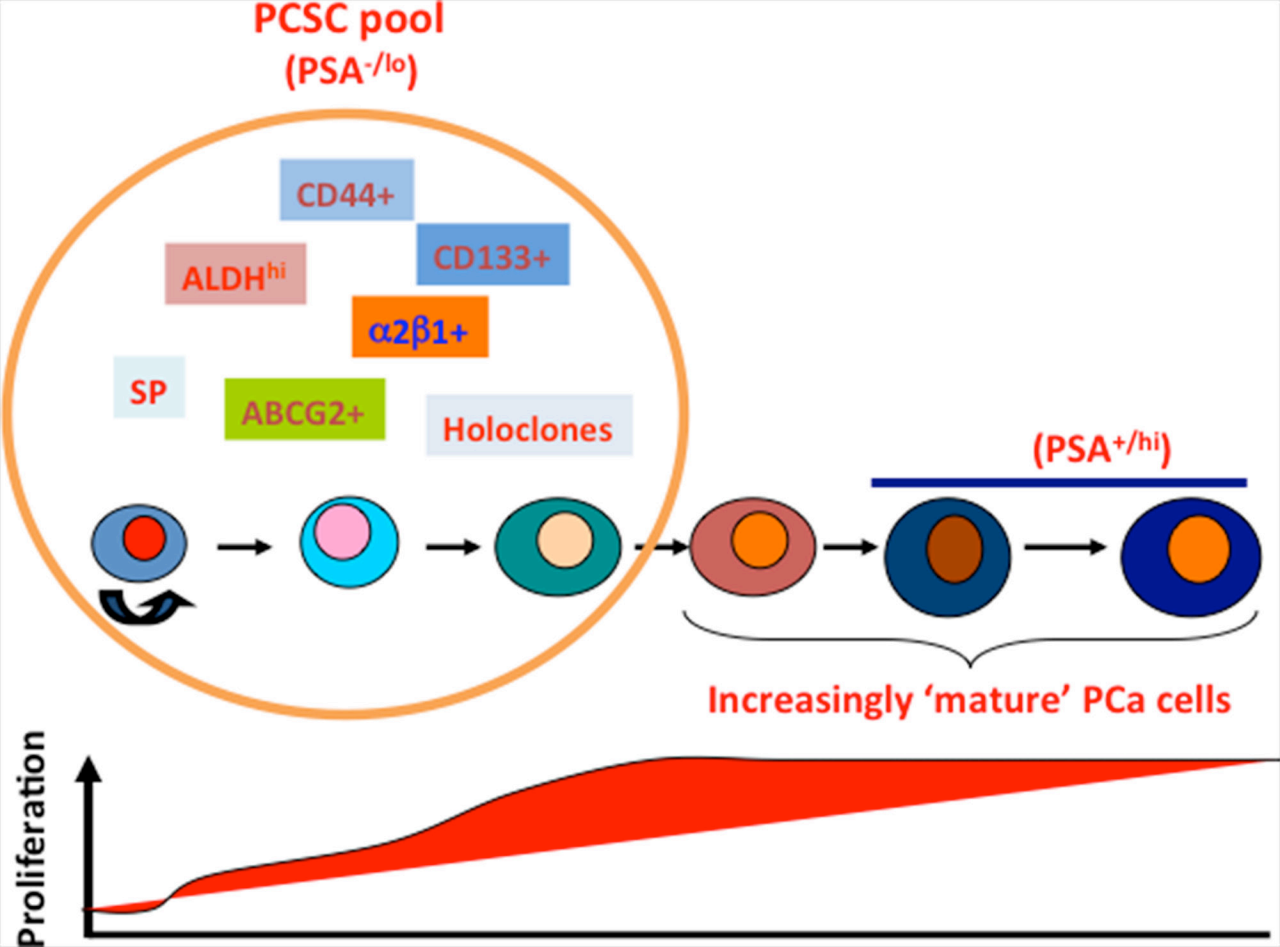
A hypothetical model of tumorigenic heterogeneity of human PCa cells Untreated (hormone-naïve) PCa contain a spectrum of tumor cells at different stages of differentiation (marked by cells of varying colors and sizes). The PCSC pool in these tumors mainly contains undifferentiated (PSA^−/lo^) PCa cells, which are quiescent (thus low proliferative index; below) and can undergo ACD developing into differentiated cells. The PSA^−/lo^ PCa cells possess long-term tumor-propagating activity. The PSA^−/lo^ PCSC pool is heterogeneous harboring and/or overlapping with other tumorigenic subsets that can be prospectively purified out using the marker profiles indicated. The PCSC pool contains the intrinsically castration-resistant cells. In contrast, fully differentiated (PSA^+^) PCa cells, despite being highly proliferative (thus high proliferative index, below), only undergo symmetric cell division and possess more limited tumor-propagating capabilities. The PSA^−/lo^ PCSC pool is relatively small and preexists in untreated patient tumors but dramatically enriched in CRPC in which the profiles of tumorigenic subsets may likely be very different from those in the untreated tumors. This model is updated from our earlier model (8). See Text for more discussions.

Results from the present study reinforce the intrinsic stem cell nature and castration-resistant properties of the PSA^−/lo^ PCa cells. Then what is the relationship between the PSA^−/lo^ PCa cell population and several other populations of PCSCs including CD44^+^, α2β1^+^ and ALDH^+^ PCa cells? IF staining combined with molecular profiling indicate that the 3 CSC marker-positive populations of PCa cells are included in the PSA^−/lo^ population (Figure 7; Supplementary Figure 5). The current work, together with our systematic studies published over the past 10 years [6–13] allows us to propose a hypothetical model that unifies most previous PCSC studies (Figure 11). The model posits that untreated prostate tumors contain a spectrum of cancer cells at different stages of differentiation. Undifferentiated (PSA^−/lo^) PCa cells are quiescent and can undergo ACD to generate PSA^+^ cells whereas the PSA^+^ PCa cells are highly proliferative but only undergo SCD (Figure 11). The PSA^−/lo^ PCa cells possess unlimited whereas PSA^+^ PCa cells limited tumor-propagating activity [13]. The PSA^−/lo^ PCa cells are intrinsically more resistant to castration and other therapeutics than PSA^+^ cells [this study; 13]. Importantly, the PSA^−/lo^ PCa cell population is heterogeneous harboring and/or overlapping with other tumorigenic subsets including the SP, holoclones, and ALDH^+^, CD44^+^, α2β1^+^, and ABCG2^+^ cells [6–8, 10, 12, 13; this study] (Figure 11) and, likely, other subsets such as CD133^+^ [15] and TRA-1–60^+^CD151^+^CD166^+^ cells [19], which are AR^−^PSA^−^.

Our model (Figure 11) also provides a framework for understanding PCa cell heterogeneity and potential cell-of-origin to CRPC. Therefore, untreated primary HPCa, like LNCaP, LAPC9, and LAPC4 xenografts, all contain a major population of PSA^+^ cells but also a minor PSA^−/lo^ population, in which tumorigenic subsets differ both quantitatively and qualitatively depending on individual HPCa samples and xenograft models. Due to the nearly homogeneous AR expression in PSA^+^ PCa cells (Supplementary Figure 4A; 13), primary HPCa and AD xenografts respond well to antiandrogens, leading to prominent reduction in tumor burden. On the other hand, the PSA^−/lo^ PCa cells, being only ∼30% AR^+^, do not respond well to antiandrogens and will survive ADT leading to the eventual emergence of CRPC. In support, the PSA^−/lo^ PCa cells from multiple PCa models regenerate AI tumors very efficiently in completely androgen-deficient hosts [13]. Significantly, more tumorigenic subsets can be further purified out from the PSA^−/lo^ PCa cell population to establish CRPC [13; Chen *et al*., manuscript submitted]. These observations implicate the PSA^−/lo^ PCa cell population, which pre-exists in primary HPCa, as a cell-of-origin for CRPC due to their preferential survival of castration. This conjecture is fully consistent with classical studies performed decades ago reporting that CRPC might result from selective outgrowth of pre-existent AI clones in primary tumors [69, 70]. In contrast to the untreated HPCa and AD xenografts, the cellular landscape in clinical CRPC and AI xenografts completely changes with the PSA^−/lo^ cells becoming predominant [this study, 13]. The profiles of tumorigenic subsets within the PSA^−/lo^ PCSC pool may also likely to change (Figure 11). Taken together, the current study further highlights the need to develop novel therapeutics that specifically target the PSA^−/lo^ population and other PCSC subsets within, which when used in combination with ADT, should help prevent tumor relapse. Many of our ongoing projects are fulfilling this need.

## MATERIALS AND METHODS

### Cells and antibodies

PC3, Du145, PPC-1, LNCaP cells were obtained from ATCC (Manassas, VA) whereas 293FT packaging cells were purchased from Invitrogen (Carlsbad, CA), respectively. All these cells were mycoplasma free, STR-authenticated, and routinely maintained in serum- and antibiotic-containing media as suggested by the providers. Synthetic androgen R1881 and androgen antagonist bicalutamide were purchased from PerkinElmer (cat# NLP005005MG; Waltham, MA) and Toronto Research Chemicals (cat#B382000; Ontario, Canada), respectively. MDV3100 was bought from Selleck Chemicals (car# S1250). All other chemicals were obtained from Sigma unless otherwise specified. Antibodies used in the present study included:

mouse mAb to integrin α2β1 (cat# MAB1998Z, clone BHA2.1; Chemicon, Billerica, MA)

mouse mAb to β-actin (cat# 69100, clone C4; ICN, MP Biomedicals, Solon, OH)

rabbit pAb to ABCG2 (cat# AV43649; Sigma)

mouse mAb to AR (cat# sc-7305, clone 441; Santa Cruz Biotechnology, Santa Cruz, CA)

mouse mAb to Bcl-2 (clone N-19; Santa Cruz Biotech)

mouse mAb to Bcl-2 (cat# 610538; BD Biosciences, San Jose, CA)

mouse mAb to BrdU (cat# B2531, clone BU-33; Sigma, St Louis, MO)

mouse mAb to CD44 (cat# 550932, clone G44–26; BD Biosciences)

mouse mAb to CD44 (cat# sc-7297; Santa Cruz Biotech.)

rabbit pAb to cytokeratin 5 (cat# PRB-160P; BAbCO, Covance, Princeton, NJ)

mouse mAb to cytokeratin 18 (cat# 550511, clone GRE53; BD Biosciences)

mouse mAb to cytokeratin 18 (cat# MAB1600, clone DC-10; Chemicon)

rabbit mAb to GAPDH (cat# sc-25778, clone FL-335; Santa Cruz Biotech)

rabbit pAb to GFP (cat# Ab290; Abcam)

rabbit pAb to Ki-67 (cat# Ab16667; Abcam)

mouse mAb to P63 (cat# sc-8431; clone 4A4; Santa Cruz Biotechnology)

rabbit pAb to PSA (cat# A0562; Dako, Carpinteria, CA)

mouse mAb to PSA (clone A67-B/E13; Santa Cruz Biotechnology)

rabbit pAb to Histone H3 (Cat# 06–755, Millipore)

rabbit pAb to Histone H3K4, trimethyl (cat# 07–473, Millipore)

rabbit pAb to Histone H3K27, trimethyl (cat# 07–449, Millipore)

mouse mAb to Histone H3K27, trimethyl (cat# 61017, Active Motif)

rabbit control IgG, ChIP grade (cat# ab46540, Abcam)

rabbit pAb to hTERT (cat# NB 100–141; Novus)

Alexa Flour 405 conjugated streptavidin (S32351, Invitrogen)

Alexa Flour-conjugated secondary antibodies (Invitrogen)

APC-conjugated goat anti-mouse IgG (550826; BD Biosciences)

Biotin-conjugated pAb to mouse H-2K^d^ (SF1–11; BD Biosciences)

PE-conjugated mAb to H-2K^d^ (clone SF1–1.1; BD Biosciences)

PE conjugated mAb CD44 antibody (550932, BD Bioscience)

### Regular immunohistochemical (IHC) staining and double immunofluorescence (IF) staining of AR and PSA in formalin-fixed paraffin-embedded (FFPE) HPCa sample

Basic IHC protocols have been described [12, 13]. Paraffin-embedded sections (4 μm) were deparaffinized and hydrated in xylene followed dehydration in graded alcohols to water. Antigen retrieval was performed in 1.0 mM EDTA Buffer (pH 8.0) for 10 min in a microwave oven followed by a 20-min cool down. Slides were then incubated with various primary antibodies followed by Envision-plus labeled polymer-conjugated horseradish peroxidase and DAB monitoring staining development (Dako). For IHC analysis of PSA^+^ and PSA^−/lo^ cells in FFPE HPCa sections, we first titrated the primary antibody to PSA (A0526, Dako) and found that at 1:5 dilutions, the antibody reliably differentiated the PSA^+^ and PSA^−/lo^ PCa cells. We then utilized this antibody concentration to stain FFPE sections [13]. In general we stained at least 3 consecutive sections from each sample for PSA. Twelve fields were chosen from each slide for counting by two individuals in a blind fashion and PSA^+^ and PSA^−/lo^ PCa cells were averaged.

For PSA and AR double IF staining, HPCa sections (4 μm) were deparaffinized and dehydrated through graded alcohols. Antigen retrieval was performed by soaking slides in pre-warmed target retrieve agent (S1099, Dakocytomation) in boiling water bath (40 min). Slides were incubated with Background Sniper (BS966H, Biocare Medical) at room temperature for 30 min. For primary antibody staining, slides were incubated at 4°C overnight with a mix of mouse monoclonal anti-AR (clone 411, SC-7305, Santa Cruz Technology; 1:50) and rabbit polyclonal anti-PSA (A0526, Carpinteria, CA; 1:5) in PBS containing 0.1% Triton and 5% goat serum. After thorough washing, slides were incubated at RT for 60 min with secondary antibodies (Invitrogen), i.e., Alexa Flour 594-conjugated goat anti-mouse IgG (1:500) and Alexa Flour 488-conjugated goat anti-rabbit IgG (1:500) in PBS plus 0.1% Triton and 5% serum, followed by thorough washing. Then slides were incubated with DAPI (3 μM) diluted in PBS (RT for 5 min). To eliminate autofluorescence, slides were immersed in 70% ethanol for 5 min, incubated in Autofluorescence Eliminator Reagent (2160, Millipore) for 5 min, and were finally passed through 3 changes of 70% ethanol for 1 min each. Upon rinsing in PBS, slides were mounted with 10 μL Gold Antifade Reagent (936590, Prolong). Images were acquired on an Olympus microscope.

### Xenograft tumor processing and purification of human PCa cells from xenografts

Basic procedures were detailed elsewhere [11]. Briefly, xenograft tumors were harvested from maintenance tumors and minced into ∼1 mm^3^ pieces, which were rinsed once with PBS, digested for 30 min with Accumax (AM105; Innovative Cell Technologies, San Diego, CA) at room temperature, and filtered though 40-μm cell strainer. Dead cells and debris were separated from live cells on a discontinuous Percoll gradient. Lineage-positive mouse cells were depleted using either MACS Lineage Cell Depletion Kit (Miltenyi Biotec) or staining for mouse-specific MHC using PE or Biotin-conjugated monoclonal anti-H-2K^d^ (SF1–11; BD Biosciences).

### Primary prostate tumor (HPCa) processing

Our lab has so far worked on >220 HPCa samples and the present study utilized >70 HPCa samples (Supplementary Table 2). All HPCa samples (with the matched normal/benign samples) were obtained with the written informed consent from the patients in accordance with federal and institutional guidelines and with the approved IRB protocols (MDACC LAB04–0498). HPCa processing protocol has been described previously [11–13]. Lineage-positive (i.e., hematopoietic, endothelial, smooth muscle, fibroblast, and other stromal) cells were depleted using the MACS Lin-1 cocktail mix and anti-CD140b-PE (Miltenyi Biotec). Purified HPCa cells were used in multiple types of experiments and, in some cases, for infection with the PSAP-GFP lentiviral vector. When necessary, HPCa cells were cultured for a short period time in various media, e.g., serum/androgen-free PrEBM supplemented with insulin, EGF, and bovine pituitary extract.

### Tumor experiments and serial tumor transplantation in NOD/SCID mice

Subcutaneous (s.c) and orthotopic (i.e., dorsal prostate or DP) tumor transplantations were carried out as previously described [6–8, 11–13]. For serial tumor transplantations in NOD/SCID mice, marker-positive and -negative PCa cells were sorted out by FACS from the first-generation (1°) tumors originally derived from corresponding marker-positive and –negative cells, and implanted s.c or in the DP to generate secondary (2°) tumors. Sequential tumor transplantation was performed using similar strategies. For tumor experiments in castrated mice, male NOD/SCID mice (6–8 weeks) were surgically castrated 1–2 weeks prior to tumor cell injection.

### Lentiviral infection of PCa cells

Lentivirus was produced in 293FT packaging cells and titers determined using GFP positivity in HT1080 cells. PCa cells were infected, generally, at a multiplicity of infection (MOI) of 20 and harvested at 48–72 h post-infection. Infected bulk cells or FACS-purified subpopulation of cells were used in various *in vitro* and *in vivo* experiments detailed in each Figure.

### Fluorescence-activated cell sorting (FACS)

PCa cells stained for various markers or after PSAP-GFP infection (48–72 h) were dissociated into single-cell suspension and generally 1–10 × 10^6^ cells were used for FACS on a BD FACSAria™ Fusion cell sorter. Unstained or uninfected cells were used as negative control for gating. Post-sort analysis was routinely performed to guarantee the purity of each population. HPCa cells freshly purified from primary tumors were first infected with PSAP-GFP and sorted 3–7 days later. To purify marker-positive PCa cells from xenograft tumors, we first incubated PCa cells with FcR blocking agent (Miltenyi Biotec) for 15 min at 4°C and then stained them with various primary antibodies. For double or triple marker populations, we would incubate cells with anti-α2β1 (MAB1998Z; Chemicon) for 30 min on ice followed by staining with APC-conjugated goat anti-mouse IgG (550826; BD Biosciences) for 15 min on ice. Cells were then washed (3x) and stained with PE-conjugated anti-CD44 antibody (550932, BD Bioscience) and biotinylated mouse H2-Kd (553564, BD Pharmingen) for 20 min. After washing, cells were incubated with Alexa Flour 405-conjugated streptavidin for 10 min at 4°C. Cells were incubated in solution containing 1% BSA and 2.5 μg/ml insulin (I-6634, Sigma). PCa cells were suspended in ALDEFLUOR assay buffer containing ALDH substrate (1 μM per 1 × 10^6^ cells, the ALDEFLUOR kit; StemCell Technologies, Vancouver, Canada) and incubated for 40 min at 37°C and sorted by FACS. As negative control, we added 50 nmol/l diethylaminobenzaldehyde (DEAB) to the cell suspension.

### Clonal and clonogenic sphere-formation assays

Holoclone and sphere-formation assays were conducted as previously described [10, 11] and stringent conditions were employed to ensure that clones, colonies, and spheres were all derived from single cells [12]. Briefly, we performed clonal analysis using purified and/or sorted PCa cells plated at 100 cells/10-cm plate or 100 cells/well in a six-well culture dish. Clones with ≥50 cells were scored ∼2 weeks after plating. We performed LAPC9 clonal analysis on mitomycin C (M0530, Sigma) treated Swiss 3T3 cells. The results were expressed as cloning efficiency (%). In some clonal assays, cells were directly sorted into 96-well plates at 1 cell/well and clonal type and size were monitored and scored under a (fluorescence) microscope [10, 11]. For clonogenic sphere-formation assays in xenograft and HPCa cells, cells were plated at 5,000–10,000 cells/well in six-well culture dishes coated with a thin layer of 1% solidified agar or 50% Matrigel or plated in 6-well ultra-low attachment (ULA) plates. Spheres that arose within 1–2 weeks were presented as clonogenicity (%). For serial sphere-formation assays, the first-generation spheres were harvested, disaggregated with 0.025% trypsin/EDTA, triturated with a 27-G needle, filtered through 40-μm mesh, and replated as above. This process was repeated for up to 4–5 generations. We sometimes performed serial clonogenic assays in a different way. Briefly, cells were first resuspended in DMEM/F12 supplemented with B27 (17504–044, Invitrogen) and N2 (17502–048, Invitrogen) and mixed (7:4) thoroughly with methylcellulose (04100, Stem Cell Technology) and plated (600 μl) in 24-well ULA plates at 2, 000 cells/wells. Primary spheres were scored in ∼2 weeks. For secondary sphere assays, the first-generation spheres were individually picked up with a transfer pipette under a dissection microscope and dissociated with 0.05% trypsin/EDTA. All the cells derived from individual spheres were mixed with methylcellulose and plated back to one well of a 96-well ULA plate.

### Clonogenic assays in CD44^+^ PCa cells treated with FGFR inhibitor or with gene knockdowns

Du145 and LAPC9 cells were incubated with PE-conjugated anti-CD44 (BD Biosciences; 1:10 dilution) for 1 h at 4°C. Cells were washed and resuspended in sorting buffer, and the CD44^+^ (top 20%) population was sorted out by FACS (see above). For shRNA (CD44, integrin α2, and ABCG2) infection, 1 × 10^5^ sorted cells were plated in a 12-well plate in PrEBM media supplemented with B27, 10 μM EGF, 10 μM FGF, 8 μg polybrene and infected with individual shRNA lentiviral vectors (MOI 20). After 48 h, 2 × 10^3^ cells were plated per well in a 6-well ULA plate in complete PrEBM media and incubated for 2 weeks before scoring. Alternatively, cells were resuspended in a 1:1 ratio of Matrigel:PrEBM media mix and 3 × 10^3^ cells per well were plated around the rim in a 12-well plate. After the Matrigel solidified, 2 ml of complete PrEBM media was added to wells and cells were incubated for 2 weeks before scoring. For treatment with the FGFR inhibitor SU5402, CD44^+^ cells were plated in both Matrigel and ULA plates immediately after sorting as described above with the indicated SU5402 concentrations.

### Immunofluorescence (IF) microscopy

Basic IF procedures have been described [6–8]. To correlate GFP and AR expression in LNCaP cells, GFP^+^ and GFP^−/lo^ cells were sorted out by FACS and plated on the glass coverslips overnight. Cells were stained using a monoclonal antibody to AR (clone 441) followed by goat anti-mouse IgG conjugated to Alexa Fluor 594. For Ki-67 staining, cells were sorted out via FACS and plated on the glass coverslips for 8 h. Cells were then incubated with the rabbit mAb to Ki-67 (Abcam; 1:1000) for 60 min at room temperature. Following thorough washing for 3 times with PBS, the coverslips were incubated for 60 min at room temperature with Alexa Fluor 594–conjugated goat anti-rabbit IgG (1:1000). In some experiments, freshly purified CD44+ HPCa cells were plated on collagen-coated glass coverslips and cultured in PrEBM media supplemented with B27, 10 μM EGF, 10 μM FGF, and BPE overnight followed by IF labeling for CD44, α2β1, AR, PSA, and hTERT.

### Cell cycle and cell death analyses

To determine the cell-cycle profiles, regular PCa cells or PSAP-GFP infected cells were plated in 3.5-cm culture dish at 30% confluence and harvested at ∼60% confluence, fixed in 0.5% PFA for 1 h at 4°C, and then permeabilized in 70% cold ethanol at 4°C for 3 h. Cells were incubated in propidium iodide (PI) working solution (40 μg/ml, P4170; Sigma) at 37°C for 30 min and analyzed by FACS for cell-cycle profiles [11, 13]. To determine differential sensitivities of the PSA^−/lo^ and PSA^+^ PCa cell populations to various drug treatments, we performed FACS analysis using the Vybrant Apoptosis Kit (catalog #V23200; Molecular Probes, Invitrogen) according to the manufacturern’s instructions. The kit contained biotin-Annexin V, Alexa Fluor 350 (similar spectrum to DAPI) streptavidin, and PI. Briefly, LNCaP cells infected with the PSAP-GFP reporter construct were plated in 10-cm cell culture plates at 500, 000 cells per plate. Cells were treated with DMSO (vehicle control), etoposide (25 μM), paclitaxel (10 nM), CDSS plus bicalutamide (20 μM) or H_2_O_2_ (10 μM) for various time intervals with fresh drugs added every 1–2 days. Treated cells and controls were analyzed by FACS at 2–5 days after the initiation of treatments. Healthy live cells were identified as Annexin V dim and PI negative; apoptotic cells Annexin V positive and PI low/−; and necrotic cells Annexin V bright and PI bright.

### Quantitative RT-PCR (qRT-PCR)

Basic protocols for qRT-PCR have been described [12, 13]. In brief, qRT-PCR was performed using an ABI Prism 7900HT and the TaqMan system (ABI; Applied Biosystems, Foster City, CA; http://www.appliedbiosystems.com). The primers, probes, and assay conditions for other molecules were designed by ABI with the following information: PSA (Hs03063374_m1; assay number), AR (Hs00907244_m1), β-actin (Hs99999903_ml), CD44 (Hs00153304_m1), α2 integrin (Hs00158148_m1), and GAPDH (4326317E).

### Time-lapse videomicroscopy and estimate of cell-cycle transit time

Purified GFP^+^ and GFP^−^ LNCaP cells were plated on special glass-bottom dishes, placed on the incubator stage of Nikon Biostation Timelapse system [13], and maintained at 37°C, 5% CO2 and >95% humidity in the RPMI medium supplemented with 7% FBS. Phase and GFP images were collected continuously with a 20X objective lens at a 1-h interval for up to ∼1 week. Data analysis was performed using Nikon NIS-Elements software. Several dozens of recorded GFP^+^ and GFP^−^ images were analyzed in detail for cell-cycle transit times using the first cell division as the starting point.

### Correlating *PSA* mRNA levels with clinical outcomes of PCa patients in *Oncomine*

A total of 27 *Oncomine* PCa data sets containing *KLK3* mRNA expression data (Supplementary Table 2) were analyzed in detail for correlations with available patient parameters including survival, recurrence, metastasis, Gleason score, serum PSA levels, and LN status. Significance of *PSA* mRNA between conditions was determined by Student’s *t*-test, and *P* values less than 0.05 were considered statistically significant. Box plot data presentations and statistical analyses were generated using program R. We also performed survival analysis and generated Kaplan-Meier survival plots using the survival package in R. Briefly, we first input the individual normalized gene expression data from patients with both survival time and survival status from *Oncomine* and ranked the data according to *PSA* mRNA expression. We then assigned the samples with rankings from the first quartile to the third quartile into two groups and compared the *P*-value between these two groups along with different cutoffs. Finally, we set the ultimate cutoff with the smallest *P*-value and plotted a Kaplan-Meier survival curve.

### Determination and GO analysis of genes commonly upregulated in both LNCaP and LAPC9 cDNA microarrays

We previously performed cDNA microarrays in PSA^−/lo^ versus PSA^+^ LNCaP and LAPC9 cells [13] and all microarray data have been deposited in the NCBI GEO database (www.ncbi.nlm.nih.gov/geo/query) under the accession number GSE15411 and GSE30114. To determine commonly changed genes, we first selected the genes (by Agilent’s Probe ID) either up-regulated or down-regulated using a 1.4FC cutoff from both LAPC9 and LNCaP gene lists (from raw data file) and used these genes for Venn diagram analysis. This analysis identified 3,949 and 3,338 upregulated probe ID’s in PSA^−/lo^ LAPC9 and LNCaP cells, respectively, over the corresponding PSA^+^ cells, of which 570 probe ID’s were shared (see Supplementary Figure 4B). The probe ID’s were then converted into official gene names (symbols) using the ID conversion tool available in DAVID, which identified a total 337 genes commonly upregulated in both LNCaP and LAPC9 PSA^−/lo^ populations. We performed Gene Ontology (GO) analysis of 337 commonly upregulated genes using functional annotation tool in DAVID.

### ChIP and re-ChIP assays

To determine if PSA^−/lo^ and PSA^+^ tumor cells differed in chromatin composition and stem-cell associated bivalent domains, ChIP and re-ChIP assays [30] were performed using chromatin from prospectively purified PSA^−/lo^ and PSA^+^ LAPC9 and LNCaP cells. Freshly sorted PSA^−/lo^ and PSA^+^ cells were fixed for 15 min at RT by addition of freshly prepared neutral buffered formalin to their respective media at a final concentration of 0.75%, with gentle rocking. Formalin was quenched by the addition of 2 M glycine to a final concentration of 0.125 M and incubation for 5 min at RT with gentle rocking. Cells were then pelleted and resuspended in 750 μl of ChIP lysis buffer (50 mM HEPES-KOH, 140 mM NaCl, 1 mM EDTA, pH 8.0, 1% Triton X100, 0.1% sodium deoxycholate, and Roche complete protease inhibitor cocktail) for every million cells, and sonicated on ice until the majority of the chromatin had been sheared into 500–1000 bp fragments. 50 μl was removed for use as input, and the remaining chromatin was divided into aliquots for immunoprecipitation (IP) and diluted 10x with dilution buffer (1% Triton X-100, 2 mM EDTA, 150 mM NaCl, 20 mM Tris-HCl, pH 8.0, and Roche protease inhibitor cocktail).

In all immunoprecipitations, 5 μg of primary antibody or control IgG was used for the initial ChIP as well as re-ChIPs performed. Antibodies used in the initial ChIP were ChIP-grade rabbit control IgG, anti-Histone H3, anti-Histone H3K4 (trimethyl), and anti-Histone H3K27 (trimethyl). For the re-ChIP, a mouse monoclonal antibody raised against histone H3K27 (trimethyl) was used. For each ChIP antibody, 60 μl of Invitrogen Dynal beads were washed and blocked in PBS-0.1% BSA. Half of the beads were incubated overnight with the chromatin samples without antibody, as a pre-clearing step to reduce background due to non-specific chromatin interaction with the beads. Beads used for pre-clearing were then discarded. The other half of the beads were incubated with antibodies for ChIP while pre-clearing was ongoing. The following day, IP’s were performed by combining pre-cleared chromatin samples with antibody-bead complexes and incubating overnight at 4°C with gentle rocking. The next day, the beads were washed 3 × 5 min. with ChIP wash buffer (0.1% SDS 1% Triton X-100 2 mM EDTA 150 mM NaCl 20 mM Tris-HCl, pH8) and the bound chromatin eluted by incubating the beads with 450 μl of 100 mM sodium bicarbonate, 20 mM DTT for 15 min. at RT. 50 μl of each ChIP was removed for use as input before incubating the IP eluate with the re-ChIP antibody. Re-ChIPs were performed by incubating the eluted chromatin from the first round of ChIP with the H3K27 (trimethyl) mAb/bead mixture overnight at 4°C with gentle rocking. The re-ChIP’ed chromatin was washed 3× for 5 min. in ChIP wash buffer and then eluted by incubation with 450 μl of 100 mM sodium bicarbonate, 1% SDS for 15 min at RT. Prior to use in PCR reactions to detect the immunoprecipitated DNA, all samples and inputs were subjected to cross-link reversal by addition of 5 μl of 20 mg/ml proteinase K and incubation at 60°C overnight. Protein was removed from ChIP samples and inputs by phenol-chloroform extraction and alcohol precipitation, and inputs were resuspended in 100 μl of ddH2O while samples were resuspended in 30 μl ddH2O. PCR was typically performed for 33 cycles with 1 min. for extension and 30 seconds for denaturing and annealing steps with a 5-minute final extension. Promega GoTaq 2X master mix was used for all reactions. ChIP primers were targeted to sequences approximately 1000 bp from the TSS (transcription start site) and were as follows:

**Table:** 

AR	Forward: 5′-GGGTGATTTTGCCTTTG AGA-3′
	Reverse: 5′-GGCTTTGGAGAAACAA GTGC-3′
ASCL1	Forward: 5′-TTCACCCCAAGTCTTTC CAC-3′
	Reverse 5′-ACTAAGGCTGCGCTCTC TTG-3′
BCL2	Forward: 5′-GTCTGGGAATCGATCTGGAA-3′
	Reverse: 5′-GCGGAACACTTGATTCT GGT-3′
CD61	Forward: 5′-CACACACACATGCAAA CGAG-3′
	Reverse: 5′-CACCCTCCCAAACACT AGGA-3′
CDH2	Forward: 5′-GCGGGAGGAATAGGAG AGG-3′
	Reverse 5′-ATGTGGAGGTGGAAGTG GAG-3′
FGF5	Forward: 5′-CAATCATCCTCCCCAG AAGA-3′
	Reverse: 5′-TTGCATGCTTGGAATG TTTC-3′
NKX3-1	Forward: 5′-ACTCACTGCAGCCTCG ATTT-3′
	Reverse: 5′-CCCGTTGCACAGGTAG TTTT-3′
PPP2R4	Forward: 5′-CCTGTCCCCACATGTC TTCT-3′
	Reverse: 5′-CCTCTCGCCTTTCACT CTTG-3′

Quantification of relative binding in ChIP assays was performed using NIH ImageJ software (http://stanxterm.aecom.yu.edu/wiki/index.php?page=Using_ImageJ), and each gene promoter was analyzed in 3 independent immunoprecipitations. Arbitrary optical density values obtained through ImageJ were scaled for each ChIP by setting the pan-Histone H3 band to 1.

### Statistics

In general, unpaired two-tailed Student’s *t*-test was used to compare differences in cell numbers, cell-cycle transit time, cloning and sphere-forming efficiency, tumor weights, and many other parameters. Fisher’s Exact Test and χ^2^ test were used to compare incidence and latency. Log-Rank test was employed to analyze the survival curves and ANOVA (F-test) was used to compare multiple groups. In all these analyses, a *P* < 0.05 was considered statistically significant.

## SUPPLEMENTARY FIGURES AND TABLES




