# Supplementary material for: Systematic dissection of phenotypic, functional, and tumorigenic heterogeneity of human prostate cancer cells
Source: Oncotarget. 2015 Jun 24;6(27):23959–86. doi: 10.18632/oncotarget.4260 (PMC4695164; doi:10.18632/oncotarget.4260)
Supplement: Supplementary file 1 [file oncotarget-06-23959-s001.pdf]

SUPPLEMENTARY FIGURES AND TABLES

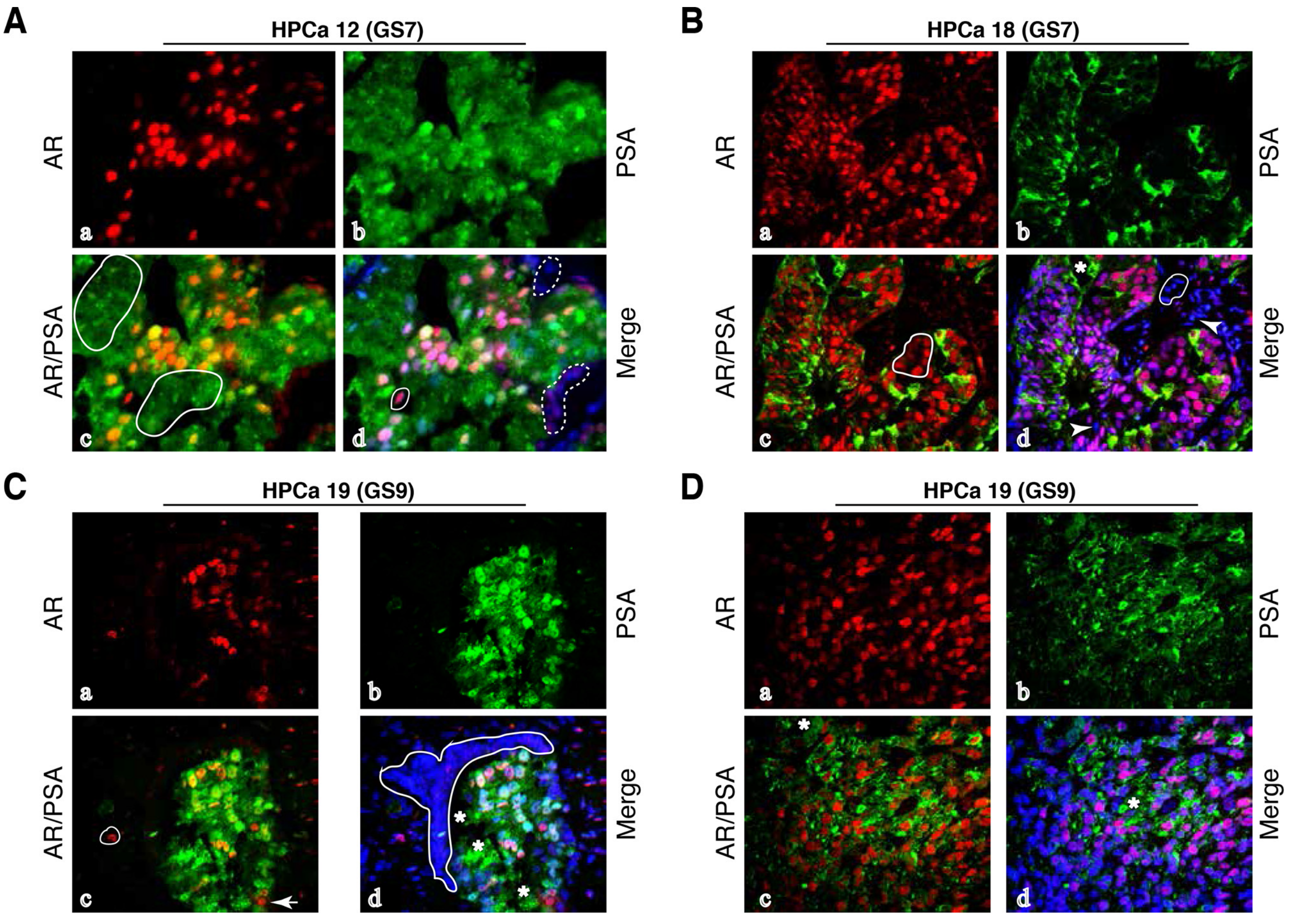

Supplementary Figure 1: Discordant PSA and AR protein expression and subtypes of PCa cells in untreated HPCa. A. Representative immunofluorescence images (×400) illustrating 4 subpopulations of PCa cells in HPCa 12. Although most cells are AR^+PSA^+ PCa cells marked by red nuclei and green cytoplasm, AR^-PSA^+ cells can be seen by green staining alone (panel c, white circled areas), AR^+PSA^- cells by red staining alone (panel d, one white circled area), and AR^-PSA^- cells by being negative or low for both red and green staining (panel d, dashed white circled areas). **B.** Representative immunofluorescence images (×400) illustrating 4 subpopulations of PCa cells in HPCa 18. AR^+PSA^- cells are identified by red staining alone (panel c, white circled area), AR^-PSA^+ cells by green staining alone (panel d, white asterisk), and AR^-PSA^- cells by being negative or low for both colors (panel d, white circled area and white arrowheads). **C–D.** Representative immunofluorescence images (×400) illustrating 4 subpopulations of PCa cells in HPCa 19. AR^+PSA^- cells are identified by red staining alone (Cc, white circled area and white arrow), AR^-PSA^+ cells by green staining alone (Cd and Dc-d, white asterisks), and AR^-PSA^- cells by being negative (or low staining) for both colors (Cd, white circled area).

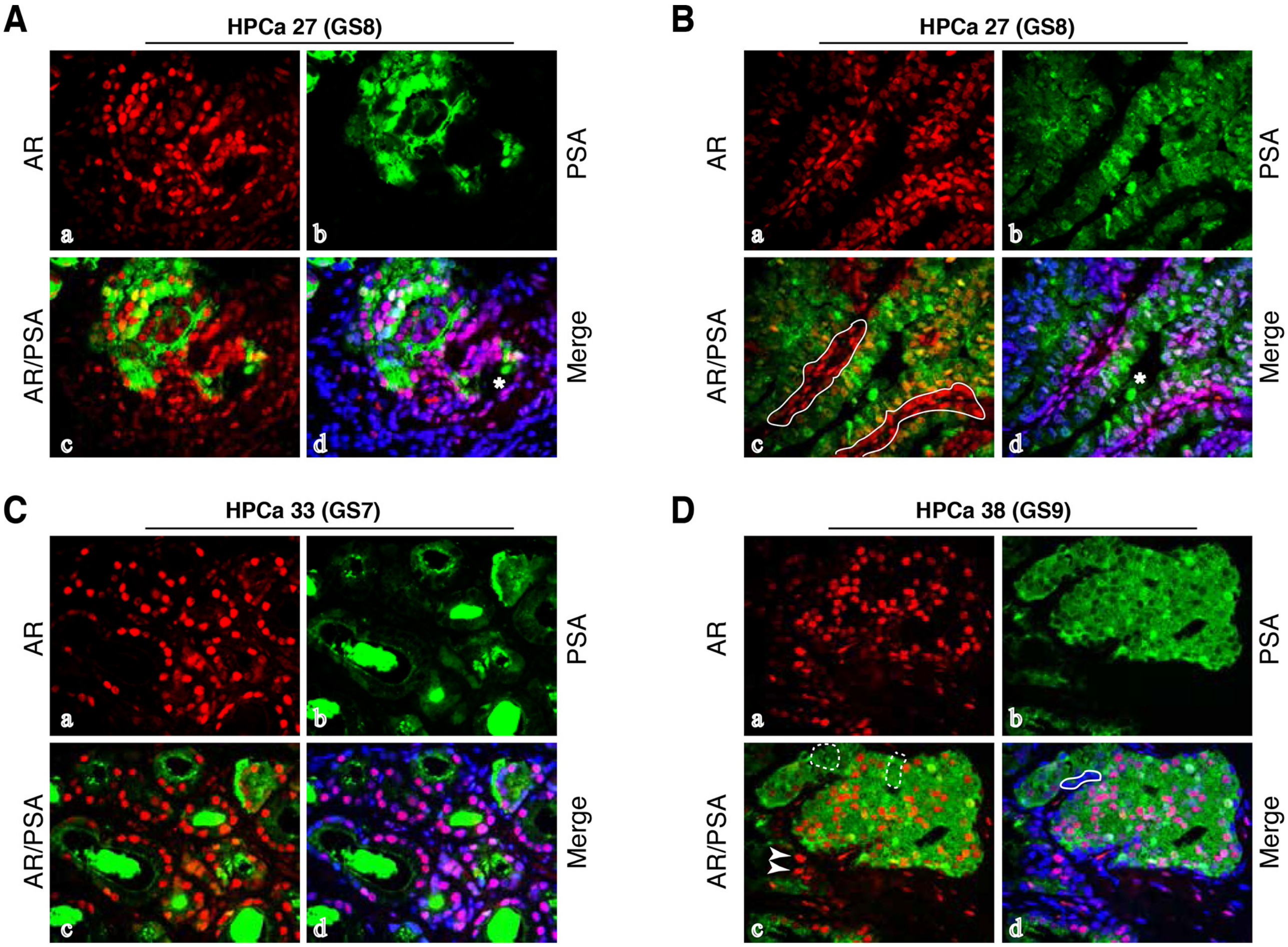

Supplementary Figure 2: Discordant PSA and AR protein expression and subtypes of PCa cells in untreated HPCa. A–B. Representative immunofluorescence images (×400) illustrating 4 subpopulations of PCa cells in HPCa 27. $AR^{+}PSA^{-}$ cells are quite abundant in this sample (Ac, red alone cells; Bc, white circled area). Rare $AR^{-}PSA^{+}$ cells can be seen by green staining alone (Bd, white asterisk). $AR^{-}PSA^{-}$ cells are negative or low for both red and green staining (Ad and Bd, cells positive for DAPI only). **C.** Representative immunofluorescence images (×400) of AR and PSA staining in HPCa 33. This Gleason 7 tumor manifests numerous small glands in which PSA^{+} secretions can be observed in the lumen. There are many $AR^{+}PSA^{-}$ cells (Cc, cells with red nuclei alone) and $AR^{-}PSA^{-}$ cells (Cd, cells low or negative for both red and green signals). **D.** Representative immunofluorescence images (×400) illustrating 4 subpopulations of PCa cells in HPCa 38. $AR^{+}PSA^{+}$ cells are marked by red nuclei and green cytoplasm (panel c), $AR^{+}PSA^{-}$ cells by red staining alone (panel c, white arrowheads), $AR^{-}PSA^{+}$ cells by green staining alone (panel c, white dashed circled cells), and $AR^{-}PSA^{-}$ cells by being negative or low for both red and green staining (panel d, white circled area and many single DAPI-positive cells).

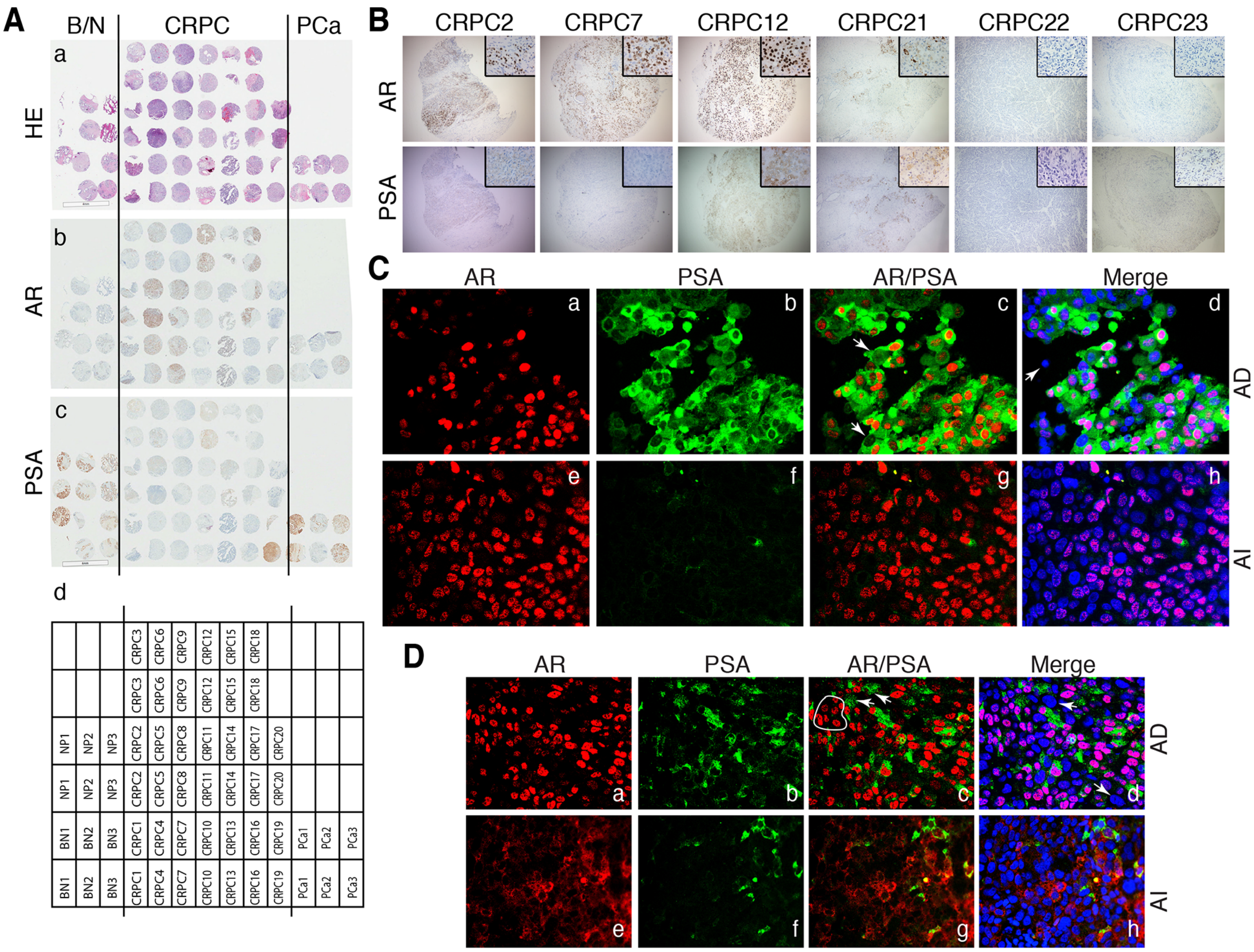

Supplementary Figure 3: Discordant PSA and AR protein expression, 4 subtypes of PCa cells, and increase of PSA$^{-/lo}$ PCa cells in CRPC. **A.** AR and protein expression in 20 CRPC patient samples on a TMA. Shown on top are HE (a) and AR (b) and PSA (c) IHC images of the TMA and below the TMA grid (d). The 20 CRPC samples are in the middle (demarcated by two vertical lines whereas several benign/normal prostate and PCa samples are shown on the left and right, respectively. All samples were cut in duplicate. NP, normal prostate (i.e., no cancer); BN, benign prostate from patients with PCa; PCa, hormone-naïve prostate cancer. CRPC1–13, PCa patients treated with castration (mostly bicalutamide) and eventually failed after months to years; CRPC14–17, patients failed both radiation and hormonal therapies; CRPC18, the patient failed radiation and cryotherapy; CRPC19, the patient with advanced PCa treated with Lupron for 2 weeks; CRPC20, the patient received 4 months of Lupron treatment plus 2 months of Casodex. **B.** IHC analysis of AR and PSA in the TMA samples. Shown are 7 CRPC samples illustrating prominent loss of PSA, heterogeneous expression of AR, and discordant AR and PSA expression (insets: 400×). CRPC21 – CRPC23 were 3 separate patient CRPC samples not included in the TMA. **C.** Double immunofluorescence staining of AR and PSA in AD vs. AI LNCaP xenograft tumors. Arrows indicate AR^{-}PSA^{+} (c) or AR^{-}PSA$^{-/lo}$ (d) cells. Note significantly increased PSA$^{-/lo}$ LNCaP cells in the AI tumor (f). Shown are representative confocal images (original magnification; ×400). **D.** Double immunofluorescence staining of AR and PSA in AD vs. AI LAPC4 xenograft tumors. In panel c, the white circle indicates several AR^{+}PSA$^{-/lo}$ cells and arrows indicate AR^{-}PSA^{+} cells. In panel d, arrows point to AR^{-}PSA$^{-/lo}$ cells. Note significantly increased PSA$^{-/lo}$ LAPC4 cells in the AI tumor (f). Shown are representative confocal images (original magnification; ×400).

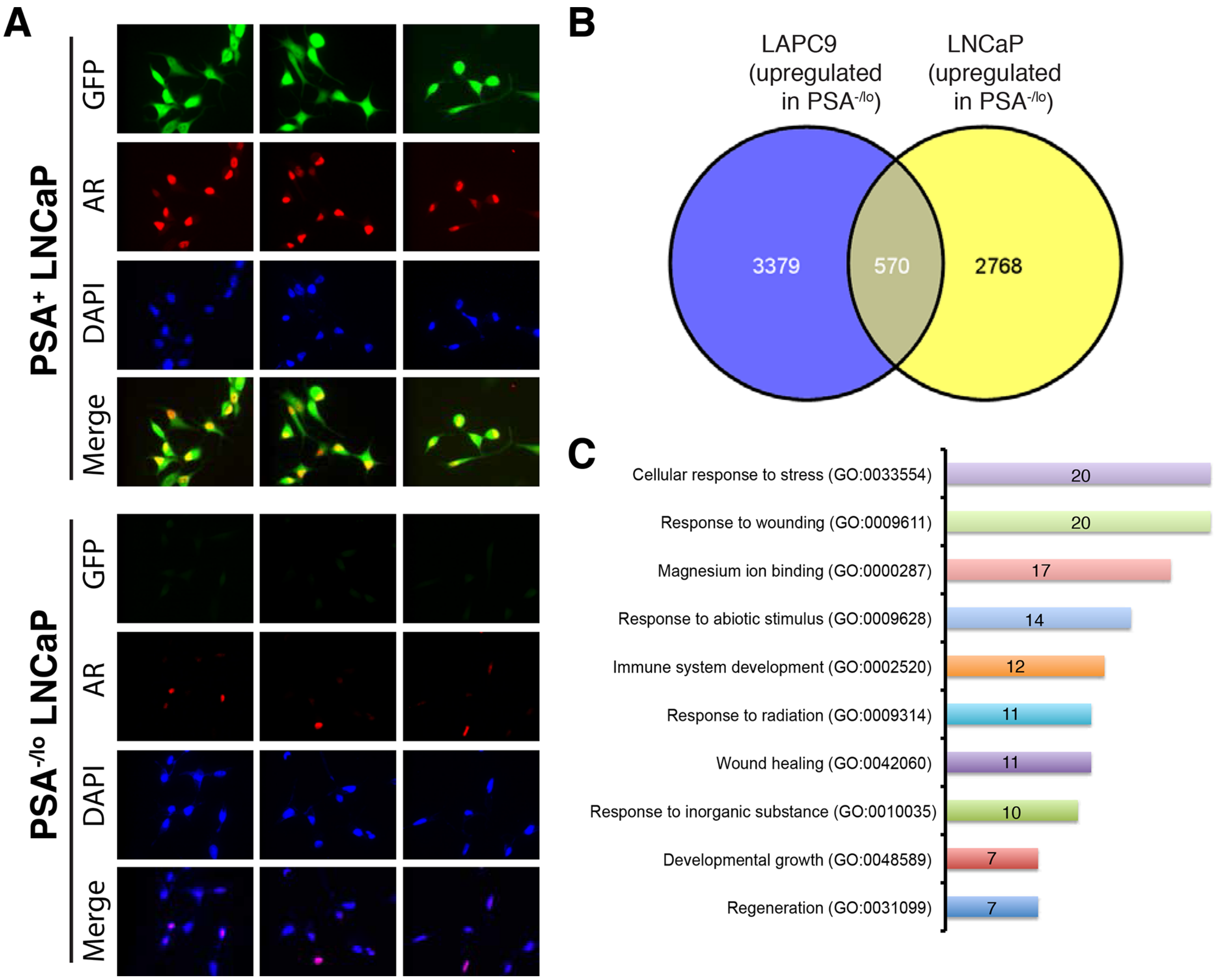

Supplementary Figure 4: PSA$^{-/lo}$ PCa cells are heterogeneous in AR expression and overexpress anti-stress genes. A. GFP^{+} (i.e., PSA^{+}) LNCaP cells express high levels of nuclear AR whereas GFP$^{-/lo}$ (PSA$^{-/lo}$) LNCaP cells are negative or weakly positive for nuclear AR. Shown are 3 representative fields (×400) of purified GFP^{+} (top) and GFP^{-} (bottom) LNCaP cells stained for AR and DAPI, representing 3 independent sorts. **B–C.** Commonly upregulated genes in PSA$^{-/lo}$ LAPC9 and LNCaP cells. Shown in A is Venn diagram presentation of commonly upregulated genes and in B is GO analysis performed using DAVID. The EASE score (a modified Fishcer's Exact test) cut off was set to stringent *p*-value of 0.005 and the top 10 GO categories appeared under GO term 'Biological Process' are presented in the graph. The GO terms and the GO term numbers are presented on the Y-axis and the gene counts are presented inside the bars. The genes for 'Cellular response to stress' are LPO, XRCC2, GEN1, SNCA, SMC5, BRCA2, LIG4, TP73, DCLRE1C, FANCD2, BCL2, GPX3, TPO, MAPK8, TLK1, TLK2, POLQ, MAP2K7, WDR33, and FGD4. The genes for 'Response to wounding' are CCL2, GATM, FUT10, IGF1, ABHD2, GPR68, TP73, PLAA, SLC11A1, F5, FCN3, SAA1, BCL2, IL1RAP, PDGFRA, SPRR3, SERPINB2, SOX15, BMPR1B, and PLAU. The genes for 'Response to abiotic stimulus' are KCNMA1, TG, PTPRC, CCL2, XRCC2, TGFBR1, BRCA2, LIG4, SNAI2, DCLRE1C, FANCD2, BCL2, MAPK8, and RHO. The genes for 'Immune system development' are PTPRC, HSPD1P6, SWAP70, TGFBR1, FUT10, BRCA2, HSPD1P1, LIG4, HSPD1P5, HSPD1P4, DCLRE1C, TAL1, SNRK, BCL2, ZAP70, and HSPD1. The genes for 'Response to radiation' are DCLRE1C, PTPRC, XRCC2, CCL2, FANCD2, BCL2, BRCA2, MAPK8, LIG4, SNAI2, and RHO. The genes for 'Wound healing' are SLC11A1, F5, GATM, SAA1, FUT10, PDGFRA, SPRR3, SERPINB2, SOX15, IGF1, and PLAU.

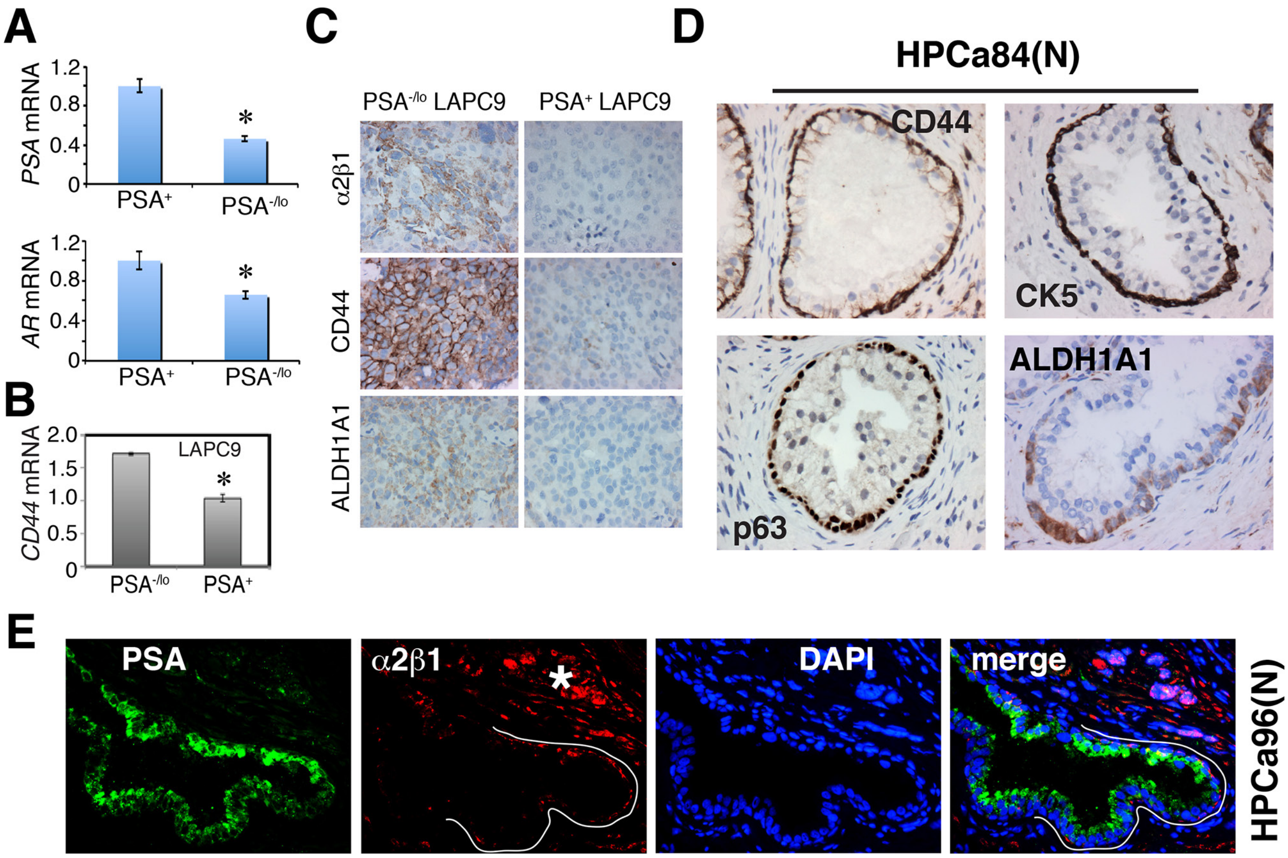

Supplementary Figure 5: Relationship between PSA$^{-/lo}$ PCa cells and other tumorigenic subsets in xenograft tumors and HPCa samples. A. PSA$^{-/lo}$ LAPC9 cells express lower levels of *PSA* (top) and *AR* (bottom) mRNAs than the corresponding PSA^{+} cells. The two populations of LAPC9 cells were freshly purified out from maintenance AD LAPC9 reporter tumors and used in qRT-PCR of *AR* and *PSA*. Shown are the qPCR results (n = 3 independent experiments). *P < 0.001. **B.** PSA$^{-/lo}$ LAPC9 cells express higher levels of *CD44* mRNAs than the corresponding PSA^{+} cells. Shown are the qPCR results (n = 3). *P < 0.01. **C.** PSA$^{-/lo}$ LAPC9 tumors serially passaged in intact male NOD/SCID mice have higher % of α2β1^{+}, CD44^{+}, and ALDH1A1^{+} cells compared to corresponding PSA^{+} LAPC9 cell-derived tumors. Shown are representative IHC images from the 3^{0} generation tumors. Original magnification, ×400. **D–E.** Marker expression in normal (benign) prostate tissues. Shown in D are basal expression of CK5 and p63 as well as CD44 and ALDH1A1 in benign prostatic glands in HPCa84(N). Shown in E are dual IF images in HPCa96(N) of PSA, which is expressed in the luminal layer, and α2β1, which is expressed in a fraction of basal cells (demarcated by white line; *indicates α2β1 expression in some stromal cells). Similar results were obtained in HPCa93(N) (not shown). Note that although CK5, p63, and CD44 are expressed in virtually all basal cells, ALDH1A1 and α2β1 are expressed in only a fraction of basal cells (all images: ×200).

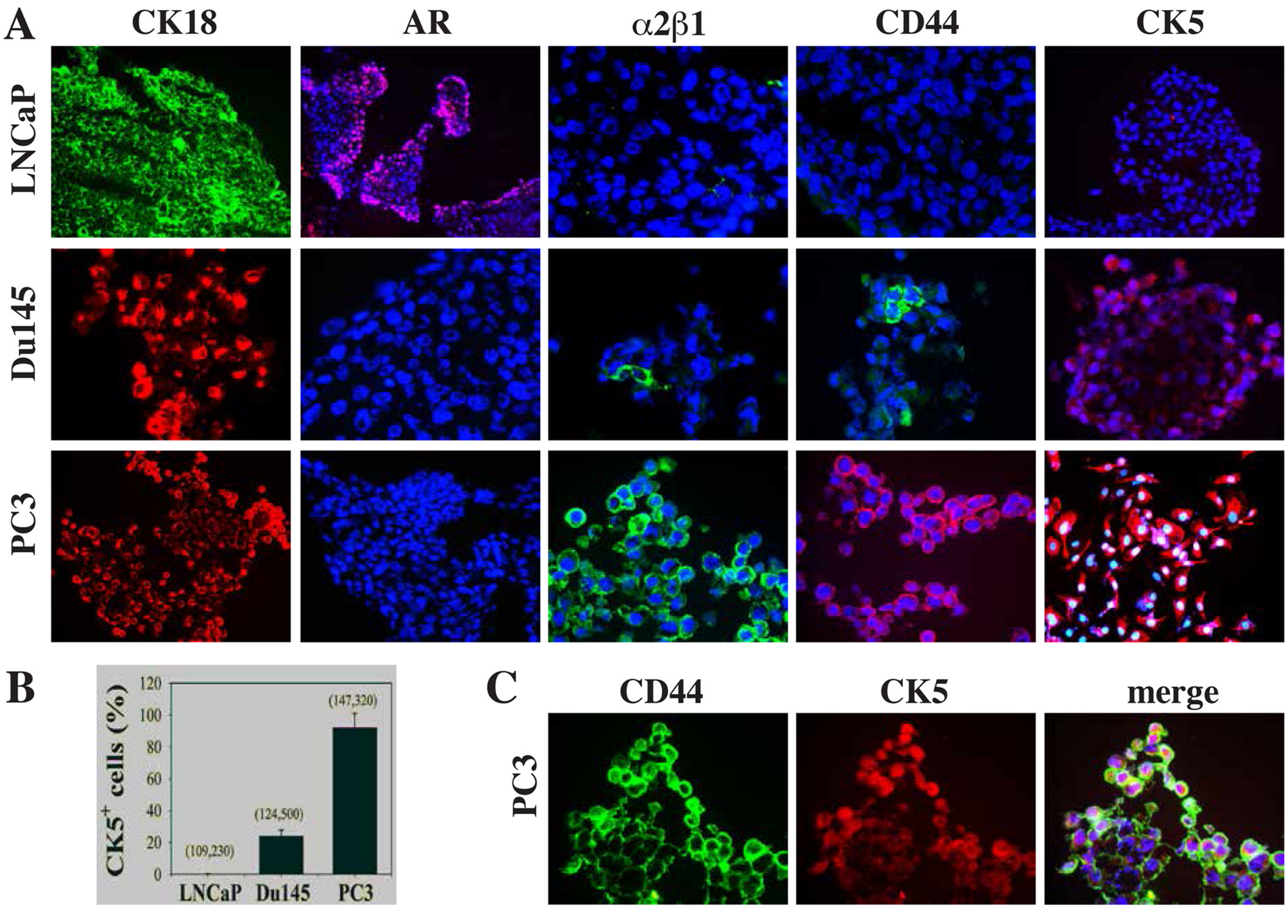

Supplementary Figure 6: Marker expression in 3 PCa cell lines. A. LNCaP, Du145, and PC3 cell spheres/spheroids on cryosections were used in immunostaining for the molecules indicated. Note that all three PCa cell lines expressed CK18 but only LNCaP cells stained positive for AR. **B.** The % of CK5+ cells was quantified in LNCaP, Du145 or PC3 spheres/spheroids. The total numbers of cells counted were indicated parentheses. **C.** In PC3 cell spheroids, CD44 staining overlapped with CK5 staining. Original magnifications: ×200.

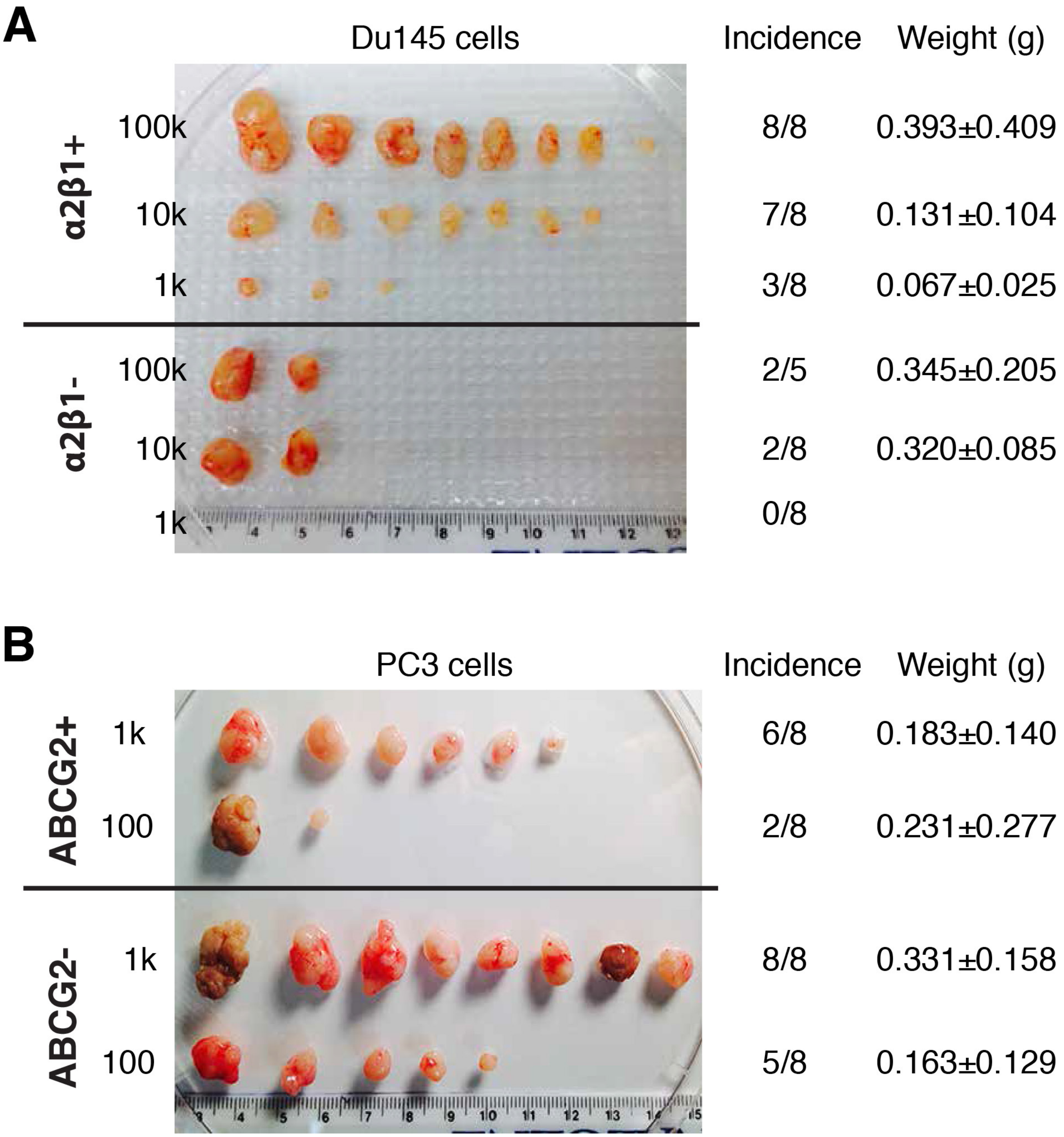

Supplementary Figure 7: LDA tumorigenic assays in subpopulations of Du145 (A) and PC3 (B) cells. (A) Integrin $\alpha2\beta1^+$ and $\alpha2\beta1^-$ Du145 cells were FACS-purified from log-phase cultures and implanted, at the indicated numbers, subcutaneously, in 50% Matrigel in female NOD/SCID mice. The experiment was terminated 74 d after implantation. (B) $ABCG2^+$ and $ABCG2^-$ PC3 cells were purified from cultures and implanted, at the indicated numbers, subcutaneously, in 50% Matrigel in female NOD/SCID mice. The experiment was terminated 57 d after implantation. Not shown were tumor images of the 10k injections, which were harvested 47 d after implantation. The 10k $ABCG2^+$ (incidence 5/5) and $ABCG2^-$ (incidence 7/8) PC3 tumors were 0.316 ± 0.191 and 0.240 ± 0.138 g, respectively. For both A and B, tumor incidence (also see Table 1) and mean tumor weight (no statistical significance) are indicated.

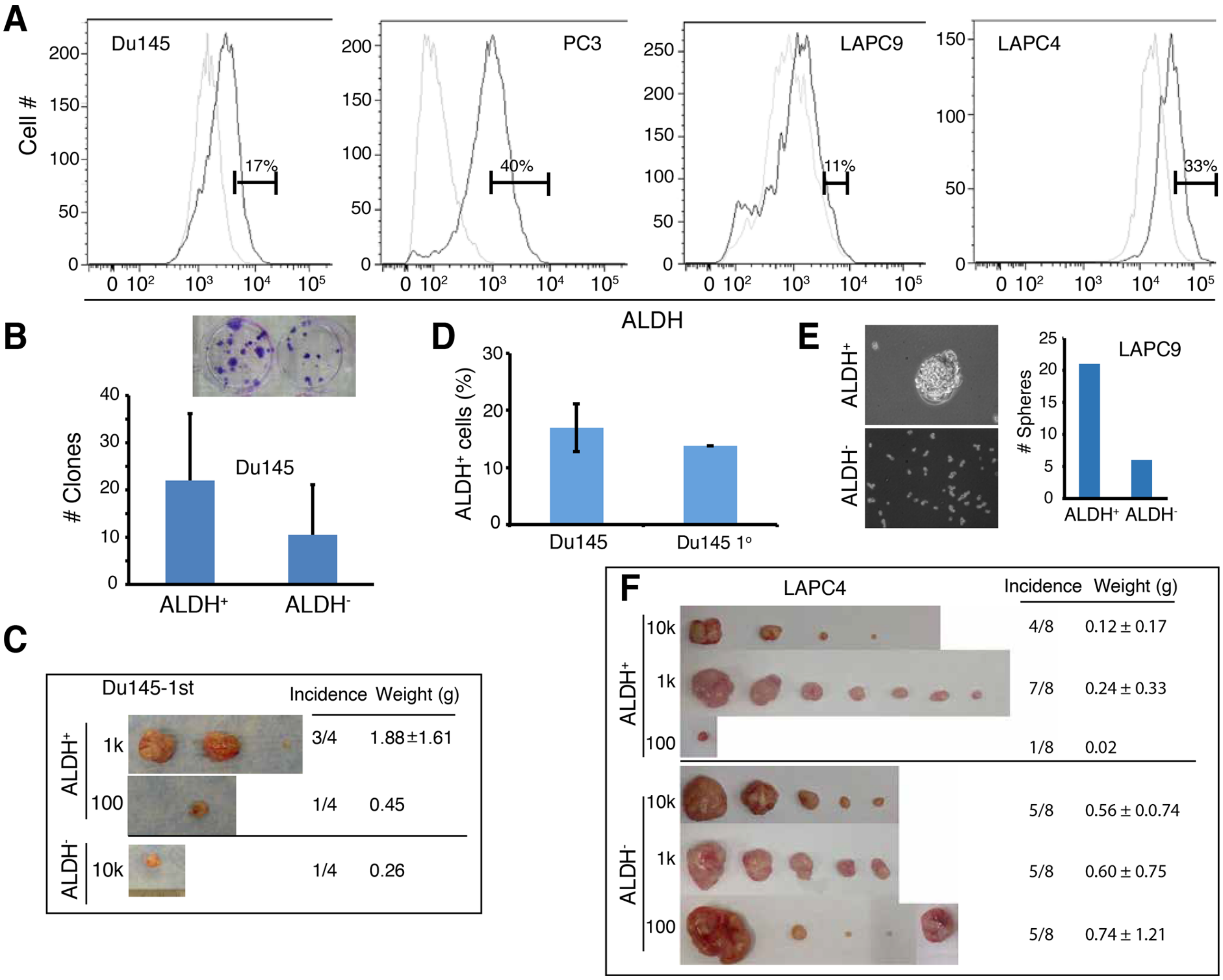

Supplementary Figure 8: The ALDH⁺ PCa cell subpopulations are enriched in tumor-initiating cells. A. Shown are the representative FACS profiles of ALDH activities in four PCa models assessed by the ALDHFLOUR assays. **B.** $ALDH^+$ and $ALDH^-$ cells were sorted from cultured Du145 cells and plated in 6-well dishes at clonal density (100 cell per well). 15 days after plating, holoclones were counted. Shown are the clone number in bar graph (mean ± S.D) and representative clone images (inset). **C.** Purified $ALDH^+$ and $ALDH^-$ Du145 cells were subcutaneously implanted in NOD/SCID mice at the indicated cell doses. Tumor images, incidence and weights (mean ± S.D) are shown. **D.** The percentage of $ALDH^+$ Du145 cells in parental Du145 cell culture and 1° tumors derived from $ALDH^+$ cells. **E.** $ALDH^+$ and $ALDH^-$ LAPC9 cells were purified and cultured in anchorage-independent conditions in IMDM-15% FBS. 24 d later, spheres were counted and photographed. Insets were representative images of spheres derived from $ALDH^+$ and $ALDH^-$ LAPC9 cells, respectively. **F.** Sorted $ALDH^+$ and $ALDH^-$ cells from LAPC4 xenograft tumors were subcutaneously injected in intact male mice. Shown are tumor weights (mean ± S.D) and incidence of tumors derived from indicated cell doses (also see Table 2).

A

Tumorigenicity of the orthotopically implanted LAPC9 cells

Cell type (number)[a]	Incidence[b]	Term. time (d)[c]	Weight (g)[d]
$CD44^+$			
1,000	2/5 (40%)	65	0.1
10,000	5/9 (56%)*	65	1.2
$CD44^-$			
1,000	0/8	90	
10,000	1/12 (8.3%)	76	0.05

[a]LAPC9 xenograft tumors were digested into single-cell suspension and used in sorting for $CD44^+$ and $CD44^-$ cells. Either 1,000 or 10,000 cells were implanted in 50% Matrigel into the DP of the intact male NOD/SCID mice.
[b]Refers to tumors developed/total number of implants. The % incidence was indicated. *$P < 0.001$ compared to the incidence of $CD44^-$ cells.
[c]Time (in days) from tumor implantation to termination (term.).
[d]The mean tumor weight in grams.

B

2° transplantation (LAPC9)

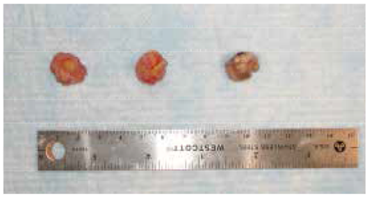

$CD44^+$ (3/6)

$CD44^-$ (0/4)

C

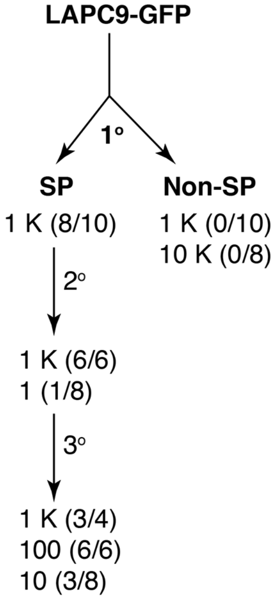

D

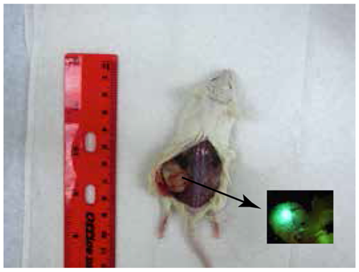

Supplementary Figure 9: Tumorigenic properties of prospectively purified $CD44^+$ and SP LAPC9 cells. A. LDA transplantation assays in $CD44^+$ and $CD44^–$ LAPC9 cells implanted in the DP of male NOD/SCID mice. Experimental details and outcome are indicated in the Table legend. This represents an independent experiment from what was shown in Table 2. **B.** $CD44^+$ and $CD44^–$ LAPC9 cells were purified, resepctively, from the $CD44^+$ and $CD44^–$ cell-derived 1° tumors (in A) and implanted in the DP of male NOD/SCID mice (1,000 cells/injection). The experiment was terminated 3 months after tumor cell injections. **C–D.** An independent tumor transplantation assay in SP and non-SP LAPC9 cells. The SP and non-SP cells were purified out from a LAPC9-GFP xenograft tumor and indicated numbers of cells were implanted subcutaneously in male NOD/SCID mice. The SP cells were subsequently purified out from regenerated tumors for 2° and 3° transplantations. Tumor incidence is indicated in parentheses. Shown in D is the image (inset, GFP) of the tumor derived from a single SP cell injection in 2° transplantations (C).

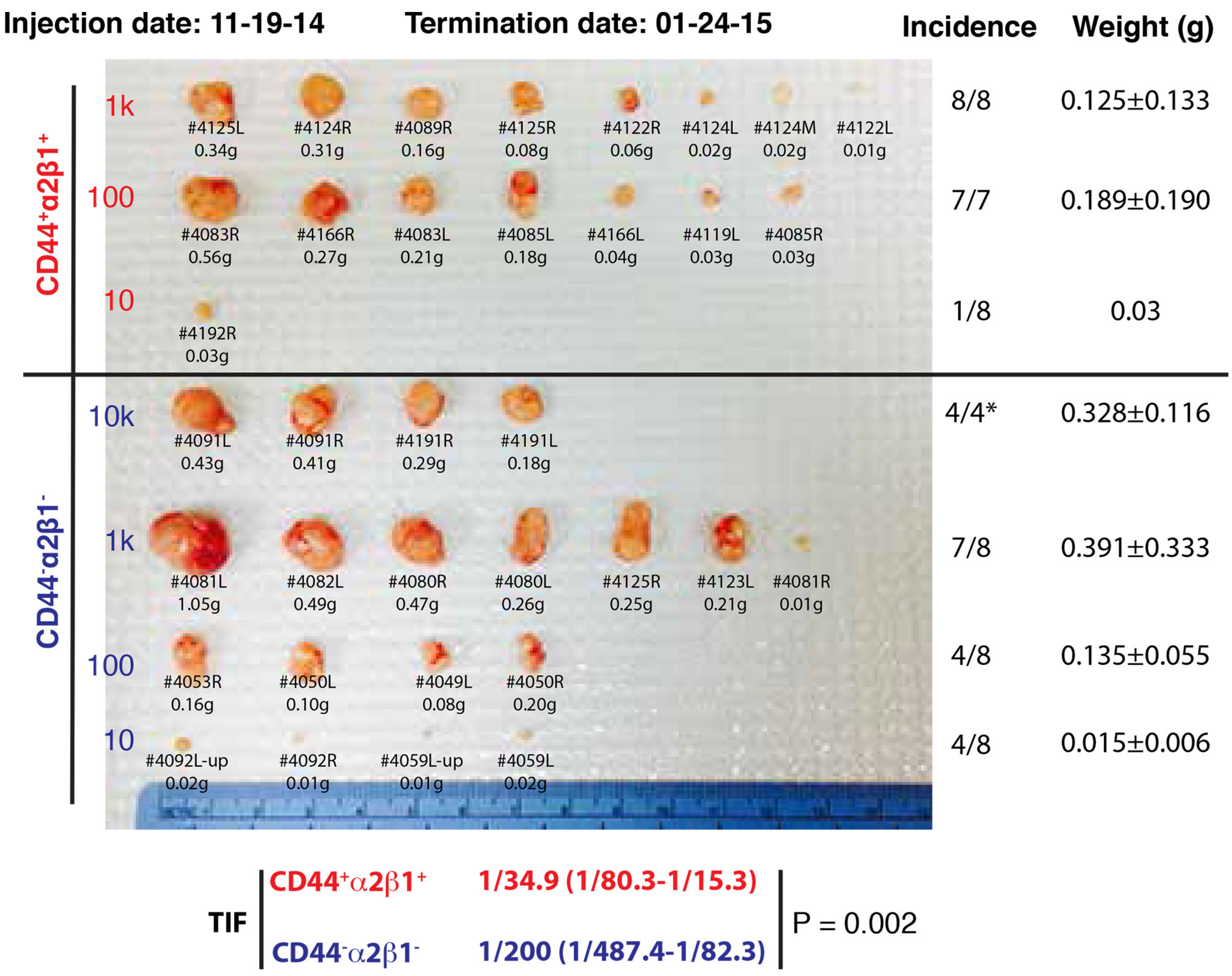

Supplementary Figure 10: CD44$^+$α2β1$^+$ LAPC4 cells are more tumorigenic than CD44$^-$α2β1$^-$ cells. CD44$^+$α2β1$^+$ and CD44$^-$ α2β1$^-$ LAPC4 cells were FACS-purified from maintenance xenograft tumors and implanted, at the indicated numbers, subcutaneously, in 50% Matrigel in female NOD/SCID mice. The experiment was terminated 66 d after implantation. Not shown were tumor images of the 4 10k injections of CD44$^-$α2β1$^-$ LAPC4 cells, which were harvested one week earlier due to IACCUC regulation (lower panel, indicated by an asterisk). Shown on the right are tumor incidence and mean tumor weight. Shown below is the calculated TIF and *P* value (also see Table 2).

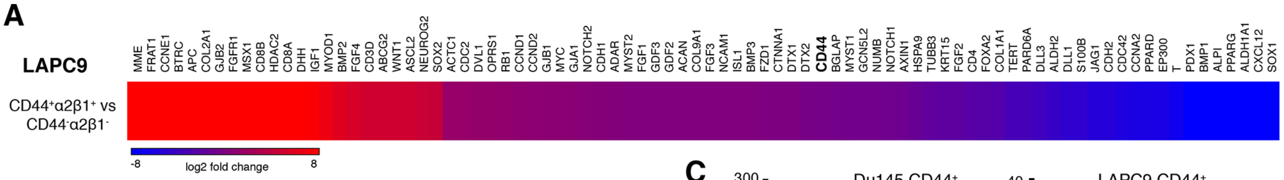

B Relationship between phenotypic markers based on qPCR Superarray results

Cell population	CD44	ABCG2	ALDH1A1	ALDH2
**Du145**				
CD44+	++++	+++	++	+++
α2β1+	++++	↓	++++	++
**LAPC9**				
CD44+	++++	+++	+	-
CD44+α2β1+	+	+++	↓	↓

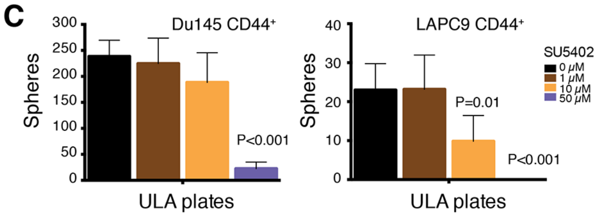

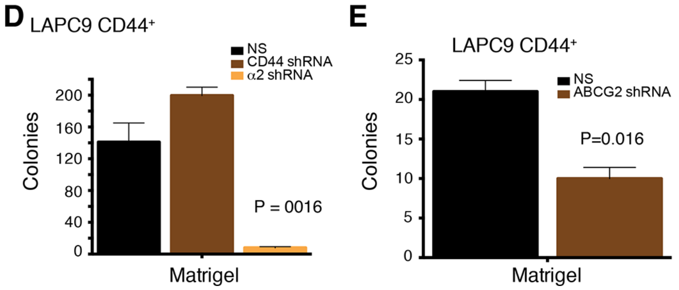

Supplementary Figure 11: Gene expression and functional studies in tumorigenic PCa cell populations. A. Expression of 84 stem cell-related genes in the CD44$^+$α2β1$^+$ LAPC9 cells. Relative expression levels were normalized to 5 internal controls (B2M, HPRT1, RPL13A, GAPDH and ACTB). Scale bars depict fold changes (in log 2 ratio), centered at 0. Interestingly, CD44 mRNA (highlighted in bold) was not among the highest expressed genes. **B.** Relative mRNA levels of CD44, ABCG2, ALDH1A1, and ALDH2 in the indicated Du145 and LAPC9 cell populations. ++++ to +, strongest to moderately increased; –, no significant enrichment in marker-positive cells;↓, decreased in marker positive population. **C.** The FGFR inhibitor SU5402 dose-dependently inhibited the sphere formation of CD44$^+$ PCa cells. FACS-purified Du145 and LAPC9 CD44$^+$ cells were plated in 6-well ULA plates (2,000 cells/well) and treated with SU5402 at 1, 10, and 50 μM. Spheres were enumerated 2 weeks after plating. *P* values were indicated where appropriate (Student's *t*-test). **D–E.** Knocking down integrin α2 and ABCG2 but not CD44 inhibited clonogenic activity of CD44$^+$ LAPC9 cells. FACS-purified LAPC9 CD44$^+$ cells were infected with non-silencing (NS) or 3 target silencing lentiviral vectors (MOI 20) and plated in Matrigel-coated 12-well plates (3,000 cells/well). Spheres were enumerated 2 weeks after plating. *P* values were indicated where appropriate (Student's *t*-test).

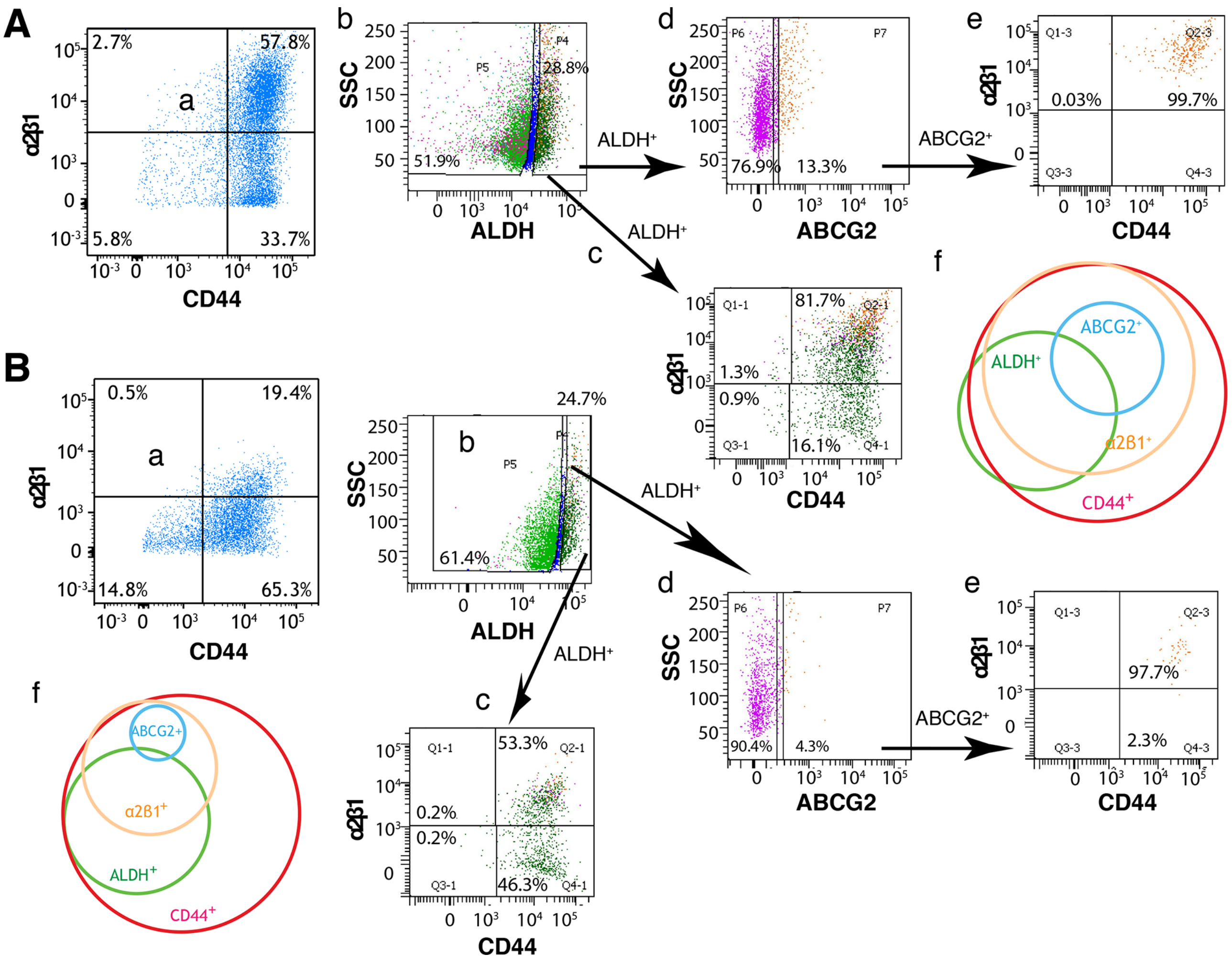

Supplementary Figure S12: Phenotypic relationship between different tumorigenic subpopulations of Du145 (A) and LAPC9 (B) cells. (A) Cultured Du145 cells were used in flow analysis of CD44 and integrin α2β1 expression, in which $CD44^+\alpha 2\beta^+$ cells constituted 57.8% of the total (a) whereas the $ALDH^+$ populations represented ~29% (b). Approximately 82% of the $ALDH^+$ population was $CD44^+\alpha 2\beta^+$ (c) whereas only ~13% of $ALDH^+$ cells were $ABCG2^+$ (d). ~98% (i.e., 81.7% + 16.1%) of $ALDH^+$ cells were $CD44^+$ (c) while virtually 100% of $ABCG2^+$ Du145 cells were harbored in the $CD44^+\alpha 2\beta^+$ cell population (e). A summary Venn diagram was presented in f. (B) LAPC9 cells were acutely purified from xenograft tumors and used in flow analysis of CD44 and integrin α2β1 expression, in which $CD44^+\alpha 2\beta^+$ cells constituted 19.4% of the total (a) whereas the $ALDH^+$ populations represented ~25% (b). Approximately 53% of the $ALDH^+$ population was $CD44^+\alpha 2\beta^+$ (c) whereas only ~4% of $ALDH^+$ cells were $ABCG2^+$ (d). Nearly 100% (i.e., 53.3% + 46.3% = 99.6%) of $ALDH^+$ cells were $CD44^+$ (c) while 98% $ABCG2^+$ Du145 cells were harbored in the $CD44^+\alpha 2\beta^+$ cell population (e). A summary Venn diagram was presented in f.

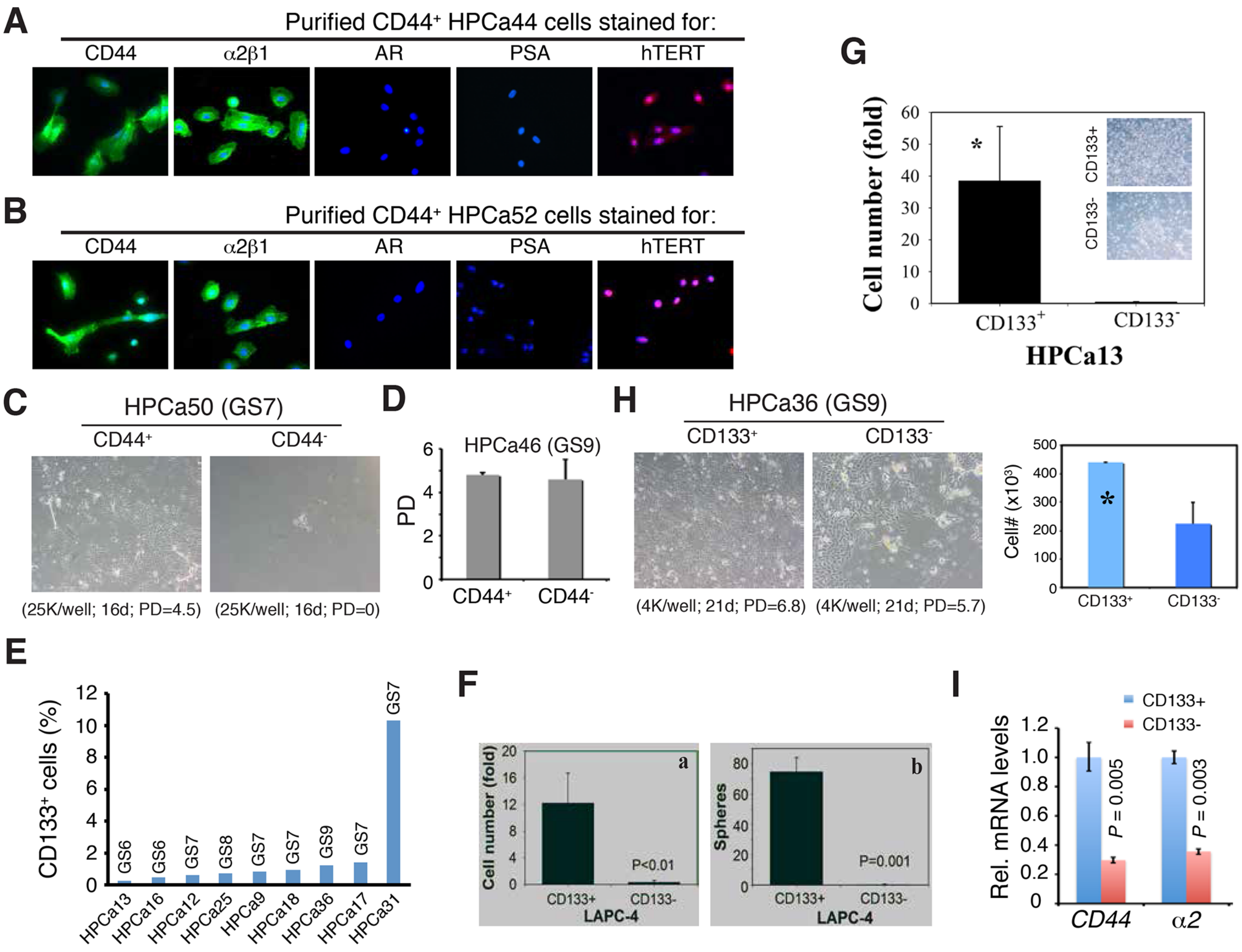

Supplementary Figure 13: *In vitro* studies on CD44⁺ and CD133⁺ HPCa cells. A–B. Highly FACS-purified primary CD44⁺ HPCa44 (A) and HPCa52 (B) cells were plated on collagen in serum-free PrEGM supplemented with a cocktail of mitogens (37, 38) overnight followed by IF labeling of the molecules indicated. Shown are representative images (original magnification: ×400) of the overlay of DAPI (for nucleus) and the indicated markers. Note that all CD44⁺ HPCa cells stained positive for CD44, α2β1, and hTERT but negative for AR and PSA. **C.** CD44⁺ HPCa50 cells possess higher survival advantages compared to the corresponding CD44⁻ cells. Purified CD44⁺ and CD44⁻ HPCa50 cells were plated in quadruplicate on collagen-coated 6-well dishes. Shown below are the cell numbers plated, time when surviving cells were enumerated, and the cumulative PDs. In this experiment, few CD44⁻ HPCa50 cells survived. **D.** CD44⁺ and CD44⁻ HPCa46 cells exhibit similar proliferative potential. Purified CD44⁺ and CD44⁻ HPCa46 cells were plated in triplicate on collagen-coated 6-well dishes (10,000 cells/well). Live cell numbers were determined 23 days after plating. Shown are the cumulative PDs (mean ± S.D). **E.** CD133 expression (%) in untreated HPCa samples. The combined Gleason score (GS) of patient tumors is indicated. **F.** The CD133⁺ LAPC4 cells possess higher proliferative and sphere-forming potentials than corresponding CD133⁻ cells. 15, 000 CD133⁺ or CD133⁻ LAPC4 cells freshly purified from xenograft tumors were plated in triplicate in 6-well plates coated with collagen (a) or a layer of agarose (b). Cell numbers (a) or spheres (b) were determined 3 weeks after plating. **G.** CD133⁺ (2, 000) and CD133⁻ (100, 000) HPCa13 cells were freshly purified out using MACS and plated, in quadruplicate, on collagen-coated dishes and cultured in 1% O2. Cell number (expressed as fold increase) was enumerated 3 weeks after plating. *$P < 0.001$. Shown in the inset are representative microphotographs (original magnification: ×100). **H.** CD133⁺ and CD133⁻ HPCa36 cells were freshly purified out using MACS and plated, in quadruplicate, on a feeder layer of Swiss 3T3 cells in 12-well culture plates (4, 000 cells/well). Cell number was determined 3 weeks after plating and bars represent the mean ± S.D (right; *$P < 0.01$). Shown on the left are representative microphotographs (original magnification: ×100) and the cumulative PDs. **I.** CD133⁺ HPCa40 cells also overexpress *CD44* and integrin *α2* mRNAs. Primary tumor HPCa40 (Gleason 7) was used to purify out Lin⁻CD133⁺ and Lin⁻CD133⁻ PCa cells, which were analyzed, by qPCR, for *CD44* and *α2* mRNAs. *P* values (Student *t*-test) are indicated.

Supplementary Table S1: *Oncomine* data sets analyzed in the current study

Supplementary Table S2: HPCa and HPCa(N) samples used in this study*

Patient	Age	Gleason	Stage	Marker(s) analyzed (%)$					Experiments
				CD133	ALDH	CD44	α2β1	CD44α2β1	
HPCa4	63	7 (4+3)	N/A			17.8			Marker quantification
HPCa6	68	7 (3+4)	T2a, N0, MX			4.0			Marker quantification
HPCa7	66	7 (4+3)	N/A			15.0			Marker quantification
HPCa8	56	6 (3+3)	T2c, NX, MX			12.4			Marker quantification
HPCa9	62	7 (4+3)	N/A	0.84					Marker quantification
HPCa10	71	6 (3+3)	T2a, NX, MX			3.3			Marker quantification
HPCa12	59	7 (4+3)	N/A	0.61					Marker quantification; AR/PSA IF
HPCa13	62	6 (3+3)	N/A	0.25					$CD133^{+/-}$ clonal assay
HPCa14	68	7 (4+3)	T3, N1, MX			5.63			Marker quantification: AR/PSA IF
HPCa16	58	6 (3+3)	pT3a, pN0, pMX	0.46					Marker quantification
HPCa17	60	7 (4+3)	T2c, N0, MX	1.4					Marker quantification
HPCa19	61	9 (4+5)	pT3b, NX, MX, G4						AR/PSA IF
HPCa18	68	7 (4+3)	N/A	0.94		19.4			Marker quantification; AR/PSA IF
HPCa25	58	8 (3+5)	pT2c, NX, MX, G4	0.73		7.7			Marker analysis
HPCa27	47	8 (4+4)	pT3a, pNX, pMX, G4, R1			4.8			Marker quantification; AR/PSA IF
HPCa31	63	7 (3+4)	N/A	10.3		71.4			Marker quantification; AR/PSA IF
HPCa33	59	7 (3+4)	pT2, pNX, pMX, G3, R1						AR/PSA IF

(*Continued*)

Patient	Age	Gleason	Stage	Marker(s) analyzed (%)[$]					Experiments
				CD133	ALDH	CD44	α2β1	CD44α2β1	
HPCa34	58	7 (4+3)	N/A			5.5			Marker quantification
HPCa36	65	9 (4+5)	pT3b, pR1, pN0, pMX	1.23					Marker quantification
HPCa37	59	9 (4+5)	pT3b, R1, pNX, pMX						AR/PSA IF
HPCa38	63	9 (4+5)	pT3b, RpNX, pMX						AR/PSA IF
HPCa39	63	7 (3+4)	pT3a, NX, MX, G3–4			56.5			Marker quantification
HPCa40	58	7 (3+4)	pT2a, pNX, pMX						qPCR CD44, Φ2, and CD133
HPCa41	49	9 (4+5)	pT2a, pNX, pMX			18.6			Marker quantification
HPCa42	54	9 (5+4)	T3b, NX, MX			20.3			Marker quantification
HPCa43	55	7 (4+3)	T3a, NX, MX			13.3			Marker quantification; clonal analysis
HPCa44	61	9 (5+4)	pT3b, R, pNX, pMX			30.5			Marker quantification; clonal analysis
HPCa45	70	9 (4+5)	pT2c, R, pNX, pMX			3.6			Marker quantification
HPCa46	51	9 (4+5)	T2c, NX, MX			19.6			Marker quantification; clonal analysis
HPCa47	56	7 (4+3)	pT2c, R, pNX, pMX			5.45			Marker quantification
HPCa48	59	7 (3+4)	T2c, NX, MX			1.53			Marker quantification
HPCa49	57	7 (4+3)	T3b, R1, NX, MX			2.65			Marker quantification
HPCa50	60	7 (4+3)	T3a, NX, MX			7.9			Marker quantification; clonal, & clonogenic
HPCa51	44	7 (4+3)	pT3a, pNX, pMX, G3			6.1			Marker quantification; clonogenic analysis
HPCa52	63	8 (4+4)	pT2a			3.2			Marker quantification
HPCa53	58	7 (4+3)	T3a, NX, MX						Clonal & clonogenic assays

(*Continued*)

Patient	Age	Gleason	Stage	Marker(s) analyzed (%)$					Experiments
				CD133	ALDH	CD44	α2β1	CD44α2β1	
HPCa54	61	8 (4+4)	pT3a, R1, pNX, pMX						Clonal & clonogenic assays
HPCa57	53	7 (3+4)	pT2c, NX, MX, G3, R1						Clonal & clonogenic assays
HPCa58	58	7 (3+4)	pT3a, NX, MX, G3, R1						Clonal & clonogenic assays
HPCa60	54	8 (3+5)	T2c, NX, MX			8.7			qPCR of AR/PSA/CD44; Marker quantification#
HPCa61	56	6 (3+3)	T2c, NX, MX						qPCR of AR/PSA/CD44
HPCa62	59	7 (4+3)	pT2c, pNX, pMX			2.4			qPCR of AR/PSA/CD44; Marker quantification#
HPCa63	51	7 (4+3)	pT2c, pNX, pMX						Clonal & clonogenic assays
HPCa65	59	7 (4+3)	T3b, NX, MX			19.9			qPCR of AR/PSA/CD44; Marker quantification#
HPCa66	58	6 (3+3)	pT2c, pNX, pMX			15.0			qPCR of AR/PSA/CD44; Marker quantification#
HPCa68	64	6 (3+3)	T2c, NX, MX						Clonal & clonogenic assays
HPCa72	58	7 (3+4)	pT2a, pNX, pMX			10.2			qPCR of AR/PSA/CD44; Marker quantification#
HPCa74	59	7 (3+4)	pT2c, NX, MX			16.2			qPCR of AR/PSA/CD44; Marker quantification#
HPCa76	64	7 (4+3)	T3a, NX, MX			0.02			qPCR of AR/PSA/CD44; Marker quantification#
HPCa77	46	6 (3+3)	T2c, NX, MX			14.2			Marker quantification#
HPCa78	64	7 (3+4)	pT2c, pNX, pMX			19.2			qPCR of AR/PSA/CD44; Marker quantification#
HPCa79	67	7 (4+3)	pT3a, pNX, pMX, G3			8.2			Marker a quantification#
HPCa80	65	9 (4+5)	T2c, NX, MX			4.4			Marker quantification#
HPCa81	54	7 (3+4)	pT2c, pNX, pMX			20.9			Marker quantification#

(Continued)

Patient	Age	Gleason	Stage	Marker(s) analyzed (%)[$]					Experiments
				CD133	ALDH	CD44	α2β1	CD44α2β1	
HPCa83	69	7 (3+4)	T2a, NX, MX						AR/PSA IF
HPCa84 & HPCa84(N)	66	7 (4+3)	T3a, R1, N0, MX						Marker IHC
HPCa87	57	9 (4+5)	pT3b, pNX, pMX, G4, R1			UD			Marker quantification
HPCa89 & HPCa89(N)	55	9 (4+5)	pT3a, pN0, pMX			24			qPCR of AR/PSA/ CD44; Marker quantification[#]
HPCa91	60	8 (3+5)	pT3a, NX, MX			UD			Marker quantification
HPCa93 & HPCa93(N)	58	7 (4+3)	pT2c, pNX, pMX			0.99			Marker quantification[#]
HPCa96 & HPCa96(N)	55	9 (5+4)	T3b, R1, N1, MX						Marker IF; α2β1 cell counting
HPCa98	64	8 (4+4)	pT3a			5.74			IF of PSA and α2β1; Marker quantification[#]
HPCa101[**]	71	9 (4+5)	pT3a, NX, MX		1.5	91.5	4.1	3.9	Marker quantification
HPCa102	55	6 (3+3)	pT2c, pNX, pMX			24.8			qPCR of AR/PSA/ CD44; Marker quantification[#]
HPCa110 & HPCa110(N)	73	8 (4+4)	pT3, N0, MX						IF of PSA and CD44; α2β1 cell counting
HPCa124	65	9 (4+5)	pT2b, N0, cM0		7.2	81.4	50.4	49.7	Marker quantification
HPCa126	68	7 (3+4)	pT2c, pN0, pMX		4.4	UD	UD	UD	Marker quantification
HPCa128 & HPCa128(N)	61	7 (3+4)	T3b, N0, MX		3.6	2.4	8.3		UD IF of PSA & ALDH1A1; Marker quantification
HPCa134 & HPCa134(N)	65	7 (4+3)	pT2a, R1, pN0						Analysis of $ALDH^{+}CD44^{+}\alpha2\beta1^{+}$ cells

[*]HPCa samples were obtained from radical prostatectomy with patients' consent under IRB LAB04–0498. The # refers to de-identified patient numbers used in our lab.

HPCa(N) refers to normal (benign) tissues from the matching HPCa samples.

[$]The % marker-positive cells were determined by FACS, MACS, or immunofluorescence (IF) staining. IHC, immunohistochemistry (i.e., Marker quantification).

[#]Part of the information for these samples was presented in ref. 12.

[**]Analyzed in early xenograft tumors.

Supplementary Table S3: Marker expression and tumorigenic properties in 4 commonly used PCa cell lines

Cells	Markers (%)[a]							Clonogenicity[b] (%)	Tumorigenicity[c]	Metastasis[d]
	CD44	α2β1	CK5	Tel	CK18	AR	PSA			
**LNCaP**	–	–	–	–	+	+	+	0.5	+	+/-
**Du145**	28	5.5	22	+	+	–	–	0.9	++	++
**PC3**	99	100	90	+++	+	–	–	1.2	+++	+++
**PPC-1**	99	100	92	+++	+	–	–	1.9	+++	+++

[a]For CD44, α2β1, and CK5 staining, cells cultured on coverslips were used in immunofluorescent staining. The % of marker-positive cells was determined by randomly counting 600 – 1,000 cells. Telomerase (Tel) activity in these cells was determined as previously described (Bhatia et al., *J. Biol. Chem*. 283:27957–72, 2008) and presented as relative levels, i.e., from negative (–) to very strong (+++). For luminal cell markers (CK18, AR, and PSA), cells were qualitatively assessed in tumor cell spheres/spheroids as either positive (+) or negative (see Supplementary Figure 6).

[b]Clonogenicity was performed by plating 1,000 – 10,000 cells (depending on cell types) on a layer of solidified agarose gel and quantifying spheres that arose in 1–2 weeks (see Materials & Methods).

[c]Tumorigenicity was determined by injecting 100 – 1,000,000 cells in 50% Matrigel subcutaneously in male NOD/SCID mice and scoring tumor latency, incidence, and weight. +, ++, and +++ refer to increasing tumor development with lower numbers of cells implanted (see also Table 1).

[d]The metastatic potential of these cells was determined by implanting 100,000 or 1,000,000 GFP-labeled tumor cells in the dorsal prostate of male NOD/SCID mice and analyzing, 3–5 months after implantation (depending on cell types), the levels of GFP^+ cells that had metastasized to the lung, lymph nodes, pancreas, brain, and kidney. +/–, ++, and +++ refer to increasing levels of metastasis to these end organs. Note that the quantitative results for metastasis assays are to be presented elsewhere.

Supplementary Table S4: Different PCa models possess distinct tumorigenic subsets*

Cells	$ABCG2^+$	$CD44^+$	$α2β1^+$	$CD44^+α2β1^+$	SP^+	$ALDH^+$
Du145	+ (10×)	+ (30×)	+ (31×)	+ (2×)#	UD	+ (>60×)
PC3	–	N/A	N/A	N/A	UD	+ (4×)
LAPC9	–	+ (6–19×)	–	+ (>900×)	+ (>500×)	+ (~90×)
LAPC4	N/A	–	–	+ (5.7×)	UD	–

*Based on data in Tables 1 and 2. +, more tumorigenic compared to the corresponding marker-negative subpopulation; –, no significant difference between the marker-positive vs. marker-negative subpopulations. Numbers in parentheses indicate relative fold enrichment in tumor-initiating activity (TIF). N/A, not assayed (or not applicable as PC3 cells were nearly 100% positive for CD44 and α2β1); UD, undetectable; ALDH, Aldefluor assay.

#Did not reach statistical significance.

Supplementary Table S5: RT² Profiler™ PCR Array Human Stem Cell List of Genes*

**Stem Cell Specific Markers:**<br>***Cell Cycle Regulators:*** APC, AXIN1, CCNA2, CCND1, CCND2, CCNE1, CDC2, CDC42, EP300, FGF1, FGF2, FGF3, FGF4, MYC, NOTCH2, PARD6A, RB1.<br>***Chromosome and Chromatin Modulators:*** GCN5L2, HDAC2, MYST1, MYST2, RB1, TERT.<br>***Genes Regulating Symmetric/Asymmetric Cell Division:*** DHH, NOTCH1, NOTCH2, NUMB, PARD6A.<br>***Self-Renewal Markers:*** HSPA9B, MYST1, MYST2, NEUROG2, SOX1, SOX2.<br>***Cytokines and Growth Factors:*** BMP1, BMP2, BMP3, CXCL12, FGF1, FGF2, FGF3, FGF4, GDF2, GDF3, IGF1, JAG1.<br>***Genes Regulating Cell-Cell Communication:*** DHH, DLL1, GJA1, GJB1, GJB2, JAG1.<br>***Cell Adhesion Molecules:*** APC, BGLAP, CD4, CD44, CDH1, CDH2, COL9A1, CTNNA1, CXCL12, NCAM1.<br>***Metabolic Markers:*** ABCG2, ALDH1A1, ALDH2, FGFR1.
**Stem Cell Differentiation Markers:**<br>***Embryonic Cell Lineage Markers:*** ACTC, ASCL2, FOXA2, IPF1, ISL1, KRT15, MSX1, MYOD1, T.<br>***Hematopoietic Cell Lineage Markers:*** CD3D, CD4, CD8A, CD8B1, MME.<br>***Mesenchymal Cell Lineage Markers:*** AGC1, ALPI, BGLAP, COL1A1, COL2A1, COL9A1, PPARG.<br>***Neural Cell Lineage Markers:*** CD44, NCAM1, OPRS1, S100B, TUBB3.
**Signaling Pathways Important for Stem Cell Maintenance:**<br>***Notch Pathway:*** DLL1, DLL3, DTX1, DTX2, DVL1, EP300, GCN5L2, HDAC2, JAG1, NOTCH1, NOTCH2, NUMB.<br>***Wnt Pathway:*** ADAR, APC, AXIN1, BTRC, CCND1, FRAT1, FZD1, MYC, PPARD, WNT1.

*This stem cell gene expression qPCR panel was custom-made by the SuperArray Bioscience Corporation. The 84 genes are categorized into several different functional groups by GO terms.
